# Development of Macrocyclic PRMT5–Adaptor Protein Interaction Inhibitors

**DOI:** 10.1021/acs.jmedchem.2c01273

**Published:** 2022-11-15

**Authors:** Adrian Krzyzanowski, Lea Marie Esser, Anthony Willaume, Renaud Prudent, Christoph Peter, Peter ‘t Hart, Herbert Waldmann

**Affiliations:** † Department of Chemical Biology, Max Planck Institute of Molecular Physiology, Otto-Hahn-Straße 11, 44227 Dortmund, Germany; ‡ Faculty of Chemistry, Chemical Biology, Technical University Dortmund, Otto-Hahn-Straße 6, 44221 Dortmund, Germany; § Institute of Molecular Medicine I, Medical Faculty and University Hospital, Heinrich Heine University Düsseldorf, Universitätsstraße 1, 40225 Düsseldorf, Germany; ∥ Edelris, Bioserra 1, 60 Avenue Rockefeller, 69008 Lyon, France; ⊥ Chemical Genomics Centre of the Max Planck Society, Max Planck Institute of Molecular Physiology, Otto-Hahn-Straße 11, 44227 Dortmund, Germany

## Abstract

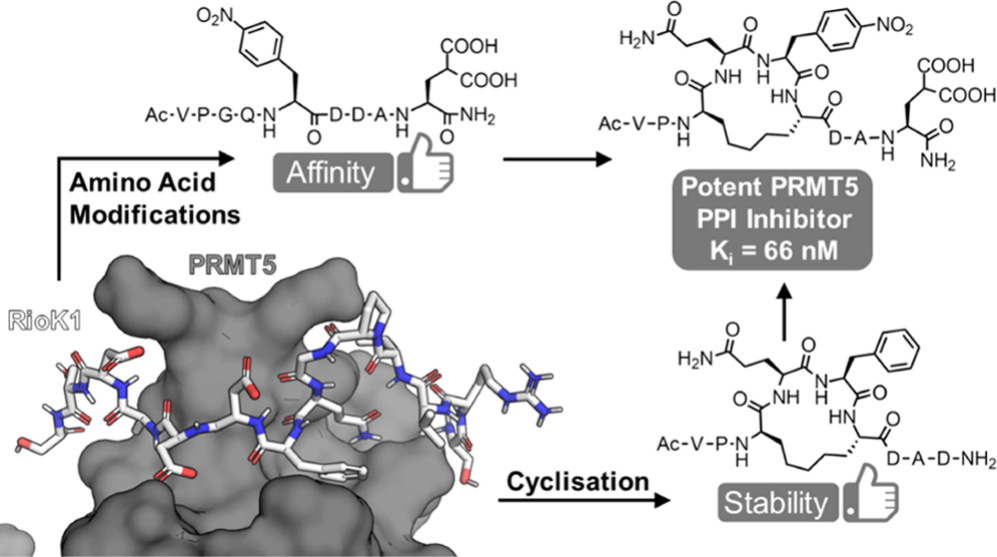

The PRMT5-MEP50 methyltransferase is a major target for anticancer drug discovery, and modulators of its interactions with different regulatory proteins are in high demand because they modulate PRMT5 substrate selectivity. We describe a strategy for the development of a PRMT5/adaptor protein PPI inhibitor, which includes the design and synthesis of macrocyclic peptides based on the motif for the interaction of PRMT5 with its adaptor protein RioK1. After the initial exploration of different macrocycle sizes and cyclization linkages, analysis of a peptide library identified hot spots for the variation of the amino acid structure. The incorporation of nonproteinogenic amino acids into the macrocyclic peptide led to a potent cyclic PRMT5 binding peptide (*K_i_* = 66 nM), which selectively inhibits the interaction of PRMT5 with the adaptor proteins RioK1 and pICln (IC_50_ = 654 nM) but not with the alternative adaptor protein MEP50. The inhibitor is a promising tool for further biological investigation of this intriguing protein interface.

## Introduction

Protein arginine methyltransferase 5 (PRMT5) is a prominent epigenetic regulator with a critical function in cellular development, growth, splicing, DNA damage response, signaling, trafficking, and other biological processes.^1^ The pivotal role of PRMT5 in diverse types of cancers and cardiovascular and neurodegenerative diseases calls for a detailed understanding of the activity, function, and regulation of this enzyme.^1,2^ Inhibition of the enzymatic activity of PRMT5 is a therapeutic strategy that is currently being explored for cancer treatment, with various compounds being tested in clinical trials.^3,4^

PRMT5 is composed of three domains: a Rossmann fold, a β-barrel, and a TIM barrel. The Rossmann fold houses the catalytic site, whereas the β-barrel allows for the dimerization of PRMT5, and the TIM barrel mediates interactions with different adaptor proteins.^5,6^ PRMT5 is normally found as a heterooctameric complex containing four copies of PRMT5 and four copies of its partner protein MEP50 (WDR77).^7,8^ It produces monomethylated and symmetrically dimethylated arginine residues on a diverse range of substrates.^9^ PRMT5’s function, activity, and substrate specificity are regulated through post-translational modifications of the enzyme, expression of several miRNAs, and interaction with pertinent adaptor proteins.^1^ These adaptor proteins include the aforementioned MEP50 aiding in histone tail methylation,^7,8,10^ RioK1, which recruits nucleolin as a substrate,^11^ pICln, which connects the enzyme to Sm proteins,^12,13^ and COPR5, which enables recruitment to chromatin and preferential methylation of histone H4.^14^

Being able to selectively modulate one of the PRMT5 PPIs would allow one to inhibit the methylation of only a subset of its targets. The feasibility of this was recently demonstrated by Asberry and colleagues, who reported the first biologically active inhibitor of PPI between PRMT5 and MEP50, highlighting the strong community interest in targeting these challenging PRMT5 interactions with the adaptor proteins.^15^

We, and independently the Sellers group, recently reported the identification and characterization of a novel PRMT5 binding motif (PBM) with the consensus sequence GQF[D/E]DA[E/D] found in the adaptor proteins pICln, RioK1, and COPR5.^5,6^ Methylation of 25 PRMT5 substrates is dependent on the interaction between the PBM and the PBM binding groove located on the TIM barrel of PRMT5.^6^ This finding suggested that a targeted inhibition of the protein–protein interactions (PPIs) at the PBM groove might allow for controlled regulation of PRMT5 substrate specificity. Very recently, the first example of this strategy has been reported, revealing a covalent PBM groove binder with moderate activity.^16^ The discovery of this first-in-class compound also highlighted the challenges associated with finding a potent binder of the groove as it included a screening of over 900,000 compounds from diverse libraries, an NMR-based fragment screen, and an *in silico* pharmacophore screen.^16^ In light of these efforts, the development of an alternative approach to finding a potent inhibitor of this challenging PPI appeared indicated.

For the development of a modulator of PRMT5/adaptor protein interactions, we decided to derive an inhibitory peptide from the binding epitope of the PPI. In particular, it was envisaged to develop cyclopeptides as they are known to be especially effective at inhibiting PPIs involving large surface areas and poorly defined pockets.^17−19^ Grafting inhibitory macrocyclic peptides out of a well-defined PPI, for example, by means of peptide stapling, can be highly effective.^20−22^ A cyclic peptide tends to be a more useful tool than its linear counterpart due to potential improvements in potency and proteolytic stability.^23^

We now report a stepwise strategy for the development of a potent macrocyclic PRMT5–adaptor protein interaction inhibitor (PAPII). Macrocyclization was explored and optimized, significantly improving the peptide stability. Various amino acid substitutions were explored, increasing the affinity of the inhibitor for the PBM groove, and the resulting compound was investigated for disruption of the PRMT5–adaptor protein PPI, showing effective modulation of the interaction.

## Results and Discussion

**Figure 1:**
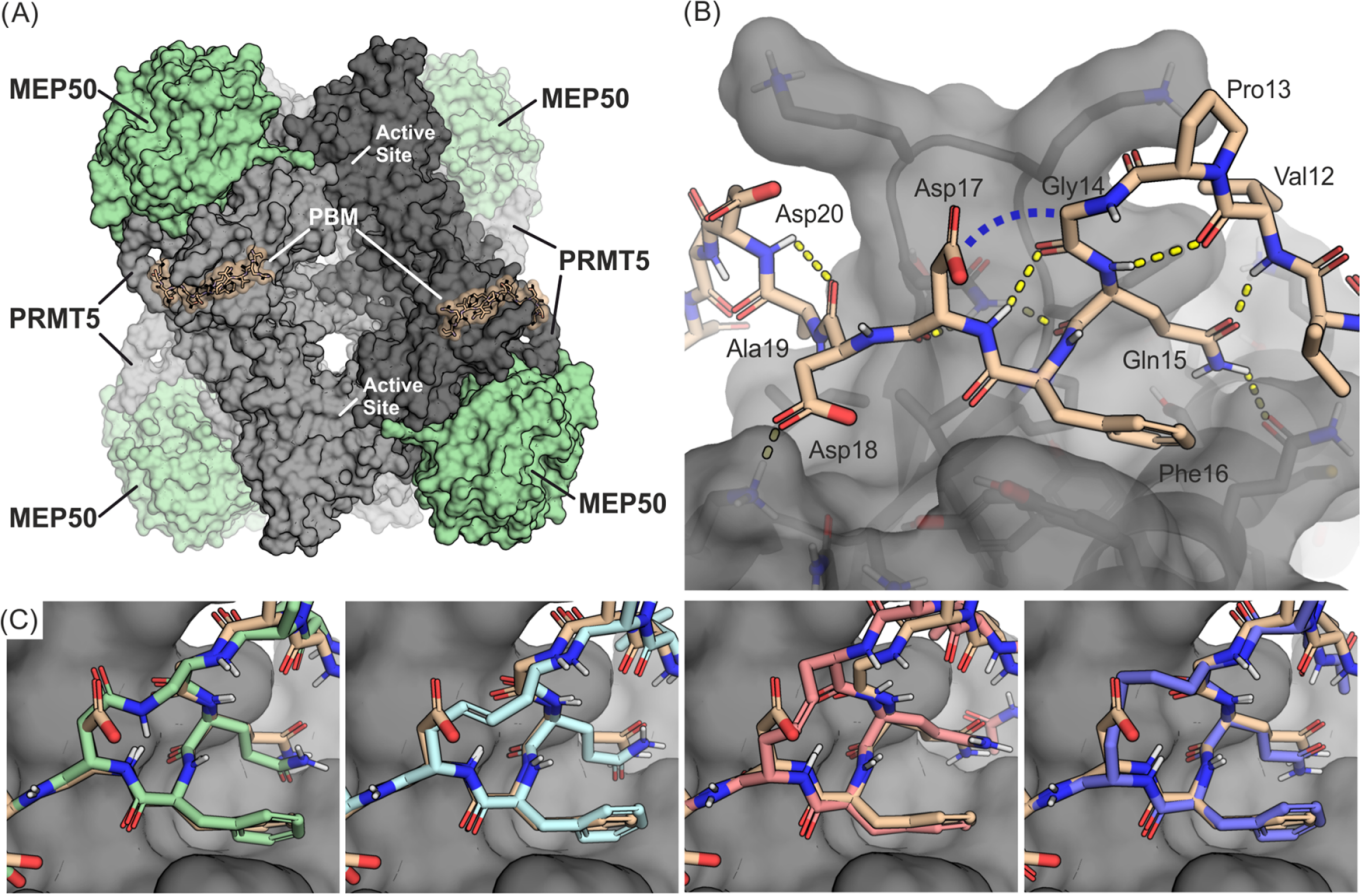
(A) Heterooctameric PRMT5-MEP50 complex with PBM peptides bound to the TIM barrel domains. The full PRMT5-MEP50 complex can bind four PBM peptides (one per PRMT5 protomer) and has four active sites (one per PRMT5 protomer). The image is shown as a superposition of the PDB structures 7BOC and 4GQB. (B) Complex of the TIM barrel with a RioK1-derived peptide bound to the PBM groove (PDB ID/7BOC). The blue dashed line indicates the envisaged macrocyclization site. (C) Computational models of exemplary macrocycles overlaid on the linear peptide structure (wheat). A macrocycle with a 4-atom-long amide linker (**5**, green, first panel), a 4-atom-long unsaturated RCM linker (**13** in trans, blue, second panel and cis, pink, third panel), and a 5-atom-long saturated linker (**21**, purple, fourth panel).

**Figure 2:**
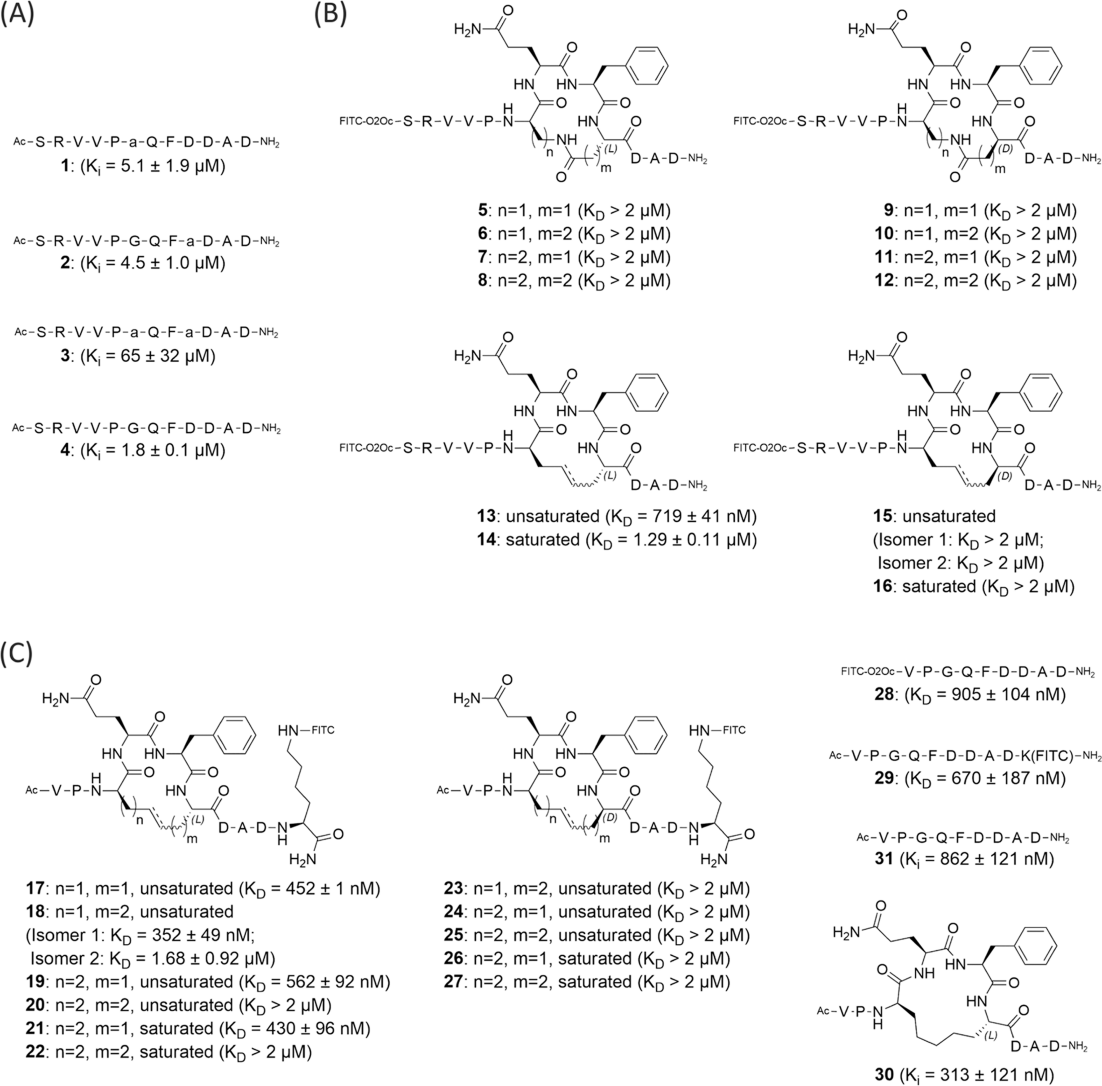
(A) Peptides incorporating d-Ala into envisaged cyclization positions of RioK1 PBM. Peptides were tested for competitive binding to PRMT5-MEP50 in the presence of a fluorescently labeled SRVVPGQFDDADSSD RioK1 sequence. Peptide **4** was used as a reference. a = d-Ala. (B) The first generation of cyclic peptides with amide- and RCM-based linkers. The provided *K*_D_ values were measured for binding to PRMT5-MEP50. (C) The second generation of the cyclic peptides based on the RCM linkers. The *K*_D_ and *K_i_* values were determined for binding to the PRMT5-MEP50 complex.

Based on the co-crystal structures of the RioK1-derived PBM in complex with the PRMT5 TIM barrel (Figure 1A,B) and Ala scan results,^5,6^ we envisaged that the covalent connection of the α-carbons in Gly14 and Asp17 would be a feasible macrocyclization strategy (Figure 1B). Such a design would encompass the hot spot residues Gln15 and Phe16 and potentially stabilize the double β-turn of the PBM peptide as well as the key intermolecular H-bonds formed with PRMT5. Computationally generated models of the macrocycles suggested a need for a 4- or 5-atom-long linker (Figure 1C) as well as replacement of Gly14 with a d-amino acid and incorporation of either an l- or a d-residue in place of Asp17 for the correct orientation of the side chains. To evaluate whether such modifications were tolerated, compounds **1–3** were prepared and tested in a competitive fluorescence polarization (FP) assay, comparing the modified peptides with the native sequence **4** (Figures 2A and S1). Introduction of d-Ala, at either position 14 or 17 (compounds **1** and **2**), resulted in a small decrease in the peptide affinity, whereas simultaneous replacement of both Gly14 and Asp17 with d-Ala caused a significant weakening of the binding (compound **3**), suggesting that only a single d-amino acid was preferred. Connection of the side chains of these amino acids by amide bond formation or by ring-closing metathesis (RCM) resulted in the first generation of macrocyclic PAPIIs (compounds **5–16**, Figure 2B).^24^ The cyclopeptides were prepared with an attached fluorescein isothiocyanate (FITC) tag to enable direct measurement of interactions with the PRMT5-MEP50 complex and, thus, allow for the identification of the *K*_D_ values for the examined molecules.

**Scheme 1:**
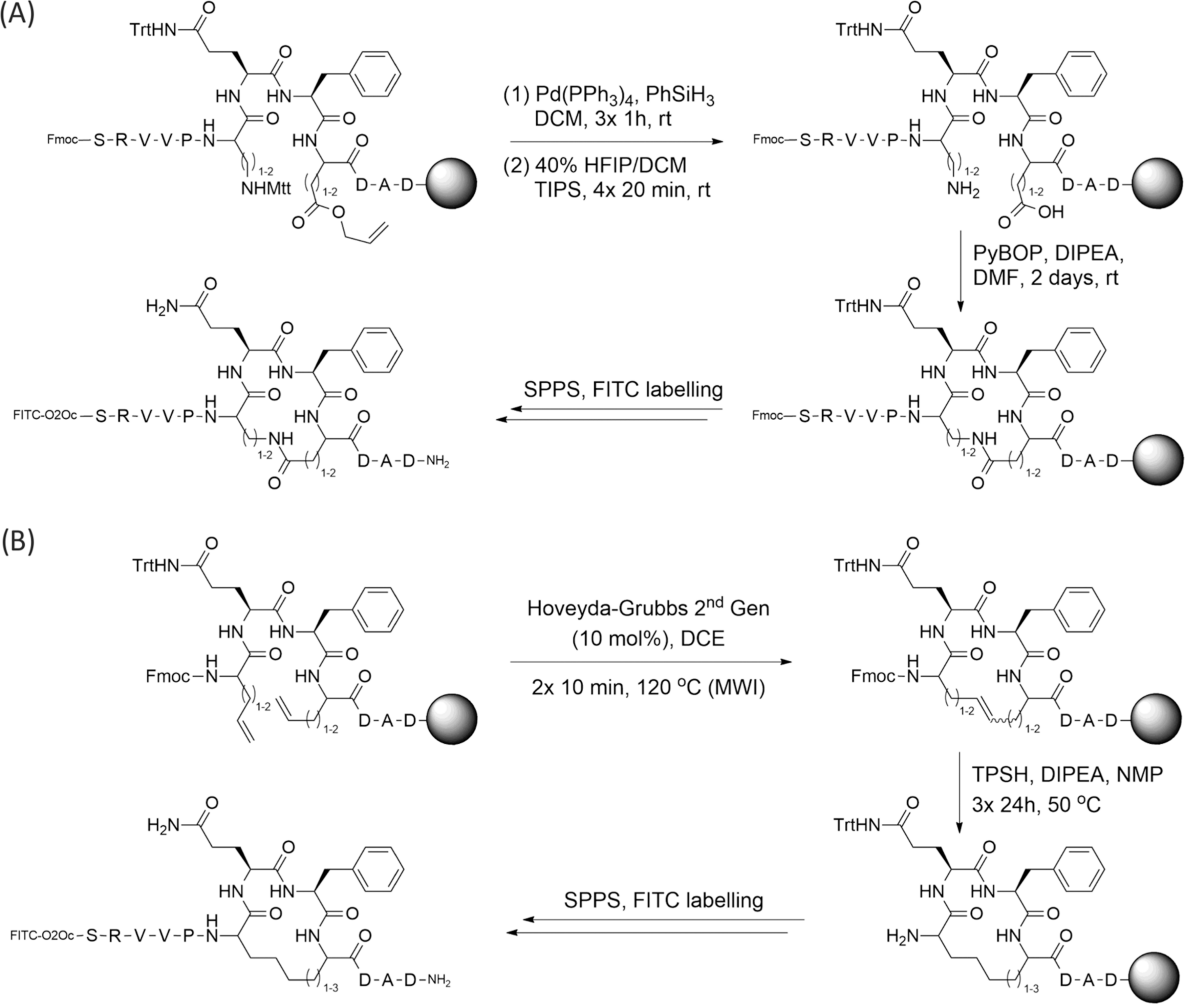
(A) General Peptide Synthesis Scheme for Macrocycle Formation through an Amide Bond Linker. (B) General Peptide Synthesis Scheme for Macrocycles Formed by Ring-Closing Metathesis. For Peptides with a Double Bond, the TPSH Reduction Step was Omitted

The fluorescently labeled cyclic peptides were synthesized on-resin using an Fmoc-based strategy with macrocycles spanning 14 to 16 atoms. For macrocyclization by means of amide bond formation, peptides were synthesized using orthogonal protection of diaminobutyric acid (Dab)/diaminopropionic acid (Dap) side chains with the acid labile Mtt group and the allyl group for Asp and Glu, respectively. Selective side chain deprotection with 40% hexafluoroisopropanol (HFIP) for Mtt and treatment with Pd(PPh_3_)_4_ in the presence of phenylsilane for allyl group removal was followed by a PyBOP-induced cyclization (Scheme 1A). In the case of allyl-protected Asp, it was necessary to protect the preceding amide bond with the dimethoxybenzyl (Dmb) protecting group due to significant aspartimide formation (Scheme S1). For cyclization by ring-closing metathesis, the Hoveyda–Grubbs 2nd generation catalyst was employed with microwave irradiation and, where appropriate, followed by reduction of the formed double bond using 2,4,6-triisopropylbenzenesulfonyl hydrazide (TPSH, Scheme 1B). Where possible, *cis* and *trans* isomers were isolated and tested individually.

Investigation of peptides **5–16** for binding to PRMT5-MEP50 by FP measurements (Figures 2B and S2) revealed that peptide **13**, with a double bond embedded in a 4-atom-long linker, displayed the highest affinity (*K*_D_ = 719 ± 41 nM). Compounds with a d-residue in place of Asp17 did not show any binding to the protein, confirming the findings for peptide **3**. Although moderate binding affinity was observed for some of the amide-cyclized compounds (Figure S2), the results suggested that cyclization via ring-closing metathesis yields better inhibitors and that optimization of the linker length is crucial. For further inhibitor design, we experimented with the truncation of the N-terminal SRV motif and shifting the FITC label to the C-terminus (compounds **17–27**, Figures 2C, S3 and Scheme S2). The decision to change the location of the fluorescent tag in the shortened structures was prompted by the comparison of the affinities toward PRMT5-MEP50 between linear peptides **28** (*K*_D_ of 905 ± 104 nM) and **29** (*K*_D_ of 670 ± 187), which suggested a potential steric interference caused by the N-terminally placed tag during binding to the protein (Figures 2C and S4). The influence of linker length in the rings of the cyclopeptides was explored using combinations of terminal alkene amino acids with varying side chain lengths and differing stereochemistry. Peptides **18** and **21**, which contain 5-atom linkers, were identified as the most potent cyclic molecules with similar *K*_D_ values of 352 ± 49 nM (for one of the isolated isomers) and 430 ± 96 nM, respectively (Figures 2C and S3), giving a slight affinity improvement over the linear analogue **29** (*K*_D_ of 670 ± 187 nM, Figure 2C). As only one of the very difficult-to-isolate isomers of **18** shows a strong affinity for the target, we decided to continue the investigations using the saturated scaffold of **21**, allowing us to significantly improve the amounts of the obtained material needed for testing. As predicted by the computational models, only compounds with 4–5 atom linkers were able to bind potently, while the 6-atom linker containing macrocycles was less effective. Comparison of unlabeled macrocycle **30** (Figure 2C) and the linear equivalent **31** in a competitive FP assay (using compound **21** as a tracer) revealed an IC_50_ of 1.17 ± 0.29 μM for cyclic **30** and an IC_50_ of 2.49 ± 0.22 μM for linear **31** (Figure S5). These values translate to a *K_i_* of 313 ± 121 nM for **30** and a *K_i_* of 862 ± 121 nM for **31**, which are comparable to the *K*_D_ values obtained for the fluorescently labeled analogues **21** and **29**. The competitive binding results confirm that the linear and cyclic peptides interact with the same PRMT5 site and prove that the fluorophore does not interfere with binding.

**Figure 3:**
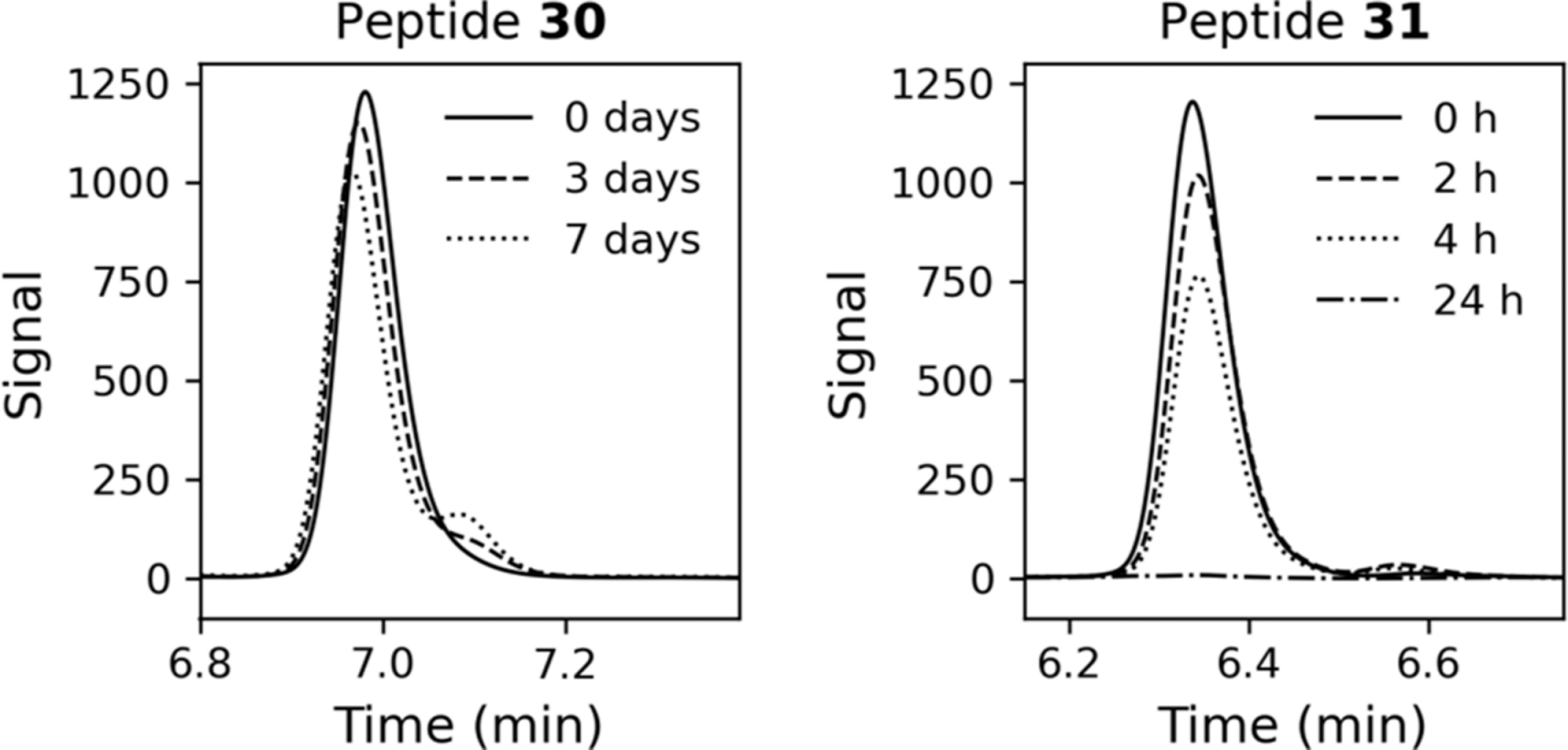
HPLC analysis of cyclic **30** and linear **31** incubated in U2OS cell lysate at 37 °C. Presented time points: 0–7 days for **30** and 0–24 h for **31**.

For potential application in biological investigations, peptides need to be resistant to proteolytic digestion for relevant time frames. To evaluate whether cyclization improved proteolytic stability, we exposed the unlabeled macrocycle **30** to a cell lysate at 37 °C, which revealed that the peptide was very stable with a half-life of 12.5 days, a 70-fold increase over the linear **31**, which had a half-life of 4.4 h (Figures 3, S6, and S7). Further examination in an assay for inhibition of the enzymatic activity of PRMT5 proved that, as intended, cyclopeptide **30** does not interfere with the direct methylation activity of the enzyme (Figure S8).

**Figure 4:**
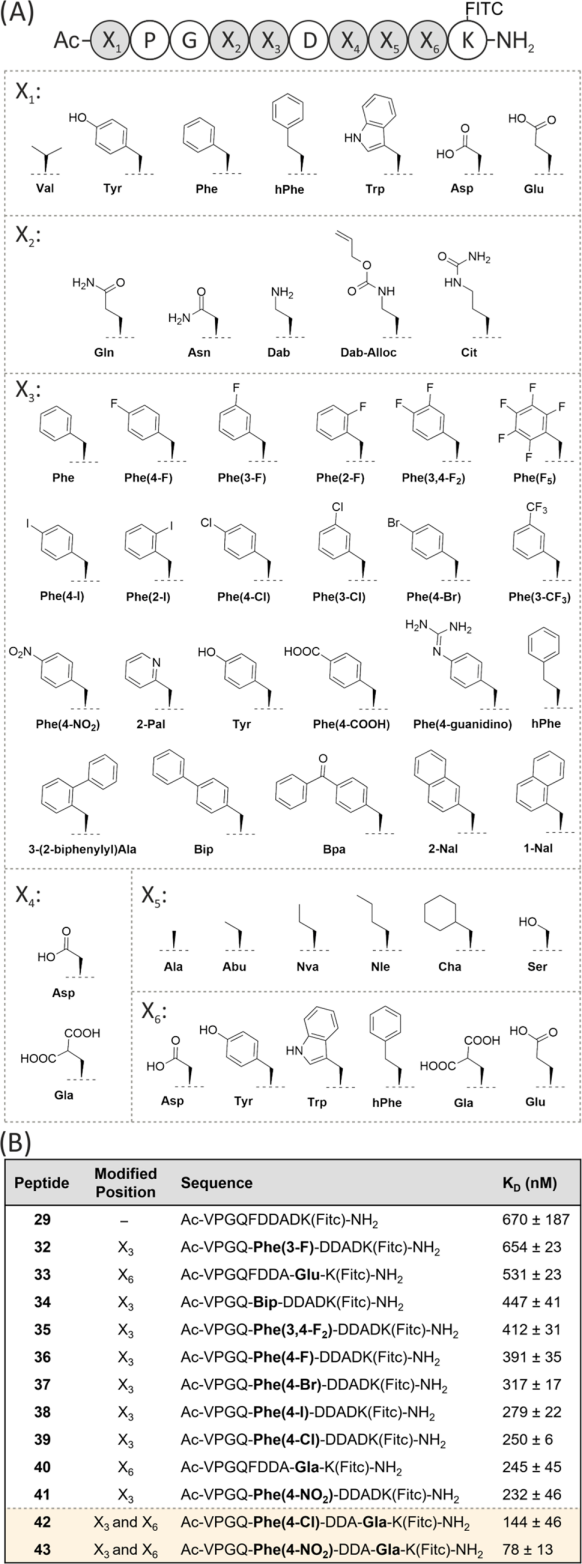
(A) Six residues in peptide **29** (X_1_–X_6_) were modified one at a time and replaced with one of the listed amino acids. (B) List of modified sequences with improved affinity for PRMT5-MEP50 over **29**.

To further improve the affinity of the PAPIIs, we designed 43 different modifications to the peptide side chains guided by the crystal structure, incorporating both proteogenic and nonproteogenic amino acids into the linear sequence of **29** (Figure 4A and Table S1). The compounds were tested by means of FP with the full-length PRMT5-MEP50 complex, which identified 10 modifications to Phe16 and Asp20 that improved the affinity over the native sequence of **29** (Figures 4B and S9–S12). The most potent was the replacement of Asp by Gla (**40**) and Phe by Phe(4-NO_2_) (**41**), increasing the affinity by three-fold in each case. The combination of these modifications in compound **43** resulted in a synergistic effect, gratifyingly affording a *K*_D_ of 78 ± 13 (Figures 4B and S9).

Backbone *N*-methylation of a linear peptide can induce a favorable conformation for binding to the target protein.^25,26^ Exploration of *N*-methylation of the amide bonds not involved in the intramolecular H-bonds but stabilizing the double β-turn (compounds **44–49**) yielded no meaningful improvement in the affinity (Table S2 and Figure S13).

**Figure 5:**
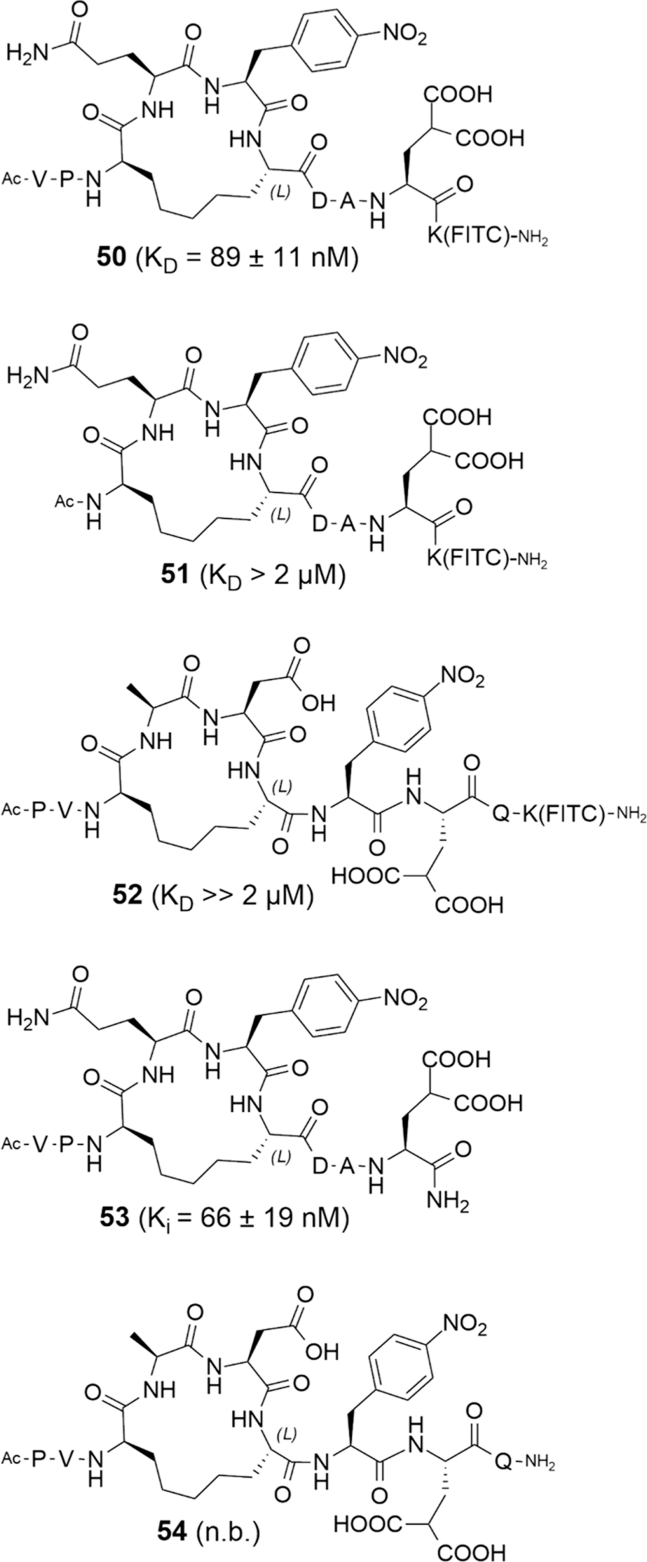
Third generation of macrocycles based on the optimized RCM linker, incorporating the identified amino acid modifications. Peptides **52** and **54** are scrambled controls. The *K*_D_ and *K_i_* values were determined for binding to PRMT5-MEP50. n.b. = no binding.

**Figure 6:**
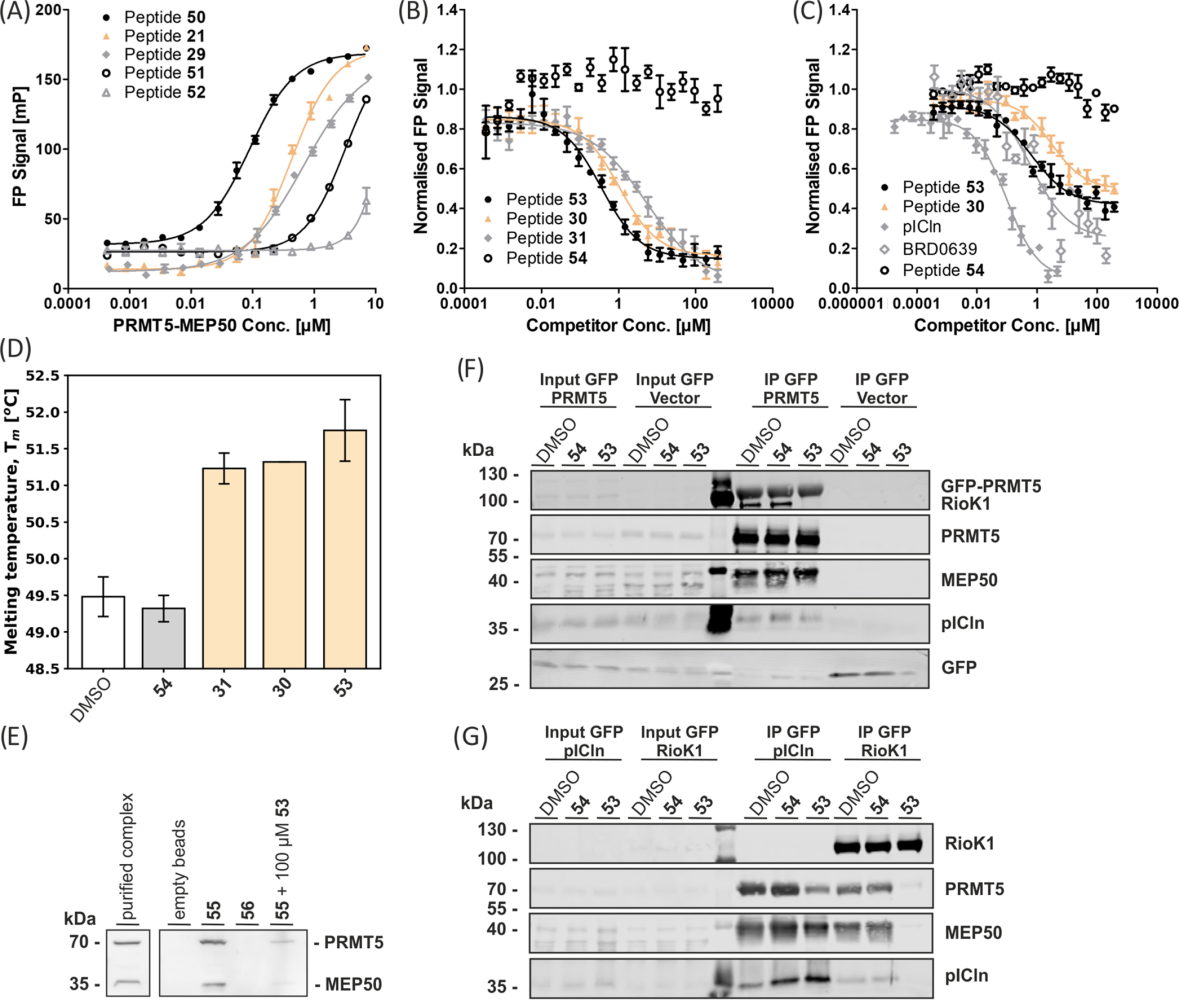
(A) FP results for linear sequence **29** and 2nd and 3rd generation macrocyclic PAPIIs with PRMT5-MEP50 (*n* = 3). (B) FP competition of representative linear and macrocyclic peptides with FITC-labeled **50** for binding to PRMT5-MEP50 (*n* = 3). (C) FP competition of 2nd and 3rd generation PAPIIs and the unlabeled pICln protein with the Alexa 488-labeled full-length human pICln protein for binding to PRMT5-MEP50 (*n* = 3). (D) Comparison of the effects of linear and cyclic peptides on the melting temperature of the TIM-MEP50 complex, as determined by TSA (*n* = 6). (E) Pull-down assay with peptides **55** and scrambled **56** immobilized on the DBCO beads using MCF7 cell lysate. Immobilized **55** was also tested with the lysate containing 100 μM of free **56**. (F) GFP-IP from the lysate of Flp-In T-REx 293-GFP and Flp-In T-REx 293-GFP-PRMT5 overexpressing cells, testing compound **53** and scrambled **54** at 50 μM. (G) GFP-IP from Flp-In T-REx 293-GFP-pICln and Flp-In T-Rex 293-GFP-RioK1 cytoplasmic extract, testing **53** and **54** at 50 μM.

The combination of all beneficial modifications into one macrocyclic PAPII molecule **50** afforded a *K*_D_ of 89 ± 11 nM for PRMT5-MEP50 (Figures 5 and 6A). Removal of the N-terminal Val and Pro to give shortened cyclic peptide **51** (*K*_D_ > 2 μM) proved that the N-terminal motif is indispensable (Figures 5 and 6A). Thus, **50** is the most advantageous PAPII. Comparison of unlabeled peptide **53** (Figure 5) with **30** and the linear equivalent **31** in a competitive FP assay (using compound **50** as a tracer) afforded an IC_50_ of 356 ± 59 nM for **53**, 1.06 ± 0.24 μM for **30**, and 5.3 ± 3.7 μM for linear compound **31** (Figure 6B). The *K_i_* value for **53** determined based on this experiment is equal to 66 ± 19 nM.

Characterization of the interaction of **53** with the PRMT5 TIM barrel-MEP50 protein complex by means of a thermal shift assay (TSA) revealed a clear stabilization effect of **53**, increasing the average melting temperature of the analyzed complex by 2.27 °C. No stabilization was observed for the scrambled analogue **54** (Figure 6D). To determine whether the optimized PAPII design enabled inhibition of the interaction between full-length human pICln and PRMT5 proteins, cyclopeptide **53** was subjected to a competitive assay employing fluorescently labeled pICln and native PRMT5-MEP50. Indeed, cyclopeptide **53** inhibits this strong PPI with an IC_50_ value of 654 ± 476 nM, whereas scrambled control peptide **54** did not compete with pICln (Figure 6C). The known covalent inhibitor BRD0639 was synthesized based on the protocol by McKinney et al. and also tested against the fluorescently labeled pICln, with the apparent IC_50_ measured after the incubation time of 1 h 45 min.^16^ The apparent IC_50_ under the assay conditions was comparable to the result obtained with cyclopeptide **53** and was equal to 568 ± 284 nM (Figure 6C).

To show that macrocyclic peptide **53** targets the PRMT5-MEP50 complex also under biologically relevant conditions, analogue **55** (Figure S14) that contains an azide at the C-terminus was synthesized and immobilized on dibenzocyclooctyne (DBCO) beads via a copper-free 1,3-dipolar cycloaddition reaction. Beads loaded with **55** were subsequently incubated with MCF7 cell lysate using empty beads and the immobilized scrambled peptide **56** as controls. Immobilized peptide **55** successfully enriched the PRMT5-MEP50 complex, while the negative controls did not show significant interaction (Figures 6E and S15). Notably, affinity enrichment from lysate in the presence of 100 μM nonimmobilized **53** demonstrated competition between the immobilized and free peptide for binding to PRMT5-MEP50. To further investigate the inhibition of the targeted PPI, we overexpressed GFP-PRMT5 in Flp-In T-REx 293 cells, followed by immunoprecipitation (IP) using an anti-GFP antibody and analysis of the enriched proteins. The experiment was performed in the presence of either **53**, **54**, or a DMSO-only control and showed, on the one hand, enrichment of PRMT5 and MEP50 in all cases, but on the other hand, the enrichment of RioK1 was blocked by **53** (Figures 6F and S16). Surprisingly, the binding of pICln to the complex was not inhibited, potentially indicating another site of interaction not targeted by **53**, as was also previously suggested in the literature.^5,13^ To confirm these findings, we overexpressed GFP-pICln and GFP-RioK1 and used an anti-GFP antibody to enrich these proteins and their binding partners. Again, **53** fully inhibited RioK1 from interacting with the PRMT5-MEP50 complex, but pICln still engaged with it (Figures 6G and S16). The physicochemical properties of **53** are likely to provide it with poor cellular uptake, and therefore, future plans are to improve on this. Once suitable cell permeability has been achieved, the compound can be tested to evaluate the effect of potent PRMT5-RioK1 interaction inhibition in cellular systems.

## Conclusions

Based on previously reported structural data of the RioK1 peptide bound to the PBM groove on PRMT5,^5,6^ we designed and identified the first macrocyclic inhibitors for PRMT5/adaptor protein interactions. By employing peptide cyclization through hydrocarbon linkers, the stability of the cyclopeptide compared to the linear analogue was increased 70-fold while maintaining binding affinity. The affinity of the cyclopeptides was significantly improved by introducing nonproteinogenic amino acids. The most potent macrocyclic peptide (**50**) binds PRMT5 with a *K*_D_ of 89 nM. Unlabeled peptide **53** inhibits the interaction between PRMT5 and pICln with an IC_50_ value of 654 nM and exhibits a clear stabilization effect on the TIM-MEP50 complex, as determined by TSA. The interaction of the cyclopeptide with PRMT5-MEP50 was further confirmed by affinity enrichment of the complex from cell lysate, and the inhibitory activity was corroborated in an IP assay with the PRMT5-MEP50/adaptor protein complexes revealing a surprising selectivity for inhibition of RioK1 over pICln. These findings indicate that the structural characterization of the PRMT5-pICln interaction might be incomplete. Our results demonstrate that peptide macrocyclization is a valid strategy for the development of selective PRMT5/adaptor proteins inhibitors, which will enable future biological investigations of this interface.

## Experimental Section

### General Methods

All chemical reagents and solvents, unless stated otherwise, were purchased from commercial suppliers: Sigma-Aldrich, Merck, Novabiochem, Acros Organics, abcr, Activate Scientific, Fluorochem, Carbolution Chemicals, Carbosynth, Iris Biotech, Carl Roth, TCI Deutschland, Fisher Scientific, Fisher Chemicals, Biosolve, Chem-Impex, Serva Electrophoresis, Gerbu Biotechnik, Calbiochem, Thermo Scientific, or VWR International. All air- and moisture-sensitive reactions were performed under an inert atmosphere of Ar gas. Organic solvents for moisture-sensitive reactions were dried through storage over activated 3Å molecular sieves for at least 24 h.

The ^1^H NMR spectral data were recorded on a Bruker DRX400 (400 MHz) spectrometer (Bruker Corporation). The δ values were referenced to the residual solvent signals of CDCl_3_ (7.26 ppm) and DMSO-*d*_6_ (2.50 ppm) as an internal standard.^27^ LR-MS data for small organic molecules were collected using a 1290 Agilent Infinity UHPLC system (Agilent Technologies) equipped with an Agilent Zorbax Eclipse Plus C18 Rapid Resolution HD 2.1 mm × 50 mm, 1.8 μm column. The automated peptide synthesis was performed using a Syro I peptide synthesizer (Multisyntech GmbH, Germany). The RCM reactions were performed in a CEM Discover microwave reactor (CEM Corporation). Preparative scale HPLC purification was carried out either on an Agilent Infinity or Infinity II liquid chromatography–mass spectrometry (LC–MS) system equipped with a 125 mm × 21 mm, 5 μm or 125 mm × 10 mm, 5 μm Macherey-Nagel Nucleodur C18 Gravity column (Macherey-Nagel GmbH & Co. KG, Germany) and detection at 210 nm. The purity of the final peptide products was determined at 210 nm using an Agilent Infinity HPLC system with a 50 mm × 3 mm, 1.8 μm Macherey-Nagel Nucleodur C18 Gravity column, with a flow rate of 0.56 mL/min, using the elution system 5% → 65% MeCN (0.1% TFA) in H_2_O (0.1% TFA) over 14 min (Method A). Alternatively, an Agilent Infinity II HPLC system was used, which was equipped with a 150 mm × 2.1 mm, 2.7 μm Agilent InfinityLab Poroshell 120 EC-C18 column, with a flow rate of 0.4 mL/min and the elution system 5 → 95% MeCN (0.1% TFA) in H_2_O (0.1% TFA) over 20 min (Method B). The purity of small molecules was confirmed by NMR. The purity of the analyzed compounds was determined to be ≥95%. The samples obtained from the stability assay were analyzed on the Agilent Infinity system at 210 nm, using a 125 mm × 3 mm, 5 μm Macherey-Nagel Nucleodur C4 Gravity column at a flow rate of 1 mL/min, with the elution system 5 → 65% MeCN (0.1% TFA) in H_2_O (0.1% TFA) over 14 min. HRMS analyses were performed on an LTQ Orbitrap mass spectrometer (Thermo Fisher Scientific) using electrospray ionization.

Concentrations of PRMT5-MEP50 were measured with the Bradford assay, using Protein Assay Dye Reagent from Bio-Rad (Cat. #500-0006; Bio-Rad Laboratories) and dilutions of BSA for calibration, whereas the concentration of pICln was determined using a NanoDrop 2000c spectrophotometer (Thermo Fisher Scientific). Fluorescence- and luminescence-based readouts were performed on the Spark Multimode Microplate Reader (Tecan Trading AG, Switzerland). Fluorescence-based assays were carried out in black, low-volume, round-bottom 384-well plates (ref 4514, Corning Incorporated), and the luminescence-based assays in white, low-volume, round-bottom 384-well plates (ref 4512, Corning Incorporated).

The antibodies used for the pull-down and immunoprecipitation experiments were as follows: anti-PRMT5 antibody (sc-376937, Santa Cruz Biotechnology) and anti-MEP50 (2828S, Cell Signaling Technology). The detection was performed using the fluorescent secondary antibodies: IRDye 680RD goat anti-rabbit and IRDye 800CW goat anti-mouse. The following primary antibodies were used for GFP immunopurification and immunoblotting: anti-PRMT5 (2252, CST), anti-RioK1 (NBP1-30103, Novus Biologicals), anti-MEP50 (2823, CST), anti-GFP (3H9, Chromotek), and anti-pICln (sc-393525, Santa Cruz). The detection of proteins was carried out with the following fluorescent secondary antibodies: IRDye 680LT goat anti-rabbit, IRDye 800LT goat anti-mouse, and IRDye 800CW goat anti-rat.

### Linear Peptide Synthesis

The synthesis of linear peptides was performed with Fmoc solid phase peptide synthesis (SPPS) methods as described previously in work by Krzyzanowski et al., 2021.^5^ The N-terminally methylated peptide **49** was synthesized following the protocols by Kim et al., 2014, to avoid the truncation of the N-terminal amino acid.^28^

### Cyclic Peptide Synthesis

#### General Approach

The synthesis of the linear peptide intermediates for cyclization was achieved through the standard Fmoc SPPS on a polystyrene-based Rink Amide AM resin LL (100–200 mesh). Resin substitution was ca. 0.35 mmol/g for the peptides cyclized through an amide bond or 0.16–0.19 mmol/g for the peptides cyclized through RCM. Peptides were synthesized manually, where each coupling step was performed using 4 equiv of amino acid, 4 equiv of PyBOP, and 8 equiv of *N*,*N*-diisopropylethylamine (DIPEA) over 1 h at rt, or in the case of the nonproteinogenic amino acids with 2 equiv of the amino acid, 2 equiv of PyBOP, and 4 equiv of DIPEA over 2 h at rt. Completeness of the coupling steps was monitored either by performing the Kaiser test on a small portion of the resin or by cleaving a portion of the intermediate from the resin and analyzing the sample with LC–MS. In the case of an incomplete coupling, the reaction was repeated. Fmoc removal was done with 20% piperidine solution in DMF, containing 0.5M Oxyma Pure for 5 min and then 10 min at rt. Acetylation of the resin-bound peptides was done using Ac_2_O (10 equiv) and DIPEA (12 equiv) in DMF over 30 min at rt. Peptides were cyclized either through the formation of an amide bond between side chains or through RCM, which was followed by the completion of the desired sequence through SPPS. Where applicable, the Mtt group was selectively removed by the treatment of the resin-bound peptides with 40% HFIP solution in DCM containing 2.5% TIPS, 4 × 20 min at rt. Fmoc-AEEA (4 equiv) was double-coupled to the peptide with PyBOP (4 equiv) and DIPEA (4 equiv) in DMF at rt over 2 h. Labeling was done with FITC isomer I (2 equiv) in the presence of DIPEA (4 equiv) in DMF over 1 h at rt and repeated overnight. Coupling to N_3_-AEEEA (3 equiv) was achieved using COMU (2.7 equiv), Oxyma Pure (3 equiv), and DIPEA (6 equiv) overnight at rt. Cleavage from the resin and the global side chain deprotection were achieved by the treatment of the resin with TFA/H_2_O/DODT/TIPS (90:5:2.5:2.5 v/v) over 2 h at rt, followed by trituration and washing in cold Et_2_O, lyophilization, and purification with preparative HPLC, where MeCN + 0.1% TFA or MeOH + 0.1% TFA (for difficult-to-isolate peptides) and H_2_O + 0.1% TFA were used as buffers.

#### Amide Bond-Mediated Cyclization

Amide-cyclized peptides **5–12** were synthesized through the incorporation of allyl-protected Asp or Glu and Mtt-protected Dap or Dab into the linear peptide chain. The allyl group was selectively removed through the treatment of the resin-bound peptide with Pd(PPh_3_)_4_ (25 mol %) and PhSiH_3_ (30 equiv) in anhydrous DCM, three times for 1 h, under a protective argon atmosphere. The resin was then washed with DCM (4 × 30 s), DMF (4 × 30 s), 0.1 M solution of Cupral in DMF (5 × 5 min), and finally with DMF (4 × 30 s). Mtt was removed from Dab/Dap by the treatment of the solid support attached peptide with a solution of 40% HFIP in DCM, containing 2.5% TIPS, four times for 20 min at rt. The Mtt removal was followed by washing the resin with DCM (4 × 30 s) and DMF (4 × 30 s).

Cyclization was achieved by coupling the side chains of Asp/Glu and Dab/Dap, where the resin-bound peptide was treated with PyBOP (2 equiv) and DIPEA (4 equiv) in DMF over 2 days at rt. Allyl-protected Asp in the case of peptides 5, 7, 9, and 11 proved to form a significant amount of aspartimide, and Dmb protection of the preceding residue was necessary: a Fmoc-deprotected, resin-bound DAD sequence was washed with a mixture of DMF/MeOH/AcOH (9:9:2) for 5 min, followed by washing with DMF/MeOH (1:1, 4 × 30 s). The resin was treated with 2,4-dimethoxybenzaldhyde (10 equiv) in DMF/MeOH (1:1) for 45 min at rt and washed with DMF/MeOH (1:1, 4 × 30 s). NaBH_3_CN (20 equiv) was added in DMF/MeOH/AcOH (9:9:2), the resin was shaken for 30 min at rt, and then washed with DMF/MeOH/AcOH (9:9:2), NMP, 5% DIPEA in NMP, NMP, and DMF (4×), affording the Dmb-protected DAD peptide intermediate on a solid support. Dmb-DAD gave a negative Kaiser test result, and the product could be observed by LC–MS after cleavage from the resin. Fmoc-Asp(OAll) (4 equiv) was coupled to this sequence using COMU (3.6 equiv), Oxyma Pure (4 equiv), and DIPEA (8 equiv) in DMF three times for 24 h at rt. The uncoupled Dmb-DAD sequence was capped by treatment with Ac_2_O (10 equiv) and DIPEA (12 equiv) in DMF over 30 min at rt.

#### RCM-Mediated Cyclization

The dry resin was swelled in argon-flushed DCE for 20 min. The Hoveyda–Grubbs 2nd generation catalyst (10 mol %) was added, and the mixture was flushed with argon and heated under MWI for 10 min at 120 °C. The mixture was flushed with argon and heated again under MWI for 10 min at 120 °C with a new portion of the catalyst (10 mol %) and washed with DCM (4×) and DMF (4×). Where applicable, the double bond, created in RCM, was reduced: the resin was washed with NMP (4×), and then TPSH (30 equiv) and DIPEA (46 equiv) were added to the resin suspended in NMP. The mixture was shaken for 24 h at 50 °C, washed with NMP (2×), and the process was repeated two more times with fresh portions of reagents. The progress of the double bond reduction was monitored by LC–MS. The resin was washed with NMP (2×), DMF (4×), DCM (4×), Et_2_O (2×), H_2_O (4×), and DMF (4×). When possible, the *cis* and *trans* isomers were separated.

### Protein Expression and Purification

The protein expression and purification were performed exactly as previously reported in the publication by Krzyzanowski et al., 2021.^5^

### Protein Labeling with Alexa 488

To a solution of the full-length pICln protein (ca. 1 mg/mL) in 0.2 M bicarbonate buffer with 1 mM TCEP at pH 8.3 was added 10 mM Alexa 488 NHE-ester in DMSO (8 equiv). The solution was kept on ice under dark conditions overnight. The protein was washed 8–10 times with a buffer intended for the fluorescence polarization assay (50 mM HEPES, 250 mM NaCl, 1 mM TCEP, 8.0 pH) using an Amicon Ultra-0.5 mL spin filter (Merck, Germany). Protein concentration was determined using the NanoDrop spectrophotometer.

### Fluorescence Polarization (FP)

#### Direct Binding Assay

The assay was performed in a buffer of 50 mM 4-(2-hydroxyethyl)-1-piperazineethanesulfonic acid (HEPES), 250 mM NaCl, 1 mM tris(2-carboxyethyl)phosphine (TCEP), and 0.01% (v/v) Tween 20, pH 8.0, in black, 384-well plates with a total volume of 10 μL per well. The analyzed FITC-labeled peptides were tested at a final concentration of 1 nM, and the unlabeled PRMT5-MEP50 protein complex was titrated as two-fold dilution series. The plates were incubated at room temperature for 1 to 2 h and analyzed on a plate reader using 485 nm excitation and 535 nm emission wavelength. The assay was performed in triplicate.

#### Competitive Binding Assay

The competitive binding assay was performed in a buffer of 50 mM HEPES, 250 mM NaCl, 1 mM TCEP, and 0.01% (v/v) Tween 20, 8.0 pH, in black, 384-well plates with a total volume of 10 μL per well. The FITC-labeled peptides or the Alexa 488-labeled pICln protein, at a final concentration of 1 nM, were mixed with PRMT5-MEP50 (at a final concentration of 194 nM when used with peptide **50**, 600 nM with compound **S1** [see Table S3] and **21**, and 27 nM in the case of Alexa 488-labeled pICln), and the appropriate nonlabeled peptide or protein was titrated as two-fold dilution series. The plates were incubated at room temperature for up to 1 h 45 min and analyzed on a plate reader using 485 nm excitation and 535 nm emission wavelength. The experiment was performed in triplicate.

### Stability Assay

To the whole cell lysate prepared from U2OS cells with the freeze–thaw method was added the appropriate peptide, resulting in a mixture of 0.5 mg/mL protein and 600 μM peptide in PBS, and incubated at 37 °C. The samples were taken at 0, 15, 30 min, 1, 2, 4 h, 1, 3, and 7-days time points and mixed with an equal volume of ice-cold ethylparaben solution in MeOH (0.1 mg/mL), used as the internal standard. The samples were kept on ice for 15 min and centrifuged at 16,873 × g at 4 °C for 5 min. The obtained supernatant was then analyzed by HPLC, the peptide and the internal standard peaks at 210 nm were integrated, and the ratio of the peptide peak surface area to the internal standard was calculated. The experiment was conducted in duplicate.

### Pull-Down

DBCO agarose beads (300 μL; Jena Bioscience, Germany) were washed three times with PBS (600 μL, pH 7.4). To the beads suspended in PBS (600 μL) was added a 10 mM solution of azide-labeled peptide in DMSO or pure DMSO. The obtained suspensions were incubated overnight at 4 °C. The beads were washed with PBS three times for 10 min. MCF7 cells were harvested and lysed using Triton X100 lysis buffer (NaCl 150 mM, HEPES 25 mM, TCEP 1 mM, 1% Triton x100, 1% NP40 alternative, protease, and phosphatase inhibitor, pH 7.4). Empty beads, beads bound to an active peptide (**55**), or beads bound to a scrambled peptide (**56**) were added to the lysate (6 mg/mL) and rotated at 4 °C overnight. Beads attached to the active peptides were also incubated in the lysate containing a nonimmobilized/free active macrocycle (**53** at 100 μM). Beads were collected by centrifugation (800*g* for 1 min) and washed three times for 10 min with PBS (500 μL). The beads were suspended in SDS-PAGE protein loading buffer (20 uL; 40 μM Tris, 8% v/v glycerol, 2% SDS, 80 μM dithioerythritol (DTE), 0.02% bromophenol blue, pH 6.8), heated with shaking for 10 min at 95 °C, and centrifuged at 350 rpm. The obtained solutions were analyzed by western blotting using 10% acrylamide gels, and PVDF membranes and the relevant proteins were fluorescently detected using antibodies against PRMT5 and MEP50 and the corresponding secondary IRDye antibodies.

### MTase-Glo Activity Assay

The methyltransferase activity of the expressed and purified PRMT5-MEP50 complex was tested in the presence of the active site inhibitor EPZ015666 and the adaptor site PPI inhibitor **30** using the MTase-Glo Methyltransferase assay kit from Promega (Promega Corporation).^31^ The assay was performed in 1× reaction buffer of 20 mM Tris, 50 mM NaCl, 1 mM EDTA, 3 mM MgCl_2_, 0.1 mg/mL BSA, and 1 mM DTT at pH = 8.0 in white, 384-well plates. A dilution series of EPZ015666 or peptide **30** was prepared in a mixture of 2 μM S-adenosylmethionine, 1 μM H4 histone tail peptide (see Figure S8), and 2× MTase-Glo reagent in the 1× reaction buffer (2.5 μL per well), and an equal volume of 200 nM PRMT5-MEP50 protein solution was added to give a total volume of 5 μL per well. The plate was incubated at 20–21 °C for 1 h. MTase-Glo detection solution (5 μL) was added, and the plate was incubated at 20–21 °C for 1 h, followed by a luminescence measurement at a plate reader. The experiment was performed in triplicate.

### Thermal Shift Assay (TSA)

Thermal Shift Assays of peptides on the TIM-MEP50 complex were carried out in a 96-well PCR plate (LightCycler 480 Multiwell Plates 96, white, 04729692001). The purified protein complex was appropriately diluted in a buffer containing 50 mM HEPES (pH = 8.0), 250 mM NaCl, and 1 mM TCEP. All assay experiments used 5.36 μg of protein per well and 140 nL 5000X Sypro Orange (Invitrogen) up to a total volume of 25 μL, with a resultant protein concentration of 3 μM and 5X SYPRO. Peptides were supplied at 10 mM concentration in DMSO. The PCR plates were sealed with an optical seal (LightCycler 480 Sealing Foil, 04729757001), shaken, and centrifuged after protein and compounds were added. Thermal scanning (25–95 °C at 1 °C/min) was performed using a real-time PCR setup (LightCycler 480 – Roche), and fluorescence intensity was measured after every temperature increment step. Analysis of the raw data was performed using internally developed software. Statistical validation of *T*_m_ shift relevance was performed using the Student’s *t* test (*n* = 6).

### Cell Culture

MCF7 and U2OS cells were cultured in DMEM medium, supplemented with 10% fetal bovine serum, 1% sodium pyruvate, and 1% nonessential amino acids at 37 °C. The MCF7 medium was further supplemented with 10 μg/mL insulin. Generation of the inducible Flp-In T-Rex 293 cell system expressing GFP, GFP-PRMT5, GFP-RioK1, and GFP-pICln was carried out according to the manufacturer′s instructions (Invitrogen, Thermo Fisher Scientific) and has been described previously.^29,30^ All Flp-In T-Rex 293 cells were cultured in DMEM (4.5 g/L d-glucose; Gibco, Thermo Fisher Scientific) supplemented with 10% (v/v) FCS (Biochrom, Merck), 100 U/mL penicillin, and 100 μg/mL streptomycin (Gibco, Thermo Fisher Scientific) under a 5% CO_2_ humidified atmosphere at 37 °C. For induction of GFP, GFP-PRMT5, GFP-RioK1, and GFP-pICln expression, Flp-In T-Rex 293 cell lines were stimulated with 0.1 μg/mL doxycycline (Clontech) for 18 h.

### GFP Immunopurification and Immunoblotting

#### Generation of S100 Extract

Harvested Flp-In T-Rex 293 cells were incubated with Roeder A buffer^32^ in three times sample weight for 10 min at room temperature, dounced 10 times, and adjusted to 150 mM NaCl. After centrifugation at 17,000 g for 30 min, the supernatants (S100 extracts) were used for immunopurification and treatment with inhibitors.

#### Inhibitor Treatment and Immunopurification

For the treatment, the S100 extracts of GFP, GFP-PRMT5, GFP-RioK1, and GFP-pICln were incubated with DMSO, active **53**, and scrambled **54**, used at a concentration of 50 μM. The incubation with the S100 extract was carried out for 1 h at room temperature. For GFP immunopurification, S100 extracts were incubated with GFP-Trap_A beads (ChromoTek) at 4 °C for 2 h with rotation. Purified proteins were washed 3 times with washing buffer (150 mM NaCl, 50 mM Tris/HCl pH 7.5, 1 mM EDTA, 1 mM EGTA, and 0.01% Igepal with protease inhibitors), eluted in sample buffer [375 mM Tris pH 7.5; 25.8% (w/v) glycerol; 12.3% (w/v) SDS; 0.06% (w/v) bromophenol blue; and 6% (v/v) β-mercaptoethanol; pH 6.8], and analyzed by immunoblotting.

#### Immunoblotting

Immunopurification samples were separated by Tris/Tricine or Tris/Glycine SDS gel electrophoresis^33^ and transferred to PVDF membranes (Immobilon-FL, Merck Millipore). The immunoblot analysis was performed using the indicated antibodies against RioK1, PRMT5, MEP50, pICln, and GFP, and the signals were detected with an Odyssey LI-COR Imaging System.

### Computational Modeling

Computational modeling was performed using the Maestro environment, version 12.3.013, and the Schrödinger suite, release 2020-1 (Schrödinger Inc.). Protein preparation was done with the Protein Preparation Wizard (Schrödinger), with the amino acid protonation states refined using PROPKA with pH set to 8.0.^34^ The macrocyclic compounds were built upon the co-crystal structure obtained for the RioK1-derived peptide sequence and TIM barrel of PRMT5. Using the Conformational Search tool (Schrödinger) with the OPLS_2005 force field and the water solvent, different conformations were generated for the cyclic core of the molecules with all of the remaining atoms fixed.^35^ Ligand refinement was performed with Glide v8.6 (Schrödinger), using first the standard precision, then followed by the extra precision ligand refinement.^36−38^ The obtained models were evaluated visually.

### Synthesis of BRD0639

The synthesis of the covalent inhibitor BRD0639 was based on the previously published protocols by McKinney and colleagues (Scheme S4).^16^

#### 2-Methyl-5-nitro-*N*-[2-(pyridin-2-yl)ethyl]benzene-1-sulfonamide (**57**)

A solution of 2-methyl-5-nitrobenzene-1-sulfonyl chloride (2.5 g, 10.61 mmol) and 2-pyridylethylamine (1.90 mL, 15.92 mmol) in THF (50 mL) was cooled to 0 °C, and TEA (4.44 mL, neat, 31.83 mmol) was added dropwise. The reaction mixture was then stirred at rt for 14 h, the solvent was evaporated in vacuo, and the crude was dissolved in EtOAc and washed with H_2_O. The aq phase was back-extracted with EtOAc (2×), and the combined organic layers were washed with sat. brine, dried (Na_2_SO_4_) and evaporated under reduced pressure, affording **57** as an off-white solid (3.283 g, 10.21 mmol, 96% yield): ^1^H NMR (400 MHz, CDCl_3_): δ [ppm] 8.81 (d, 1H), 8.51 (d, 1H), 8.26 (dd, 1H), 7.72 (t, 1H), 7.47 (d, 1H), 7.27 (t, 1H), 7.20 (d, 1H), 6.95 (t, 1H), 3.43 (q, 2H), 3.07 (t, 2H), 2.77 (s, 3H).

#### 5-Amino-2-methyl-*N*-[2-(pyridin-2-yl)ethyl]benzene-1-sulfonamide (**58**)

Compound **57** (1 g, 3.11 mmol) was dissolved in MeOH (35 mL), and Pd/C (25 mg, 10% w/w) was added. The mixture was briefly purged under reduced pressure and refilled with H_2_ three times. The reaction mixture was stirred at rt for 16 h under an H_2_ atmosphere (1 atm) and then filtered through Celite. The solvent was evaporated in vacuo, resulting in **58** as an off-white solid (896 mg, 3.08 mmol, 99% yield): LR-MS [*m*/*z*]: calculated [M + H]^+^ for C_14_H_18_N_3_O_2_S 292.1, found 291.8; ^1^H NMR (400 MHz, DMSO-*d*_6_): δ [ppm] 8.43 (m, 1H), 7.66 (tt, 1H), 7.47 (s, broad peak, 1H), 7.21–7.13 (m, 2H), 7.10 (d, 1H), 6.97 (d, 1H), 6.65 (dd, 1H), 5.28 (s, 2H), 3.11 (t, 2H), 2.82 (t, 2H), 2.31 (s, 3H).

#### (2S)-2-(4-Chloro-6-oxo-pyridazin-1-yl)propanoic Acid (**59**)

A mixture of 5-chloro-3(2H)-pyridazinone (300 mg, 2.30 mmol), methyl (2R)-2-hydroxypropanoate (435 μL, 4.60 mmol), and PPh_3_ (1.21 g, 4.60 mmol) was cooled to 0 °C, and DIAD (903 μL, neat, 4.60 mmol) was added. The reaction mixture was then stirred at rt for 2 h, and the solvent was removed under reduced pressure resulting in a yellow crude oil. Intermediate methyl(S)-2-(4-chloro-6-oxopyridazin-1(6H)-yl)propanoate was isolated through flash chromatography (silica gel, 20:1 → 9:1, petroleum ether/EtOAc) as a yellow oil, which was suspended in aq HCl (5% w/v, 4 mL) and refluxed for 12 h. The reaction mixture was cooled to room temperature, and the solvent was evaporated in vacuo affording a brown oil. The crude was purified by flash chromatography (silica gel, 5 → 8% MeOH in DCM with the addition of 0.5% (v/v) AcOH). The combined fractions containing the product were evaporated under reduced pressure with the addition of toluene, resulting in the title molecule **59** as a white solid (290 mg, 1.43 mmol, 62% yield): ^1^H NMR (400 MHz, DMSO-*d*_6_): δ [ppm] 8.13 (d, 1H), 7.31 (d, 1H), 5.33 (q, 1H), 1.52 (d, 3H).

#### (2S)-2-(4-Chloro-6-oxo-pyridazin-1-yl)-N-[4-methyl-3-[2-(2-pyridyl)ethylsulfamoyl]phenyl]propanamide (**BRD0639**)

To a mixture of **58** (71.9 mg, 0.247 mmol) and **59** (50 mg, 0.247 mmol) in THF (1 mL) was added DIPEA (172 μL, neat, 0.987 mmol) dropwise over 5 min at rt. The reaction mixture was stirred at rt for 1 h, the solvent was removed in vacuo, and the resulting crude mixture was purified by preparative HPLC (C18, A: 10 mM aq NH_4_HCO_3_, B: ACN; 15 → 75% B over 60 min, product eluted at ca. 40% B), affording the covalent PRMT5 PPI inhibitor BRD0639 as a white solid (67 mg, 0.141 mmol, 57% yield): HRMS [*m*/*z*]: calculated [M + H]^+^ for C_21_H_23_ClN_5_O_4_S 476.11538, found 476.11611; ^1^H NMR (400 MHz, DMSO-*d*_6_): δ [ppm] 10.42 (s, 1H), 8.47 (d, 1H), 8.16 (d, 1H), 8.08 (d, 1H), 7.81–7.67 (m, 3H), 7.33–7.22 (m, 4H), 5.39 (q, 1H), 3.17 (q, 2H), 2.87 (t, 2H), 2.42 (s, 3H), 1.60 (d, 3H). The spectral data of BRD0639 were found to be in accordance with the previously reported results.^16^

## Supporting Information Available

Peptide sequences and HRMS data (Table S1, S3, S4); FP assay results (Table S2, Figures S1–S5 and S9–S13); synthetic schemes (Schemes S1–S4); stability in cell lysate data (Figures S6 and S7); MTase-Glo assay results (Figure S8); structures of **55** and **56** (Figure S14); results of the pull-down assay (Figure S17); results of IP (Figure S17); HPLC traces for peptides (PDF)
Computer models of macrocycles (ZIP)
Molecular formula strings (CSV)

## Abbreviations Used

Dab: diaminobutyric acid
Dap: diaminopropionic acid
DBCO: dibenzocyclooctyne
DIPEA: *N*,*N*-diisopropylethylamine
Dmb: dimethoxybenzyl group
DTE: dithioerythritol
FITC: fluorescein isothiocyanate
FP: fluorescence polarization
HEPES: 4-(2-hydroxyethyl)-1-piperazineethanesulfonic acid
HFIP: hexafluoroisopropanol
IP: immunoprecipitation
PAPII: PRMT5–adaptor protein interaction inhibitor
PBM: PRMT5 binding motif
PPI: protein–protein interaction
PRMT5: protein arginine methyltransferase 5
SPPS: solid phase peptide synthesis
TCEP: tris(2-carboxyethyl)phosphine
TPSH: 2,4,6-triisopropylbenzenesulfonyl hydrazide
TSA: thermal shift assay
