# Supplementary material for: Development of Macrocyclic PRMT5–Adaptor Protein Interaction Inhibitors
Source: J Med Chem. 2022 Nov 15;65(22):15300–11. doi: 10.1021/acs.jmedchem.2c01273 (PMC9706563; doi:10.1021/acs.jmedchem.2c01273)

Supporting Information

Development of Macrocyclic PRMT5 Adaptor Protein Interaction Inhibitors

Adrian Krzyzanowski,[a, b] Lea Marie Esser,[c] Anthony Willaume,[d] Renaud Prudent,[d] Christoph Peter,[c] Peter ‘t Hart*[e] and Herbert Waldmann*[a, b]

[a] A. Krzyzanowski, Prof. Dr. H. Waldmann
Department of Chemical Biology
Max Planck Institute of Molecular Physiology
Otto-Hahn-Straße 11, 44227 Dortmund, Germany

[b] A. Krzyzanowski, Prof. Dr. H. Waldmann
Faculty of Chemistry, Chemical Biology
Technical University Dortmund
Otto-Hahn-Straße 6, 44221 Dortmund, Germany

[c] L.M. Esser, Dr. C. Peter
Institute of Molecular Medicine I
Medical Faculty and University Hospital, Heinrich Heine University Düsseldorf
Universitätsstraße 1, 40225 Düsseldorf, Germany

[d] Dr. A. Willaume, Dr. R. Prudent
Edelris
Bioserra 1, 60 Av. Rockefeller, 69008 Lyon, France

[e] Dr. P. ‘t Hart
Chemical Genomics Centre of the Max Planck Society
Max Planck Institute of Molecular Physiology
Otto-Hahn-Straße 11, 44227 Dortmund, Germany

* Corresponding Authors:
P.t.H. Peter.t-Hart@mpi-dortmund.mpg.de
H.W. Herbert.Waldmann@mpi-dortmund.mpg.de

Table of Contents

Supporting Tables S3
Supporting Synthetic Schemes S6
Supporting Figures S8
HPLC Traces....................S18

Supporting Tables

Table S1. Sequences and HRMS of linear peptides with amino acid modifications.

Name	Sequence	HRMS	
		m/z calculated	m/z found
**S2**	Ac-**Tyr**-PGQFDDADK(Fitc)-NH_2	1585.55764 $[M+H]^+$	1585.56081
**S3**	Ac-**Phe**-PGQFDDADK(Fitc)-NH_2	1569.56272 $[M+H]^+$	1569.56604
**S4**	Ac-**hPhe**-PGQFDDADK(Fitc)-NH_2	1583.57837 $[M+H]^+$	1583.58125
**S5**	Ac-**Trp**-PGQFDDADK(Fitc)-NH_2	1608.57362 $[M+H]^+$	1608.57625
**S6**	Ac-**Asp**-PGQFDDADK(Fitc)-NH_2	1537.52125 $[M+H]^+$	1537.52441
**S7**	Ac-**Glu**-PGQFDDADK(Fitc)-NH_2	1551.53690 $[M+H]^+$	1551.54015
**S8**	Ac-VPG-**Asn**-FDDADK(Fitc)-NH_2	1507.54707 $[M+H]^+$	1507.55099
**S9**	Ac-VPG-**Dab**-FDDADK(Fitc)-NH_2	1493.56781 $[M+H]^+$	1493.56975
**S10**	Ac-VPG-**Dab(Alloc)**-FDDADK(Fitc)-NH_2	1577.58894 $[M+H]^+$	1577.59177
**S11**	Ac-VPG-**Cit**-FDDADK(Fitc)-NH_2	1550.58927 $[M+H]^+$	1550.59317
**32**	Ac-VPGQ-**Phe(3-F)**-DDADK(Fitc)-NH_2	1539.55330 $[M+H]^+$	1539.55557
**34**	Ac-VPGQ-**Bip**-DDADK(Fitc)-NH_2	1597.59402 $[M+H]^+$	1597.59731
**35**	Ac-VPGQ-**Phe(3,4-F_2)**-DDADK(Fitc)-NH_2	1557.54388 $[M+H]^+$	1557.54775
**36**	Ac-VPGQ-**Phe(4-F)**-DDADK(Fitc)-NH_2	1539.55330 $[M+H]^+$	1539.55660
**37**	Ac-VPGQ-**Phe(4-Br)**-DDADK(Fitc)-NH_2	1599.47323 $[M+H]^+$	1599.47609
**38**	Ac-VPGQ-**Phe(4-I)**-DDADK(Fitc)-NH_2	1647.45936 $[M+H]^+$	1647.46158
**39**	Ac-VPGQ-**Phe(4-Cl)**-DDADK(Fitc)-NH_2	1555.52375 $[M+H]^+$	1555.52694
**41**	Ac-VPGQ-**Phe(4-NO_2)**-DDADK(Fitc)-NH_2	1566.54780 $[M+H]^+$	1566.55130
**S12**	Ac-VPGQ-**Phe(2-F)**-DDADK(Fitc)-NH_2	1539.55330 $[M+H]^+$	1539.55666
**S13**	Ac-VPGQ-**Phe(F_5)**-DDADK(Fitc)-NH_2	1611.51561 $[M+H]^+$	1611.51901
**S14**	Ac-VPGQ-**Phe(2-I)**-DDADK(Fitc)-NH_2	1647.45936 $[M+H]^+$	1647.46197
**S15**	Ac-VPGQ-**Phe(3-Cl)**-DDADK(Fitc)-NH_2	1555.52375 $[M+H]^+$	1555.52705
**S16**	Ac-VPGQ-**Phe(3-CF_3)**-DDADK(Fitc)-NH_2	1589.55011 $[M+H]^+$	1589.55440
**S17**	Ac-VPGQ-**2-Pal**-DDADK(Fitc)-NH_2	1522.55797 $[M+H]^+$	1522.56122
**S18**	Ac-VPGQ-**Tyr**-DDADK(Fitc)-NH_2	1537.55764 $[M+H]^+$	1537.56120
**S19**	Ac-VPGQ-**Phe(4-COOH)**-DDADK(Fitc)-NH_2	1565.55255 $[M+H]^+$	1565.55556
**S20**	Ac-VPGQ-**Phe(4-guanidino)**-DDADK(Fitc)-NH_2	1578.59542 $[M+H]^+$	1578.59706
**S21**	Ac-VPGQ-**hPhe**-DDADK(Fitc)-NH_2	1535.57837 $[M+H]^+$	1535.58161
**S22**	Ac-VPGQ-**3-(2-biphenylyl)-Ala**-DDADK(Fitc)-NH_2	1597.59402 $[M+H]^+$	1597.59787
**S23**	Ac-VPGQ-**Bpa**-DDADK(Fitc)-NH_2	1625.58894 $[M+H]^+$	1625.59170
**S24**	Ac-VPGQ-**2-Nal**-DDADK(Fitc)-NH_2	1571.57837 $[M+H]^+$	1571.58183
**S25**	Ac-VPGQ-**1-Nal**-DDADK(Fitc)-NH_2	1571.57837 $[M+H]^+$	1571.58167
**S26**	Ac-VPGQFD-**Gla**-ADK(Fitc)-NH_2	1579.56820 $[M+H]^+$	1579.57063
**S27**	Ac-VPGQFDD-**Abu**-DK(Fitc)-NH_2	1535.57837 $[M+H]^+$	1535.58199
**S28**	Ac-VPGQFDD-**Nva**-DK(Fitc)-NH_2	1549.59402 $[M+H]^+$	1549.59737
**S29**	Ac-VPGQFDD-**Nle**-DK(Fitc)-NH_2	1563.60967 $[M+H]^+$	1563.61310
**S30**	Ac-VPGQFDD-**Cha**-DK(Fitc)-NH_2	1603.64097 $[M+H]^+$	1603.64485
**S31**	Ac-VPGQFDD-**Ser**-DK(Fitc)-NH_2	1537.55764 $[M+H]^+$	1537.56164
**33**	Ac-VPGQFDDA-**Glu**-K(Fitc)-NH_2	1535.57837 $[M+H]^+$	1535.58145
**40**	Ac-VPGQFDDA-**Gla**-K(Fitc)-NH_2	1579.56820 $[M+H]^+$	1579.57062
**S32**	Ac-VPGQFDDA-**Tyr**-K(Fitc)-NH_2	1569.59911 $[M+H]^+$	1569.60176
**S33**	Ac-VPGQFDDA-**Trp**-K(Fitc)-NH_2	1592.61509 $[M+H]^+$	1592.61751
**S34**	Ac-VPGQFDDA-**hPhe**-K(Fitc)-NH_2	1567.61984 $[M+H]^+$	1567.62296
**42**	Ac-VPGQ-**Phe(4-Cl)**-DDA-**Gla**-K(Fitc)-NH_2	1613.52923 $[M+H]^+$	1613.53080
**43**	Ac-VPGQ-**Phe(4-NO_2)**-DDA-**Gla**-K(Fitc)-NH_2	1624.55328 $[M+H]^+$	1624.55454

Table S2. Direct binding FP results for *N*-methylated linear peptides **44**-**49**.

Peptide	Sequence	K_D (µM)
**44**	Ac-VPGQFDDA-**(*N*-Me)D**-K(Fitc)-NH_2	0.6 ± 0.1
**45**	Ac-VPGQFDD-**(*N*-Me)A**-DK(Fitc)-NH_2	>2
**46**	Ac-VPGQFD-**(*N*-Me)D**-ADK(Fitc)-NH_2	>2
**47**	Ac-VPGQ-**(*N*-Me)F**-DDADK(Fitc)-NH_2	>2
**48**	Ac-VP-**(*N*-Me)G**-QFDDADK(Fitc)-NH_2	>2
**49**	Ac-**(*N*-Me)V**-PGQFDDADK(Fitc)-NH_2	>2

Table S3. Sequences and HRMS data of linear RioK1-derived peptides.

Name	Sequence	HRMS	
		m/z calculated	m/z found
**1**	Ac-SRVVPaQFDDAD-NH_2	1360.64917 $[M+H]^+$	1360.65083
**2**	Ac-SRVVPGQFaDAD-NH_2	1302.64369 $[M+H]^+$	1302.64518
**3**	Ac-SRVVPaQFaDAD-NH_2	1316.65934 $[M+H]^+$	1316.66100
**4**	Ac-SRVVPGQFDDAD-NH_2	1346.63352 $[M+H]^+$	1346.63623
**28**	Fitc-O2Oc-VPGQFDDAD-NH_2	1496.53109 $[M+H]^+$	1496.53270
**29**	Ac-VPGQFDDADK(Fitc)-NH_2	1521.56272 $[M+H]^+$	1521.56580
**31**	Ac-VPGQFDDAD-NH_2	1004.43197 $[M+H]^+$	1004.42952
**44**	Ac-VPGQFDDA-**(*N*-Me)D**-K(Fitc)-NH_2	1535.57837 $[M+H]^+$	1535.58195
**45**	Ac-VPGQFDD-**(*N*-Me)A**-DK(Fitc)-NH_2	1535.57837 $[M+H]^+$	1535.58169
**46**	Ac-VPGQFD-**(*N*-Me)D**-ADK(Fitc)-NH_2	1535.57837 $[M+H]^+$	1535.58178
**47**	Ac-VPGQ-**(*N*-Me)F**-DDADK(Fitc)-NH_2	1535.57837 $[M+H]^+$	1535.58196
**48**	Ac-VP-**(*N*-Me)G**-QFDDADK(Fitc)-NH_2	1535.57837 $[M+H]^+$	1535.58188
**49**	Ac-**(*N*-Me)V**-PGQFDDADK(Fitc)-NH_2	1535.57837 $[M+H]^+$	1535.58070
**S1**	Fitc-O2Oc-SRVVPGQFDDADSSD-NH_2	1064.41546 $[M+2H]^{2+}$	1064.41816
**H4 peptide**	Ac-SGRGKGGKGLGKGGAKRHRKV-NH2	1066.64103 $[M+2H]^{2+}$	1066.64198

Table S4. HRMS of cyclic peptides.

Name	HRMS	
	m/z calculated	m/z found
**5**	925.37795 $[M+2H]^{2+}$	925.37895
**6**	932.38578 $[M+2H]^{2+}$	932.38677
**7**	932.38578 $[M+2H]^{2+}$	932.38673
**8**	939.39360 $[M+2H]^{2+}$	939.39476
**9**	925.37795 $[M+2H]^{2+}$	925.37888
**10**	932.38578 $[M+2H]^{2+}$	932.38677
**11**	932.38578 $[M+2H]^{2+}$	932.38673
**12**	939.39360 $[M+2H]^{2+}$	939.39480
**13**	916.88287 $[M+2H]^{2+}$	916.88398
**14**	917.89069 $[M+2H]^{2+}$	917.89179
**15 isomer 1**	916.88287 $[M+2H]^{2+}$	916.88396
**15 isomer 2**	916.88287 $[M+2H]^{2+}$	916.88397
**16**	917.89069 $[M+2H]^{2+}$	917.89172
**17**	758.29791 $[M+2H]^{2+}$	758.29659
**18 isomer 1**	765.30573 $[M+2H]^{2+}$	765.30446
**18 isomer 2**	765.30573 $[M+2H]^{2+}$	765.30464
**19**	765.30573 $[M+2H]^{2+}$	765.30440
**20**	772.31356 $[M+2H]^{2+}$	772.31227
**21**	766.31356 $[M+2H]^{2+}$	766.31240
**22**	773.32138 $[M+2H]^{2+}$	773.32047
**23**	765.30573 $[M+2H]^{2+}$	765.30441
**24**	765.30573 $[M+2H]^{2+}$	765.30475
**25**	772.31356 $[M+2H]^{2+}$	772.31239
**26**	766.31356 $[M+2H]^{2+}$	766.31275
**27**	773.32138 $[M+2H]^{2+}$	773.32047
**30**	1014.48909 $[M+H]^{+}$	1014.48752
**50**	1634.61040 $[M+H]^{+}$	1634.61054
**51**	1438.48922 $[M+H]^{+}$	1438.48948
**52**	1634.61040 $[M+H]^{+}$	1634.61064
**53**	1117.47964 $[M+H]^{+}$	1117.48050
**54**	1117.47964 $[M+H]^{+}$	1117.48031
**55**	1460.66521 $[M+H]^{+}$	1460.66729
**56**	1460.66521 $[M+H]^{+}$	1460.66757

Supporting Synthetic Schemes

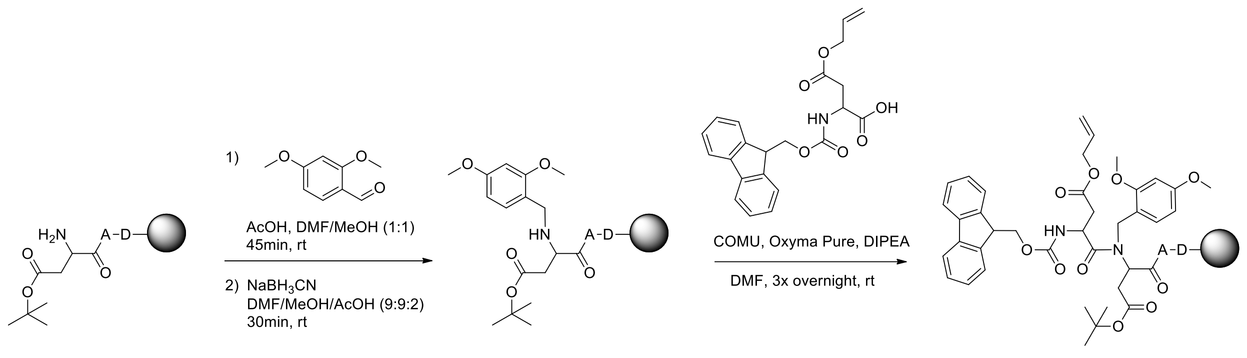

Scheme S1. On-resin protection with Dmb and coupling of the resulting Dmb-DAD sequence to Fmoc-Asp(OAll), en route to peptides **5**, **7**, **9** and **11**.

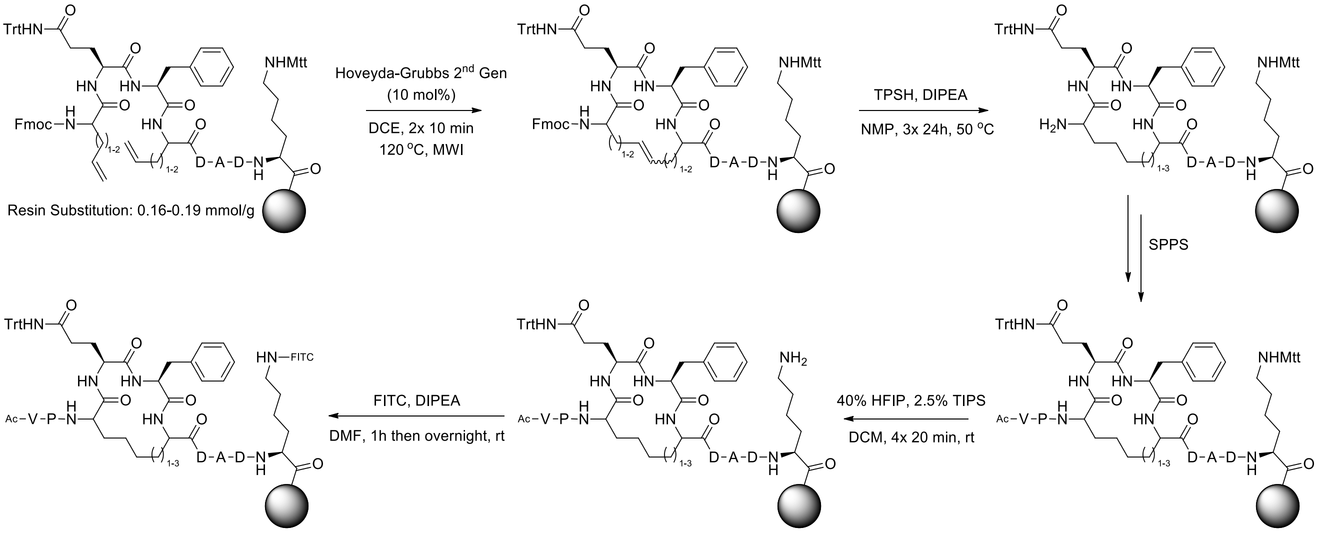

Scheme S2. Cyclisation through RCM and reduction of the resulting double bond, followed by Mtt removal and labelling with FITC for C-terminally labelled macrocycles. For peptides with a double bond the TPSH reduction step was omitted.

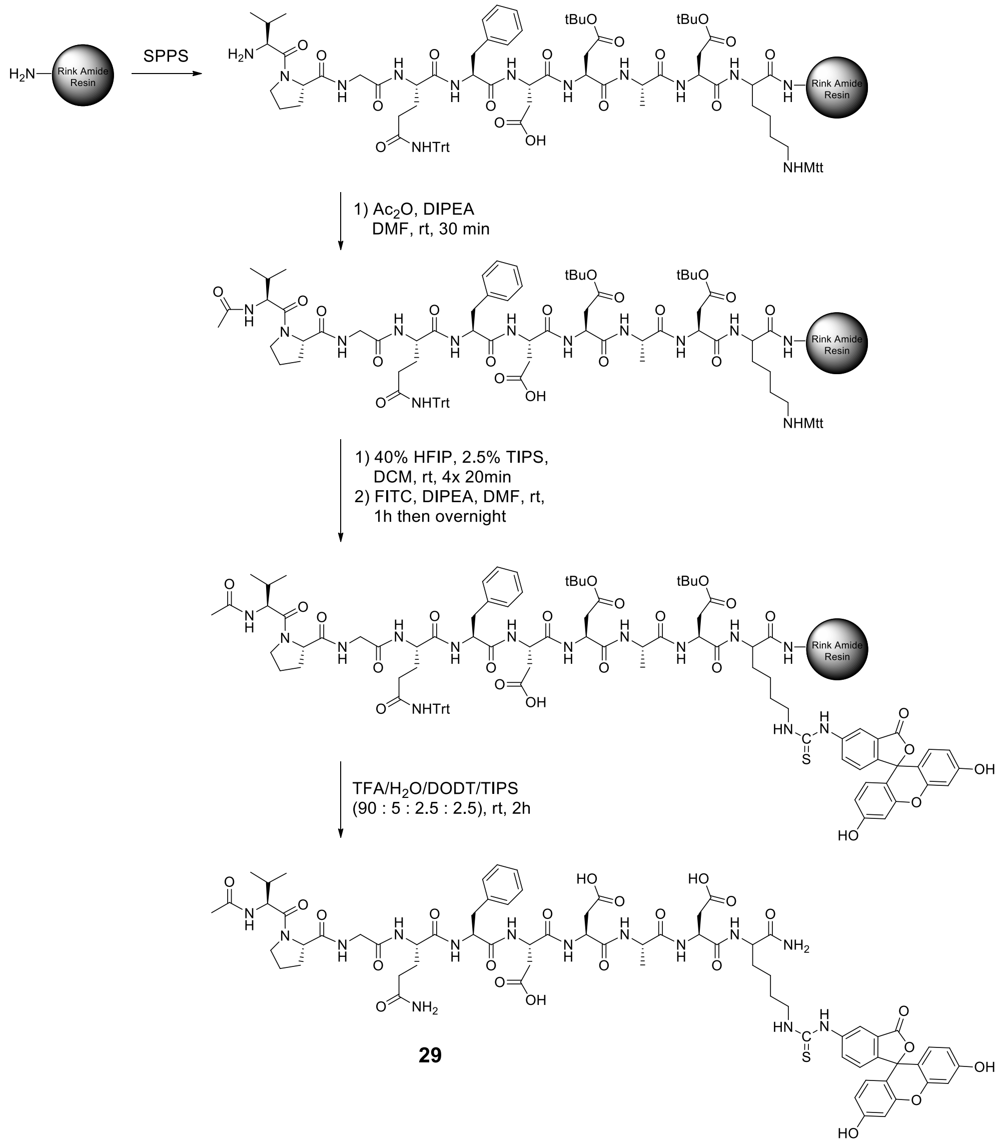

Scheme S3. An exemplary synthetic scheme leading to N-terminally acetylated and C-terminally FITC-labelled linear peptide **29**.

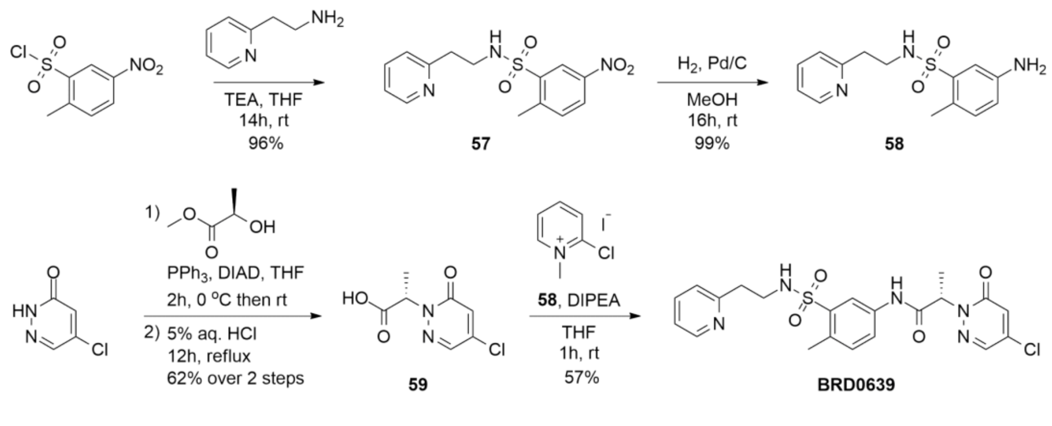

Scheme S4. Synthesis of the covalent PRMT5 PPI inhibitor BRD0639.

Supporting Figures

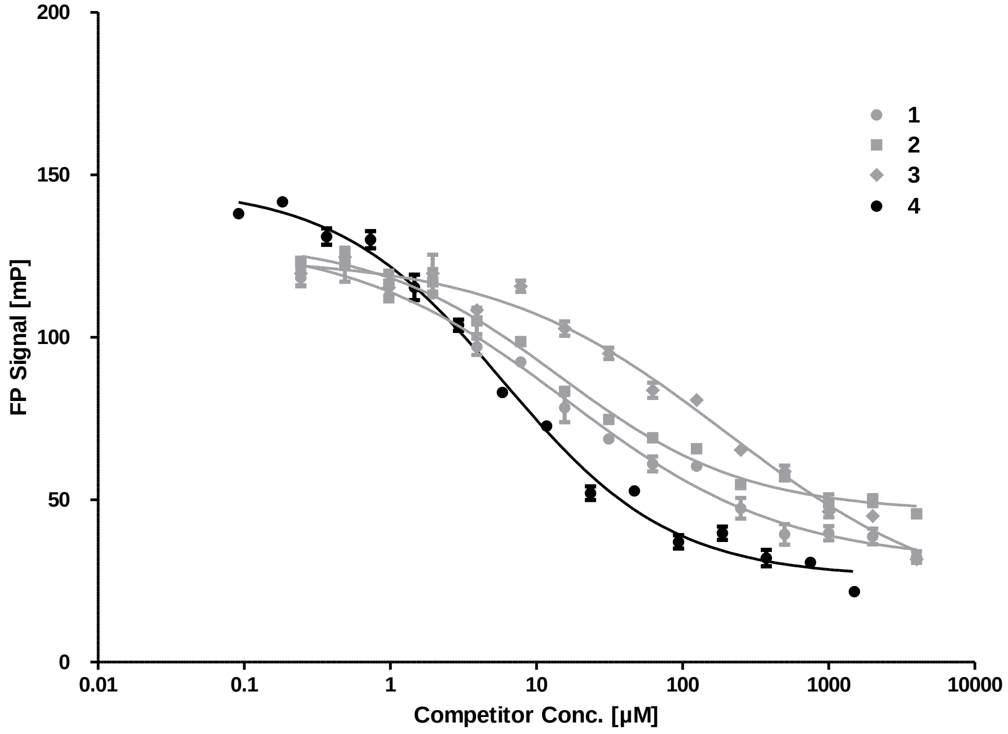

Figure S1. Competitive binding FP with PRMT5-MEP50 and peptide **S1** (Table S1) used as a fluorescent tracer, for compounds **1-4**.

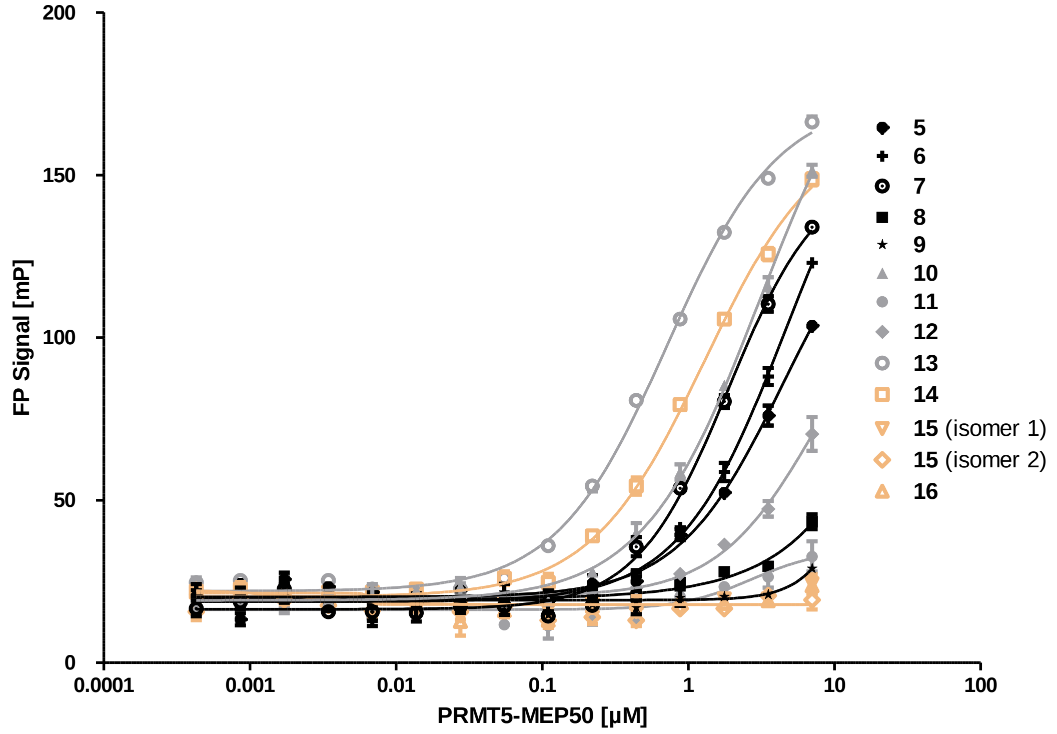

Figure S2. Direct binding FP with PRMT5-MEP50 for compounds **5-16**.

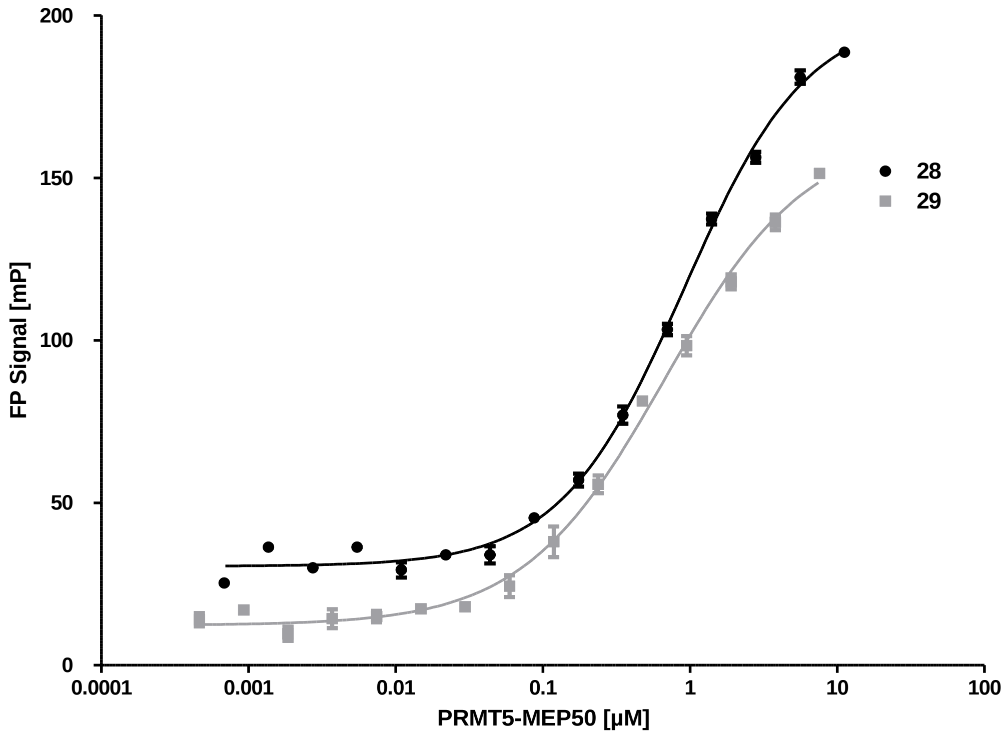

Figure S3. Direct binding FP with PRMT5-MEP50 for compound **28** and **29**.

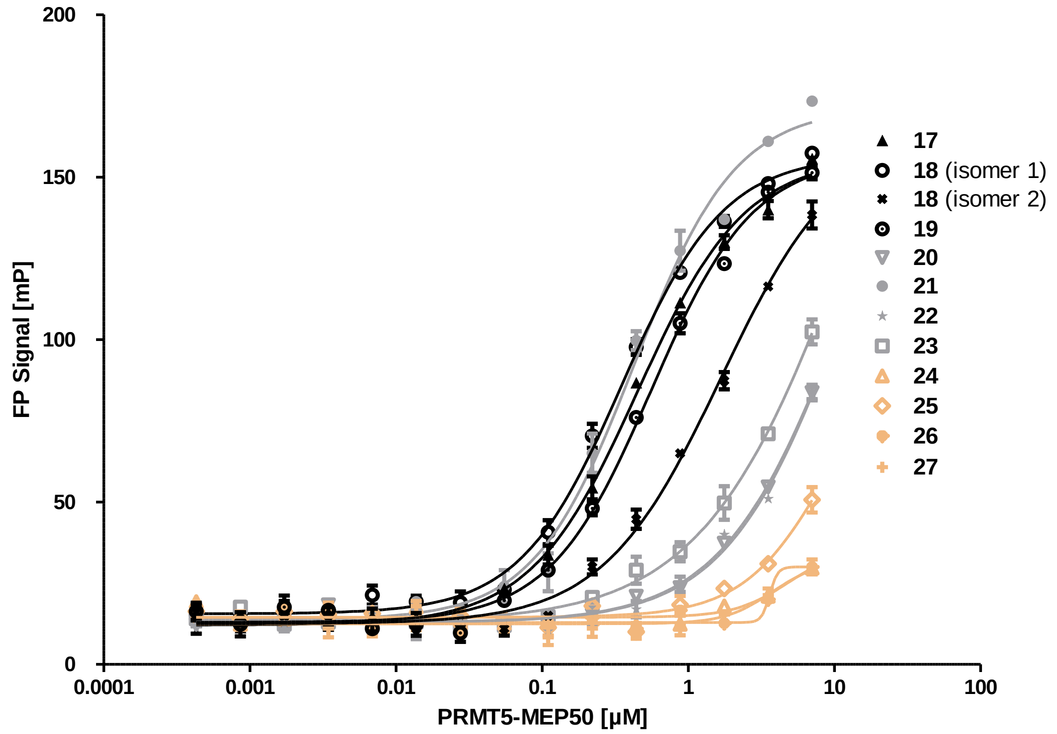

Figure S4. Direct binding FP with PRMT5-MEP50 for compounds **17-27**.

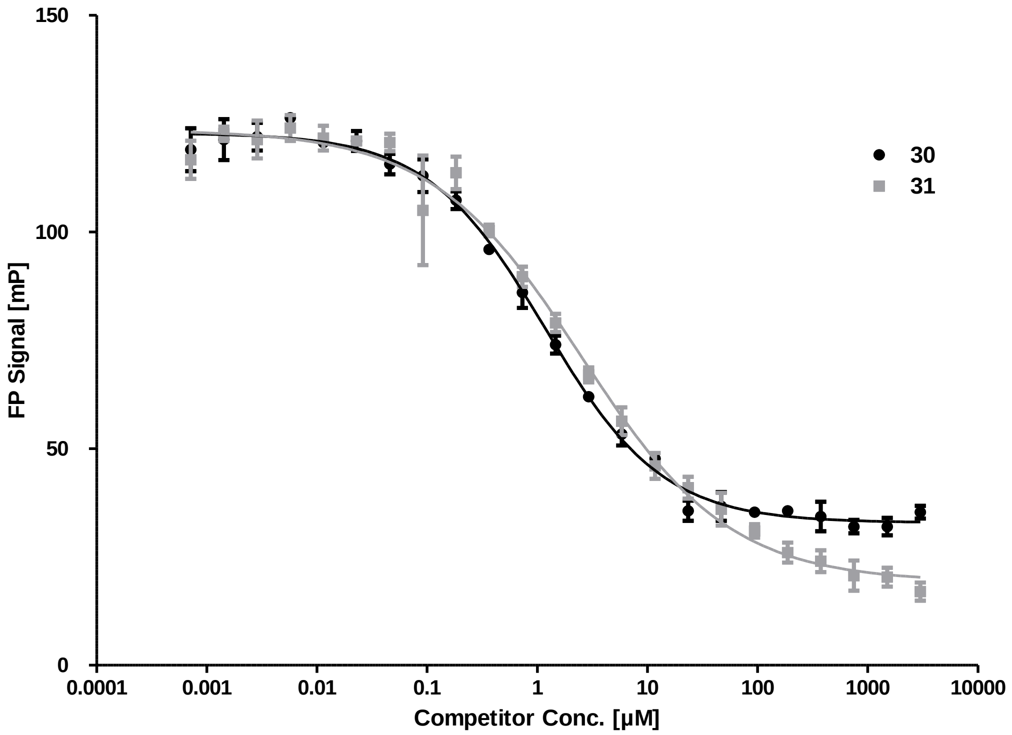

Figure S5. Competitive binding FP with PRMT5-MEP50 and fluorescent **21** used as a tracer, for compounds **30** and **31**.

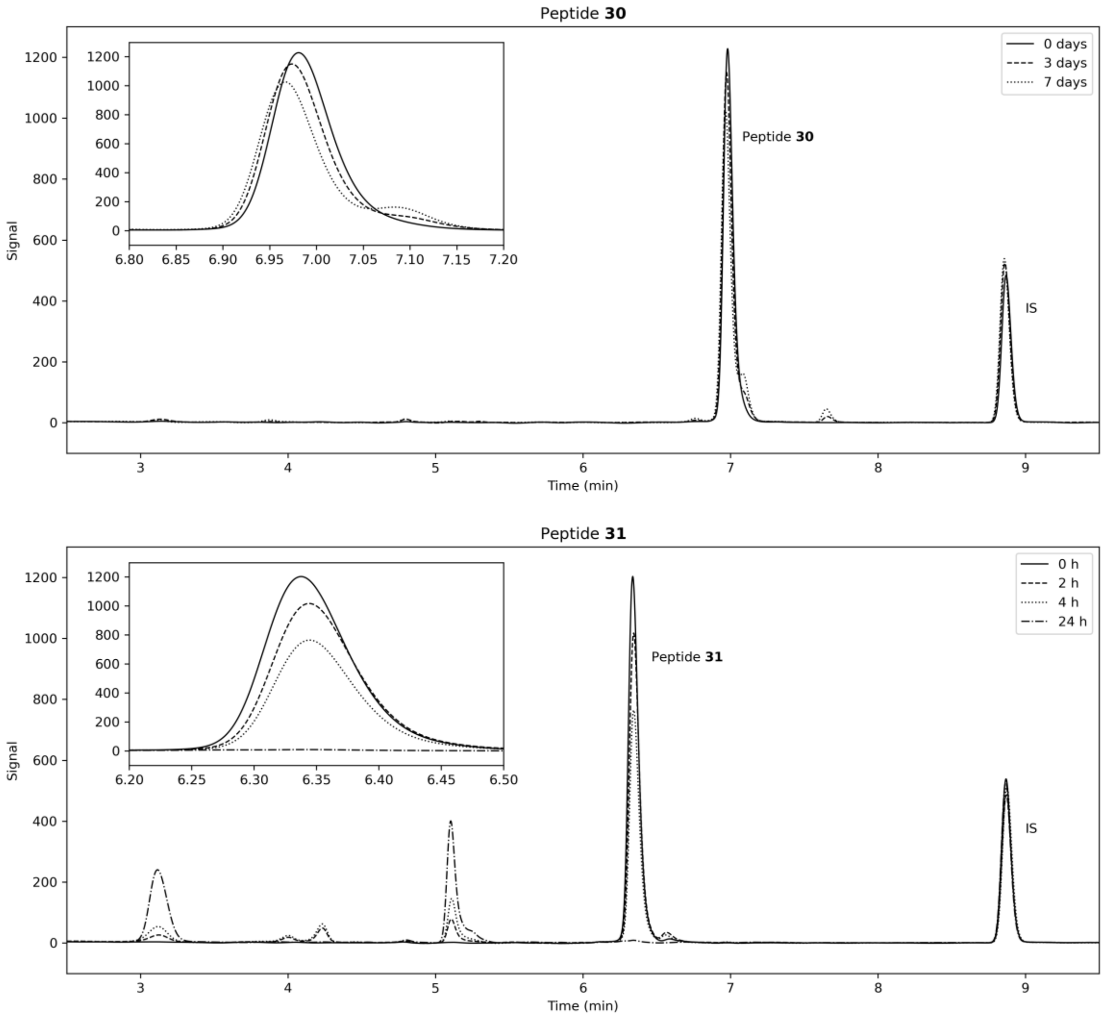

Figure S6. HPLC analysis of cyclic **30** and linear **31** after incubation in the U2OS cell lysate. Presented timepoints: between 0-7 days for **30** and between 0-24 h for **31**. IS = Internal Standard.

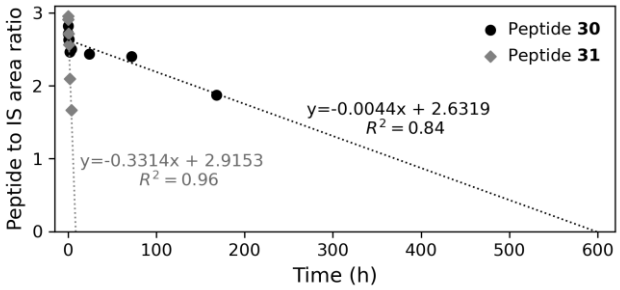

Figure S7. Linear regression models based on the obtained stability data for **30** and **31**. T½ = 299 h or 12.5 days for **30** and t½ = 4.4 h for **31** (n=2).

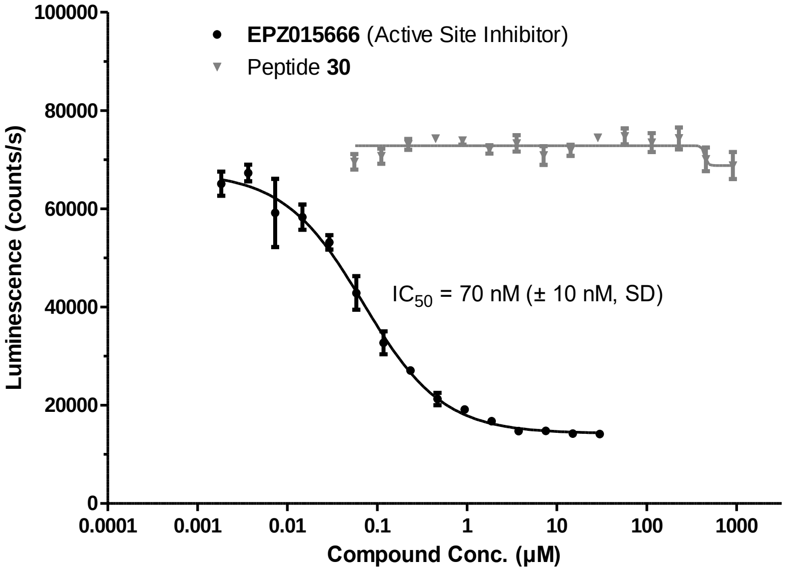

Figure S8. Mtase-Glo^{TM} Methyltransfarase Assay performed on the isolated PRMT-MEP50 complex with **30** and the reference active-site methyltransferase inhibitor **EPZ015666**. Compound **EPZ015666** is able to inhibit the direct methylation of the H4 histone tail peptide by PRMT5, whereas **30** has no effect (n=3).

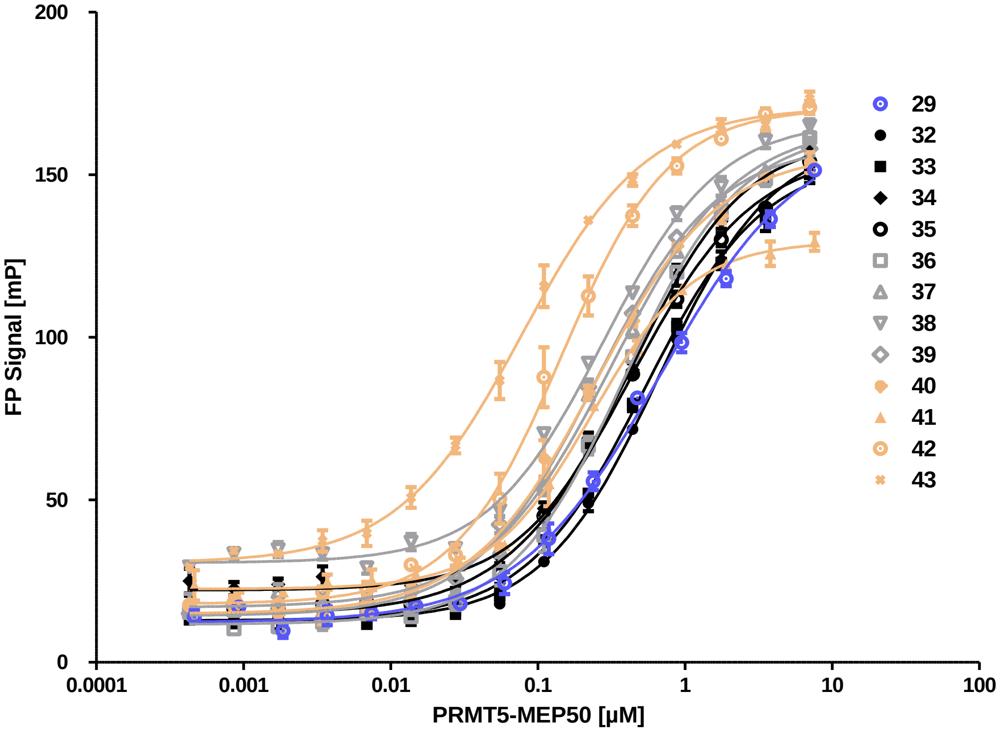

Figure S9. Direct binding FP with PRMT5-MEP50 for compounds **29** and **32-43**.

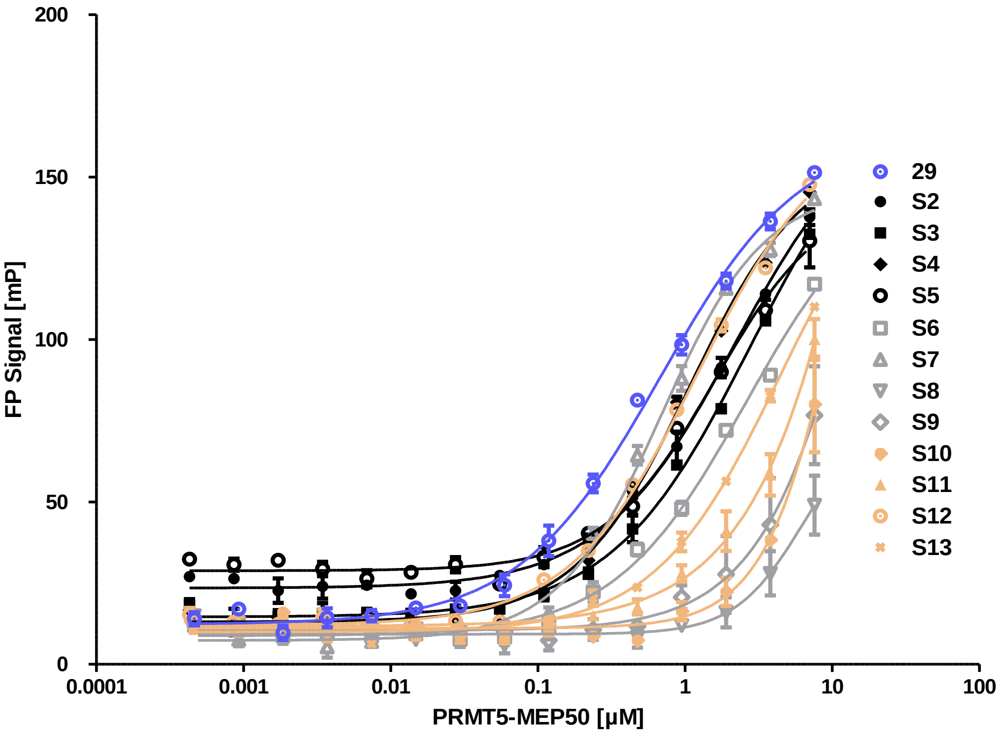

Figure S10. Direct binding FP with PRMT5-MEP50 for compounds **29** and **S3-S14**.

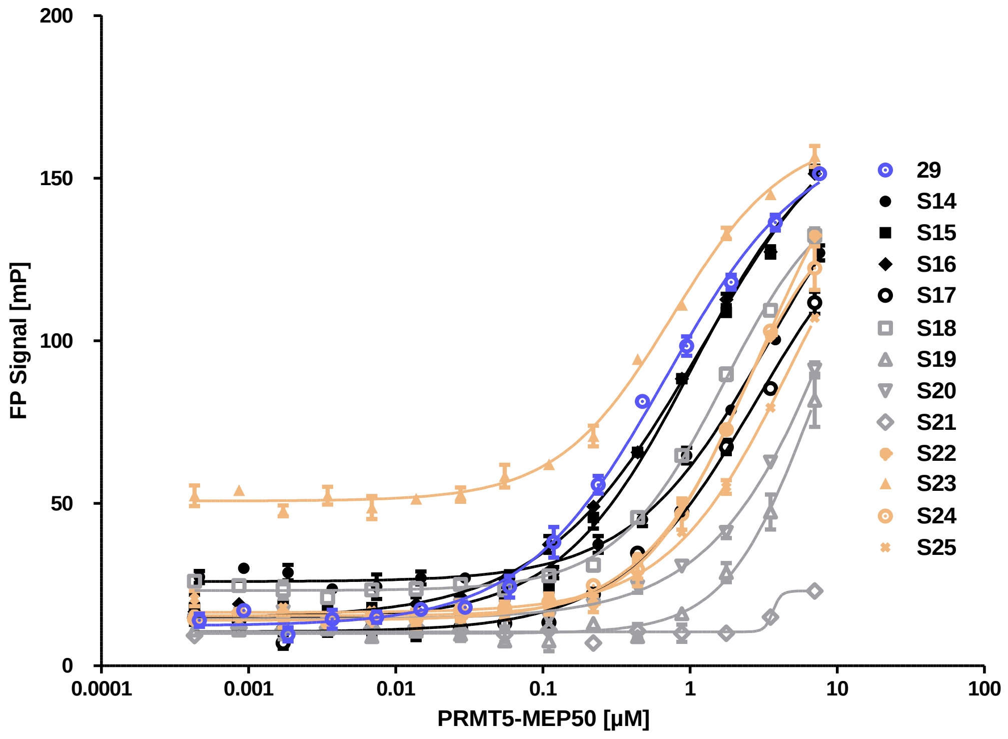

Figure S11. Direct binding FP with PRMT5-MEP50 for compounds **29** and **S14-S25**.

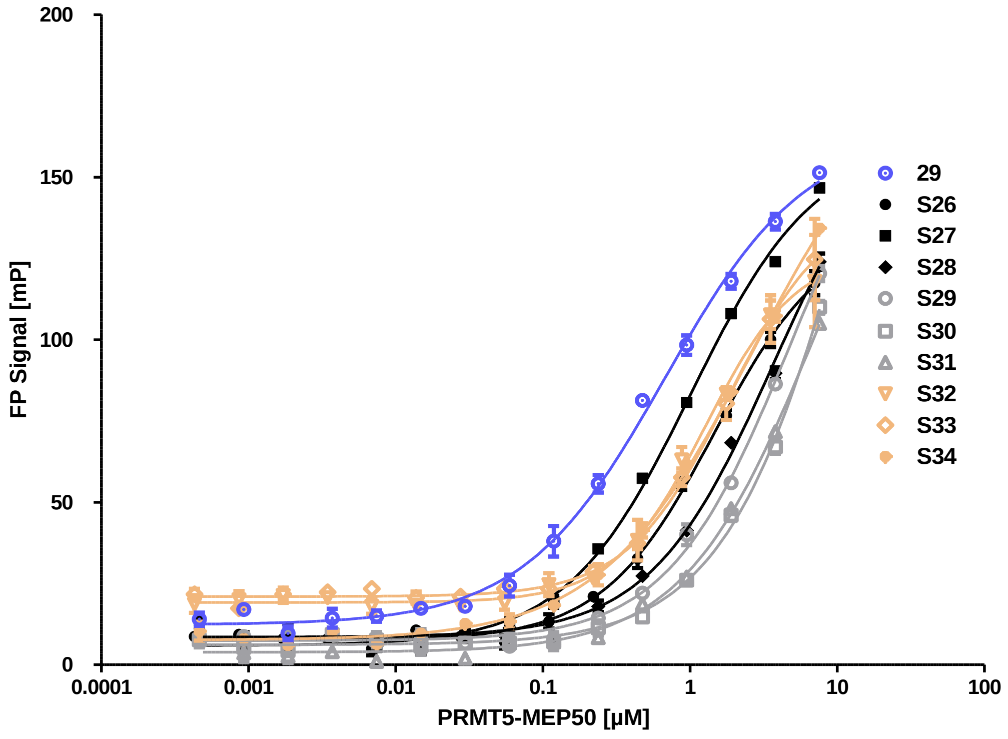

Figure S12. Direct binding FP with PRMT5-MEP50 for compounds **29** and **S26-S34**.

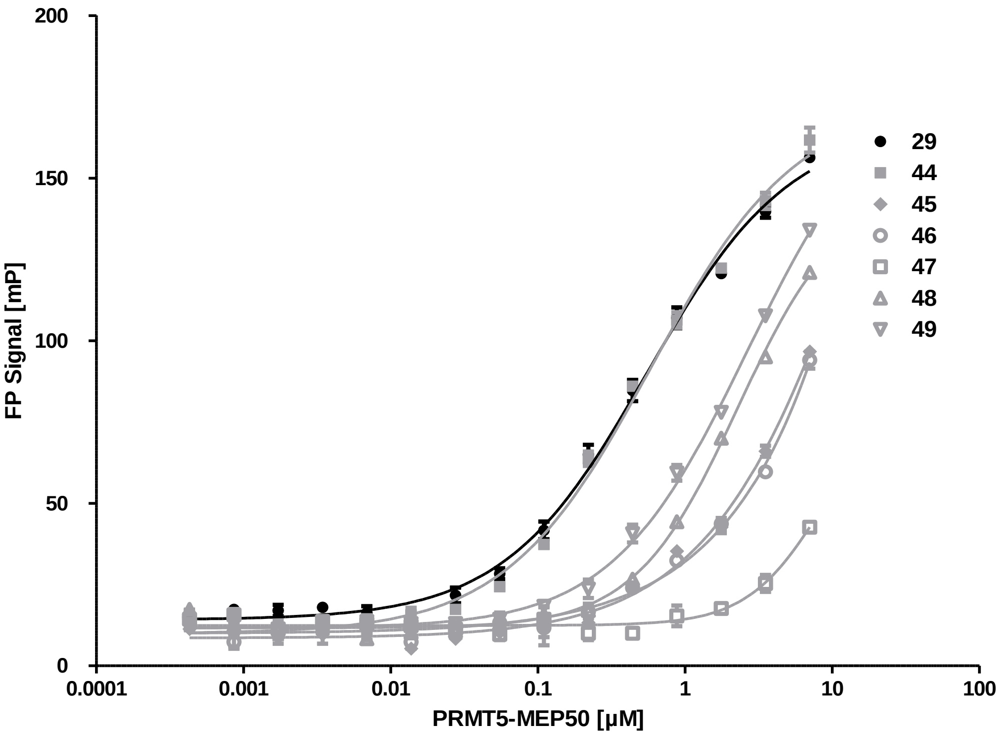

Figure S13. Direct binding FP with PRMT5-MEP50 for compounds **29** and **44-49**.

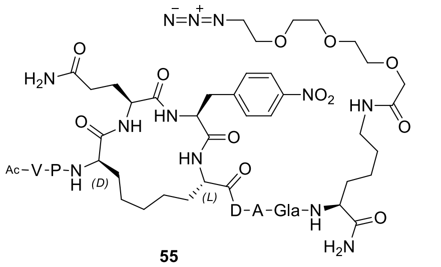

55

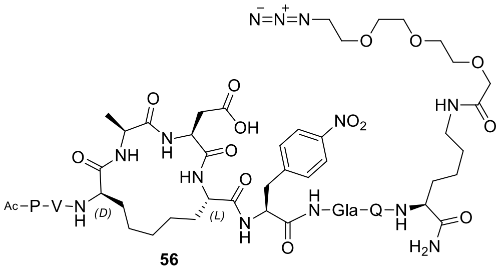

56

Figure S14. Structures of cyclic peptides **55** and **56**, equipped with azide group.

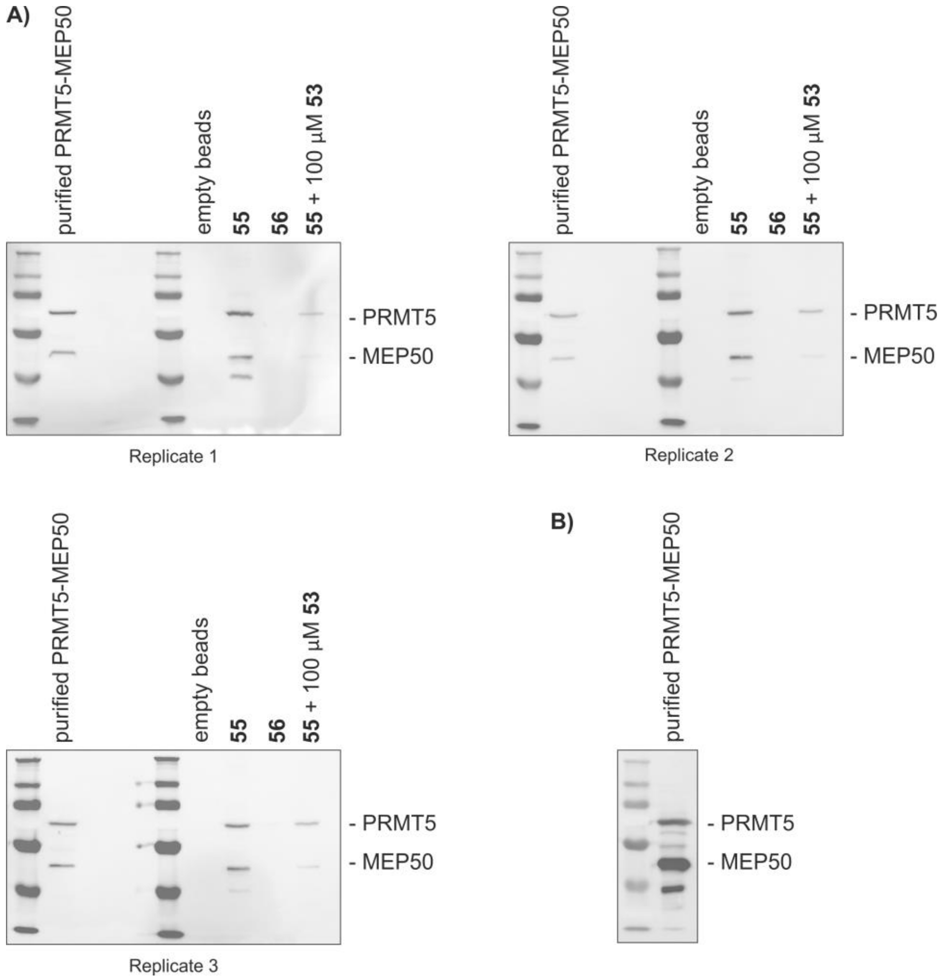

Figure S15. A) Results of the pull-down assay with peptide **55** and scrambled peptide **56** immobilised on the DBCO beads, using MCF7 cell lysate. **B)** Western blot of the purified PRMT5-MEP50 complex analysed at a higher concentration than in A).

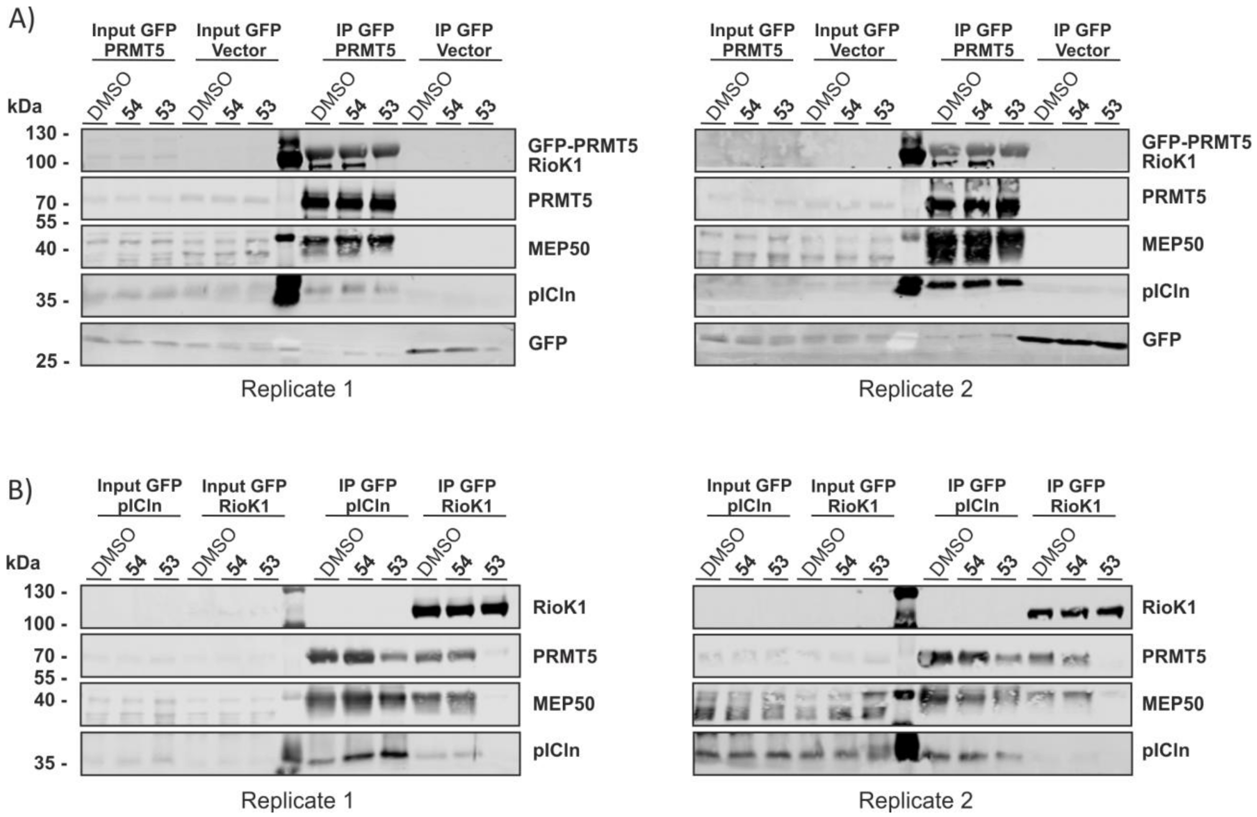

Figure S16. A) GFP-Immunopurification (GFP-IP) in Flp-In T-REx 293-GFP and Flp-In T-REx 293-GFP-PRMT5 overexpressing cells after testing active **53** and scrambled **54** at 50 µM and DMSO as a control. Therefore, cells were stimulated with 0.1 µg/ml doxycycline for 18 h before cytoplasm extraction (S100). GFP-IP was performed and analyzed by Tris/Glycine-SDS-PAGE and western blotting using antibodies against RioK1, PRMT5, MEP50, pICln and GFP. **B)** GFP-IP from Flp-In T-REx 293-GFP-pICln and Flp-In T-Rex 293-GFP-RioK1 cytoplasmic extract after testing active **53** and scrambled **54** at 50 µM and DMSO as a control. Induction of the overexpression, Tris/Glycine-SDS-PAGE and western blotting using antibodies against RioK1, PRMT5, MEP50 and pICln was performed as described in **A**.

HPLC Traces

Peptide 1 (Method A)

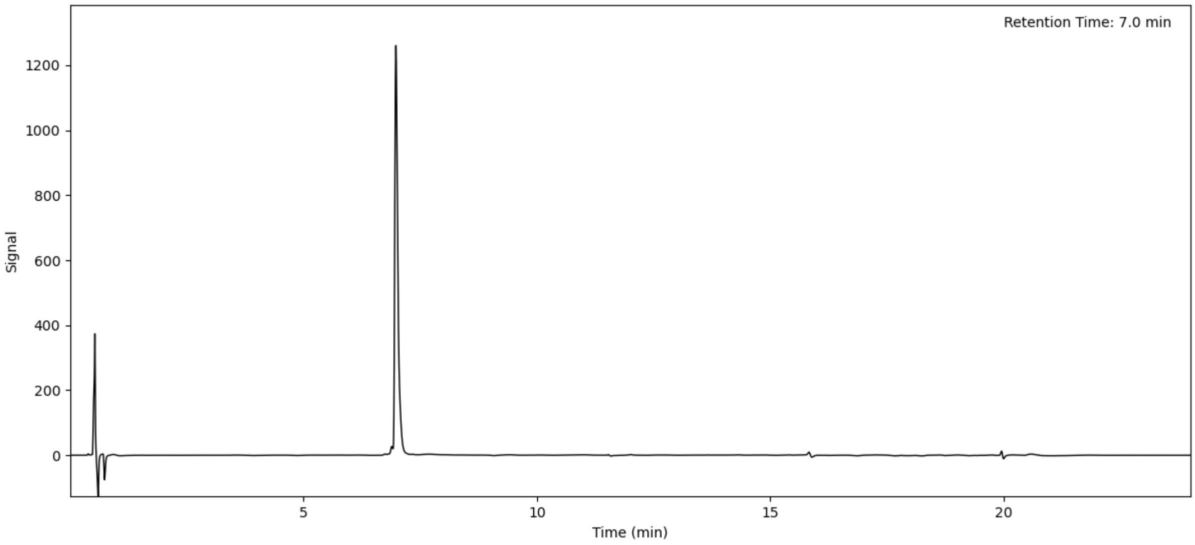

Peptide 2 (Method A)

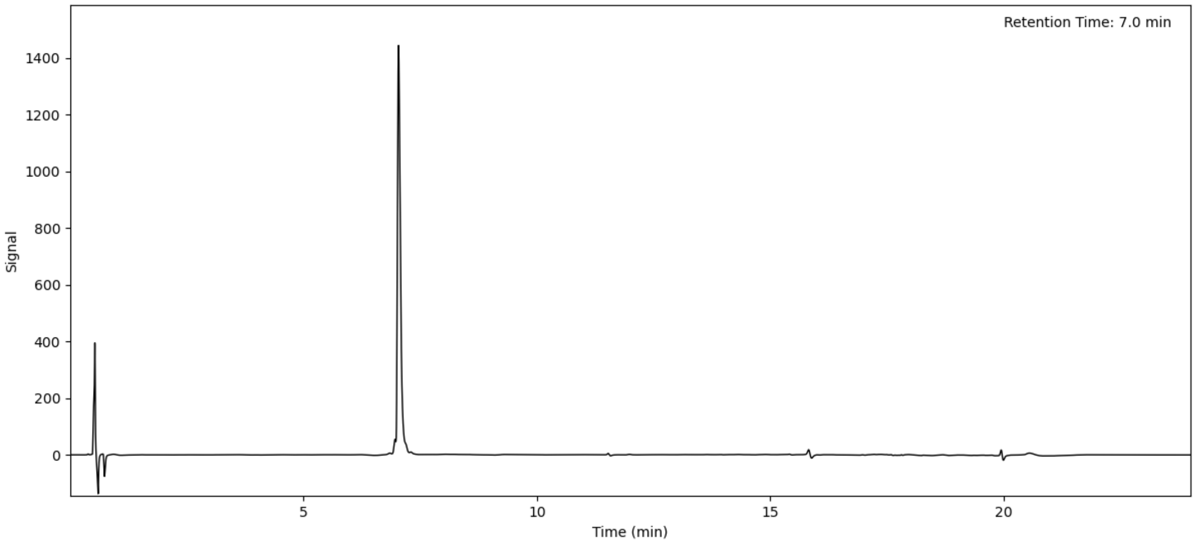

Peptide 3 (Method A)

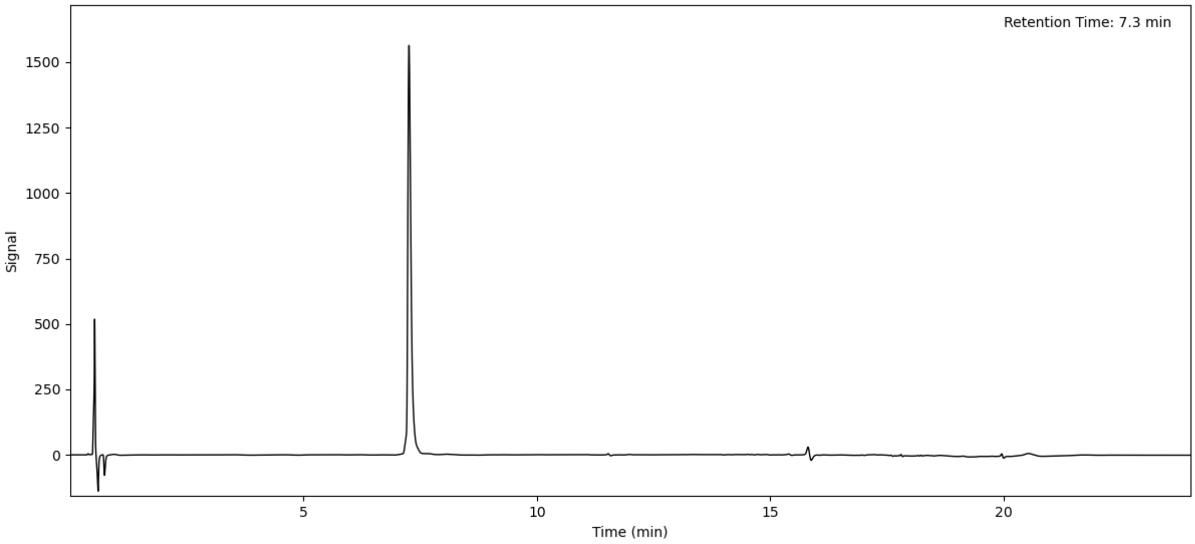

Peptide 4 (Method A)

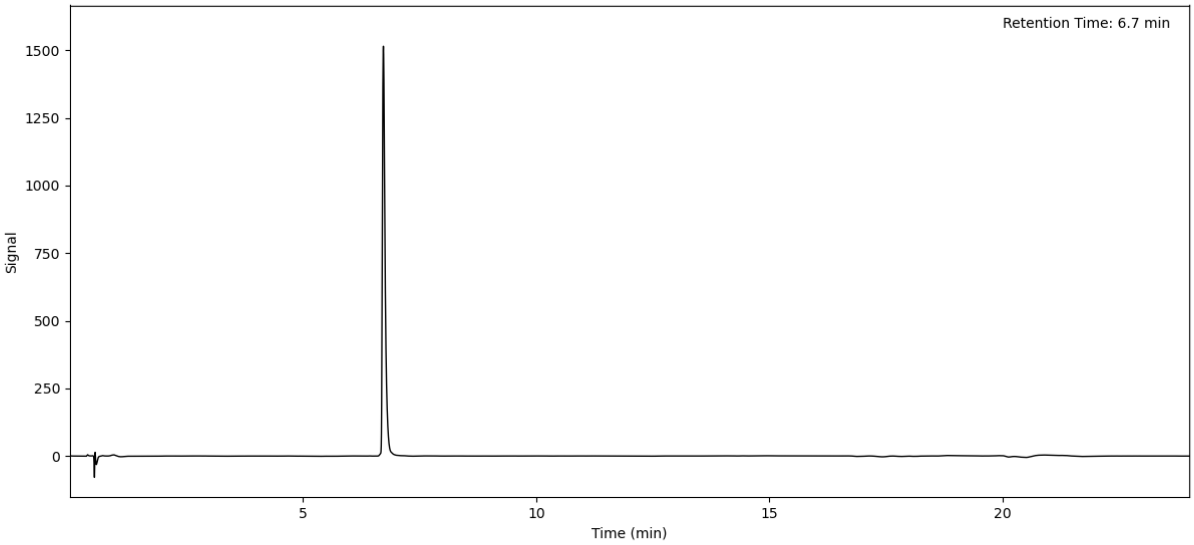

Peptide 5 (Method A)

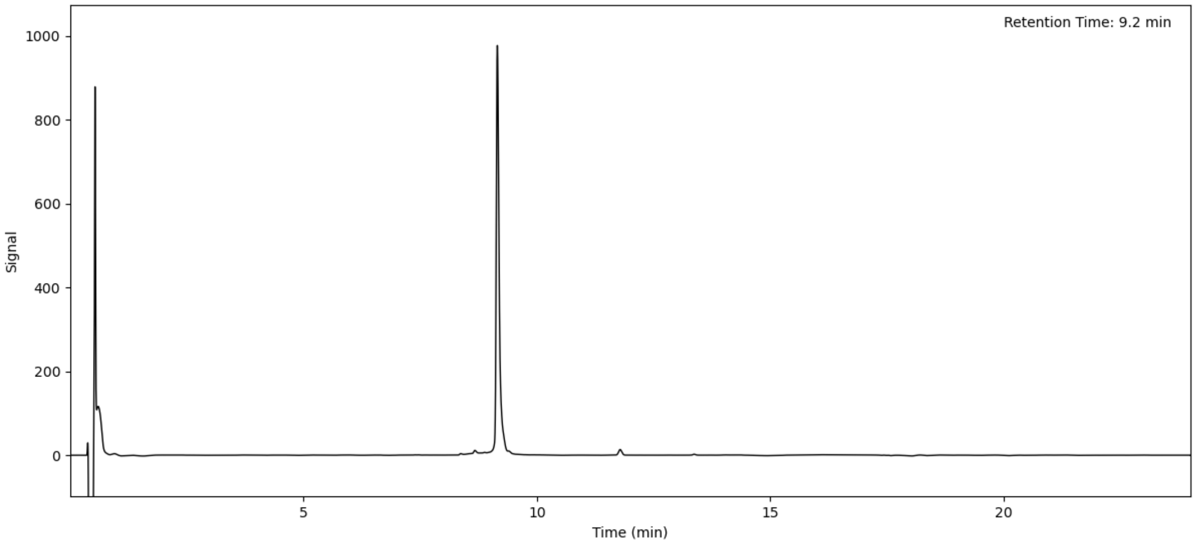

Peptide 6 (Method A)

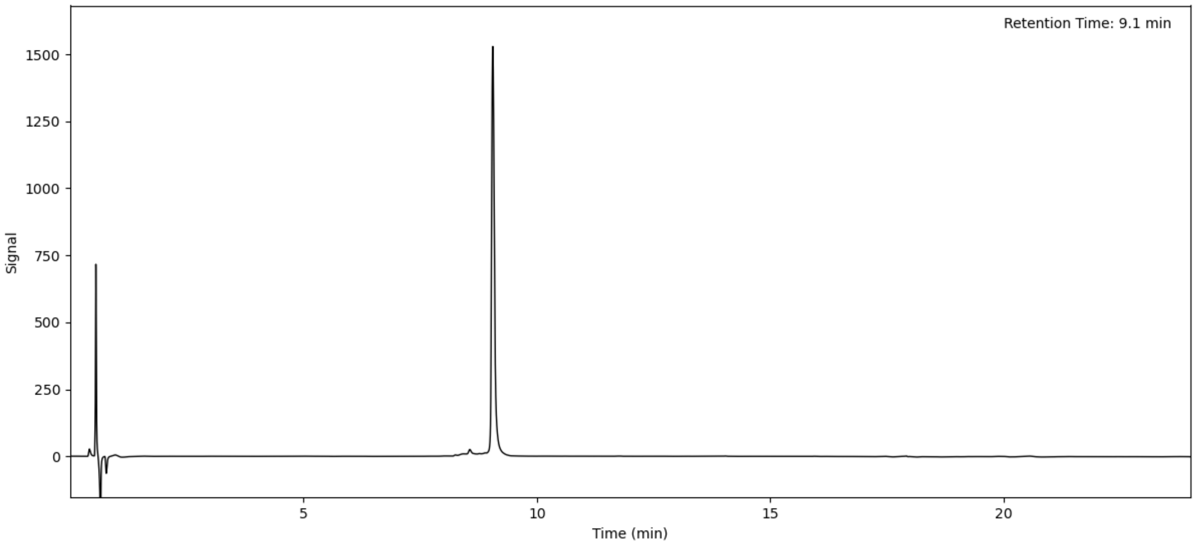

Peptide 7 (Method A)

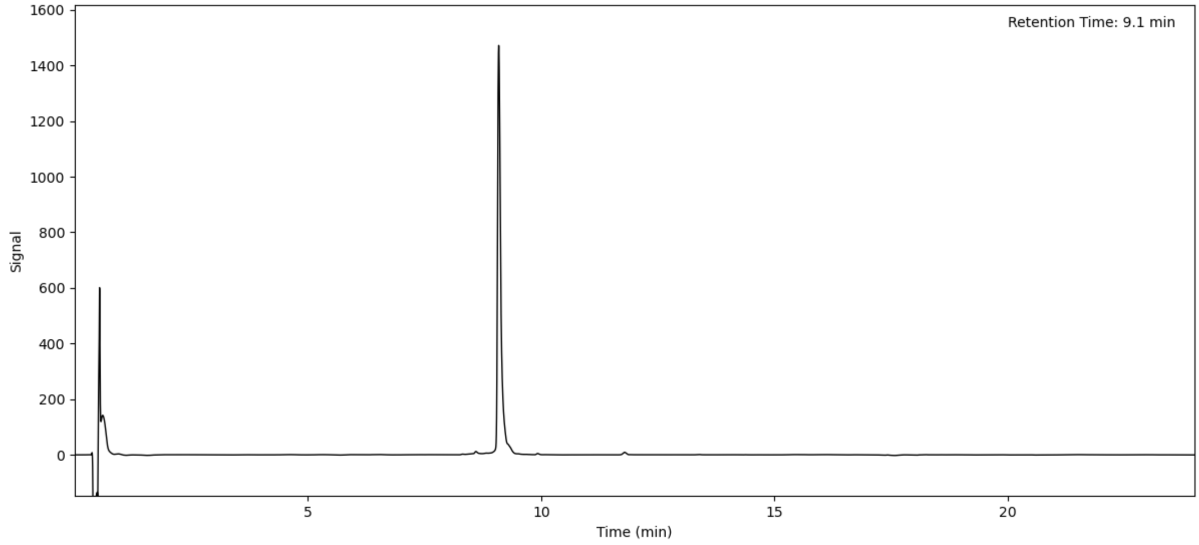

Peptide 8 (Method A)

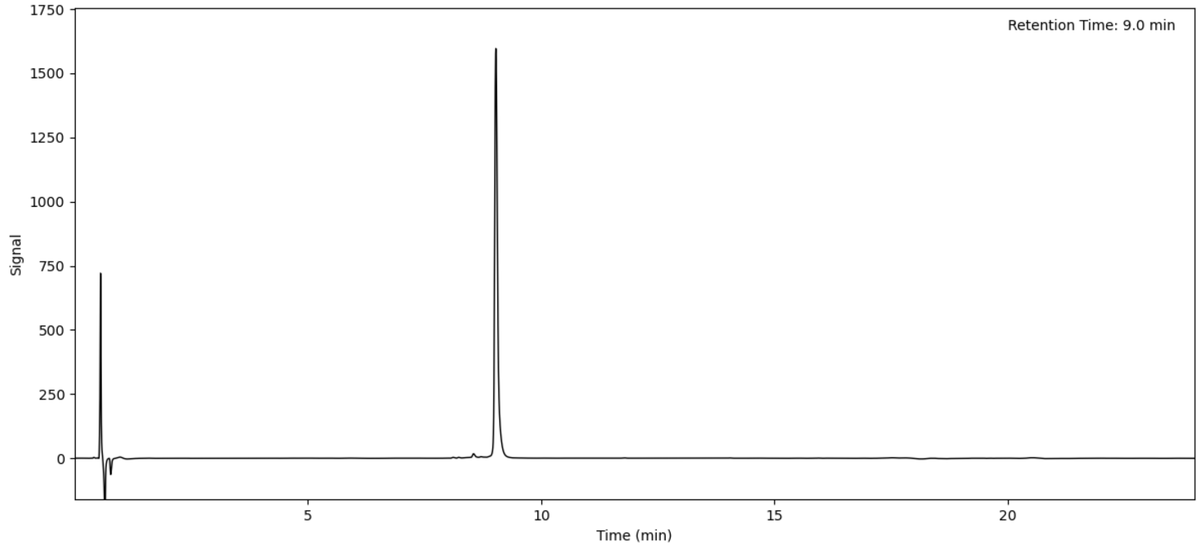

Peptide 9 (Method A)

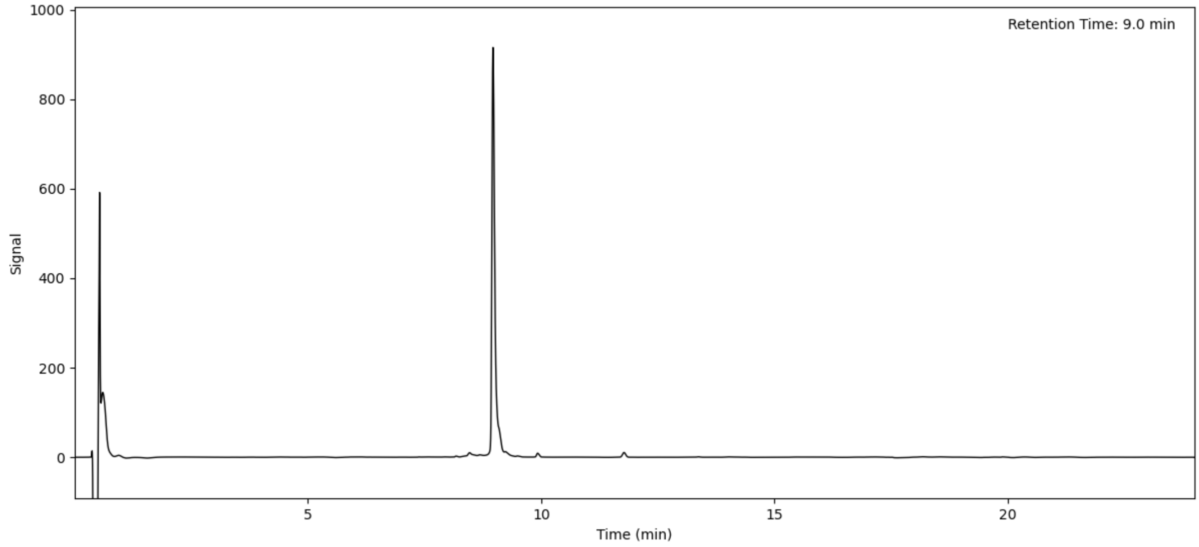

Peptide 10 (Method A)

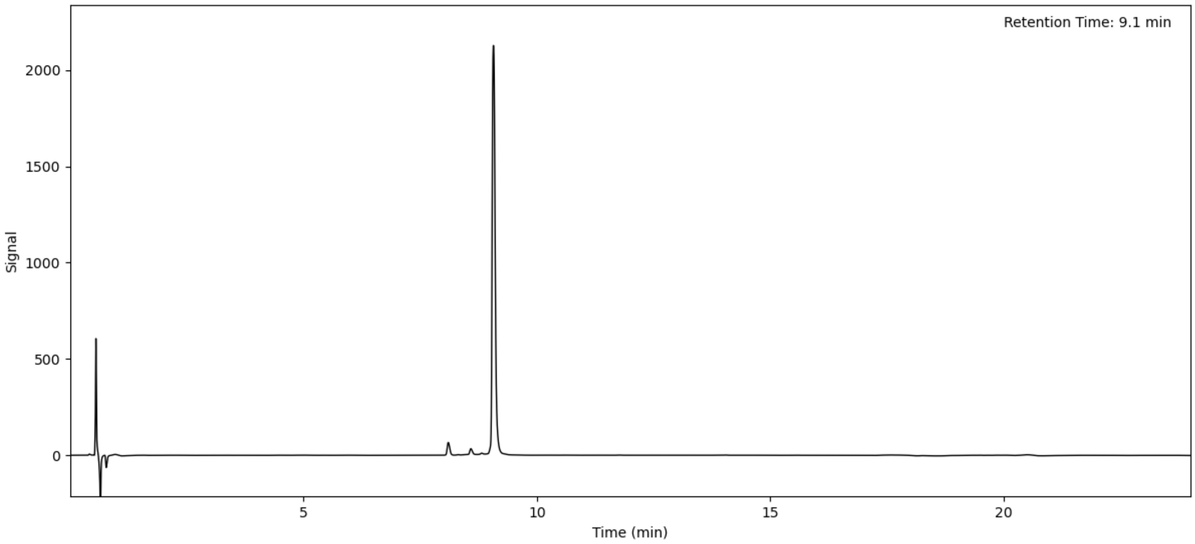

Peptide 11 (Method A)

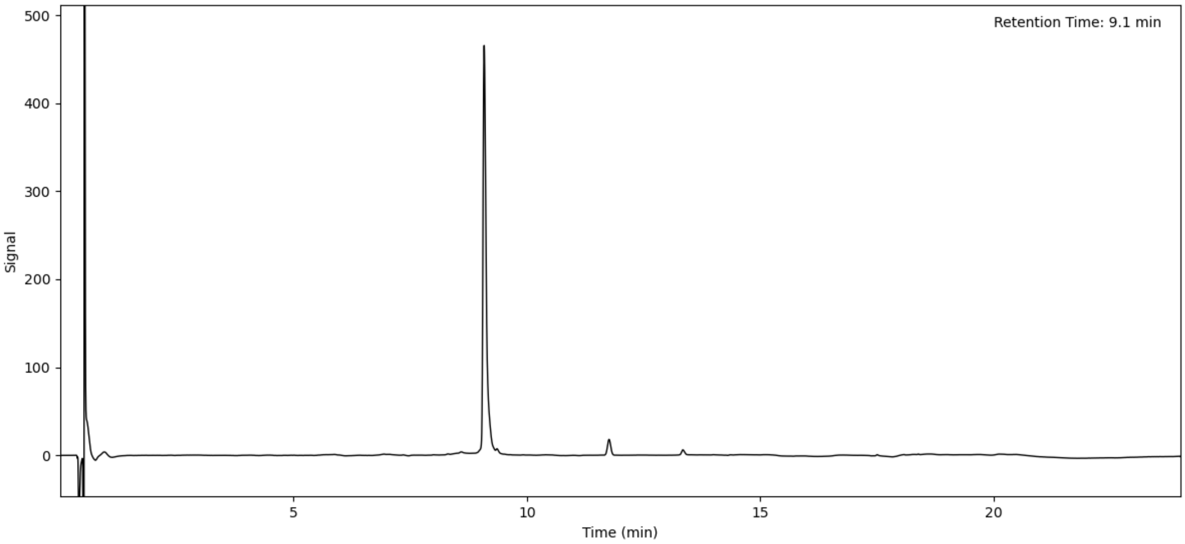

Peptide 12 (Method A)

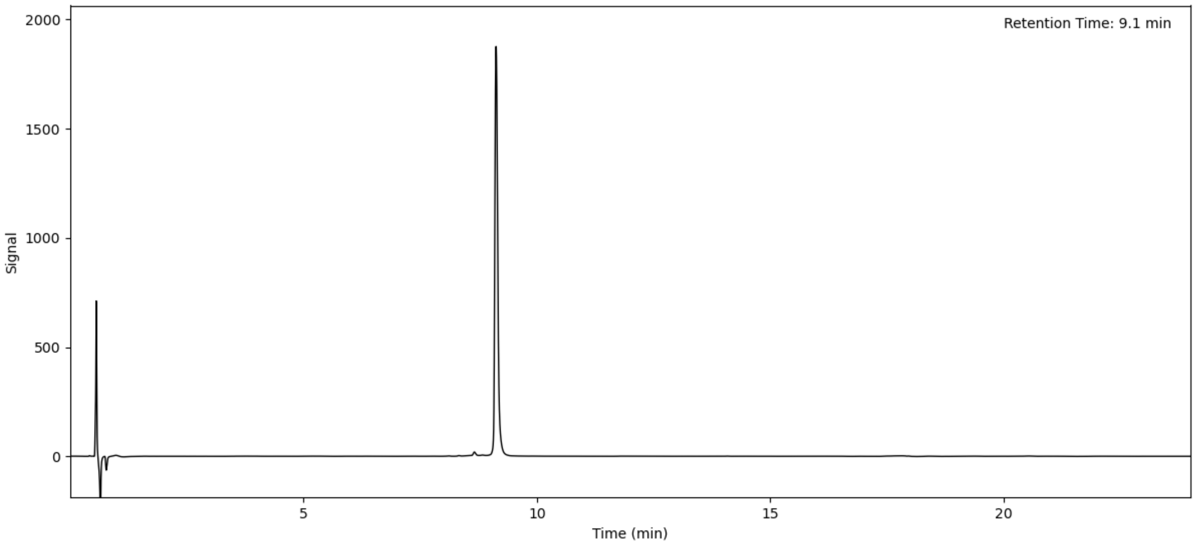

Peptide 13 (Method A)

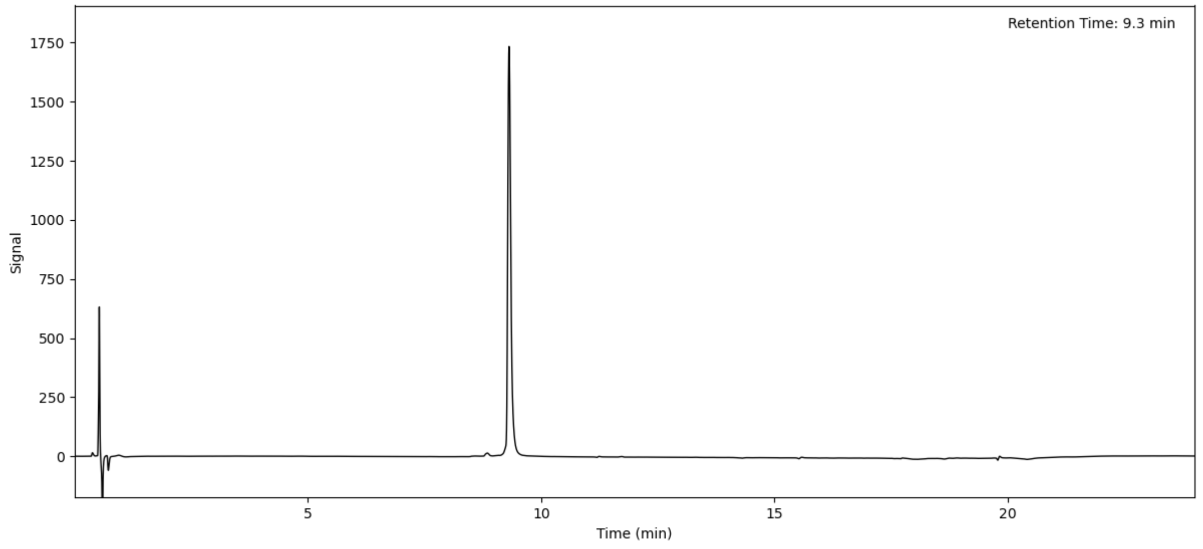

Peptide 14 (Method A)

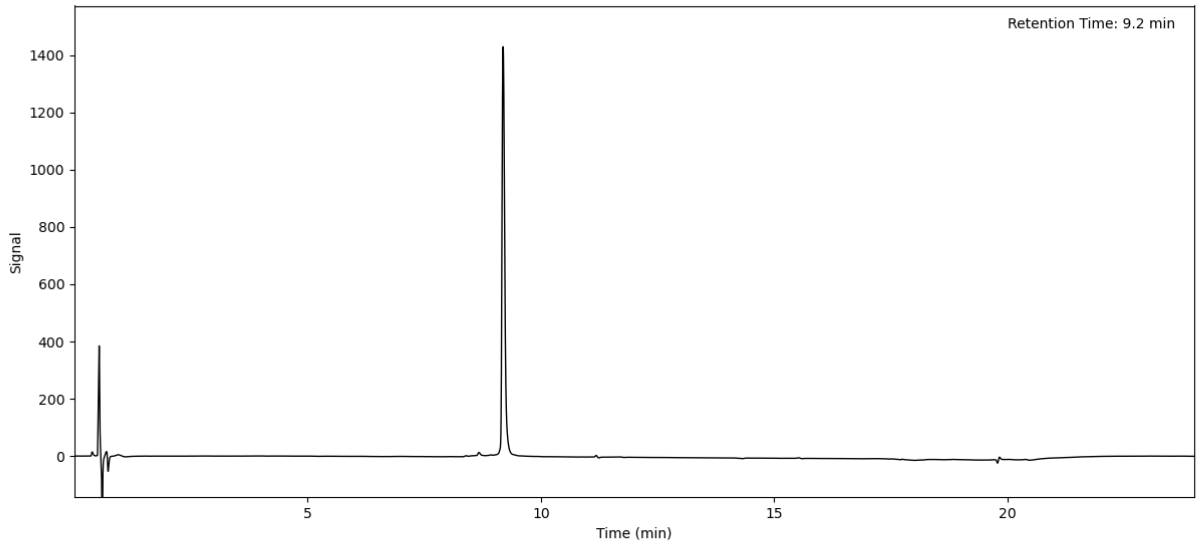

Peptide 15 isomer 1 (Method A)

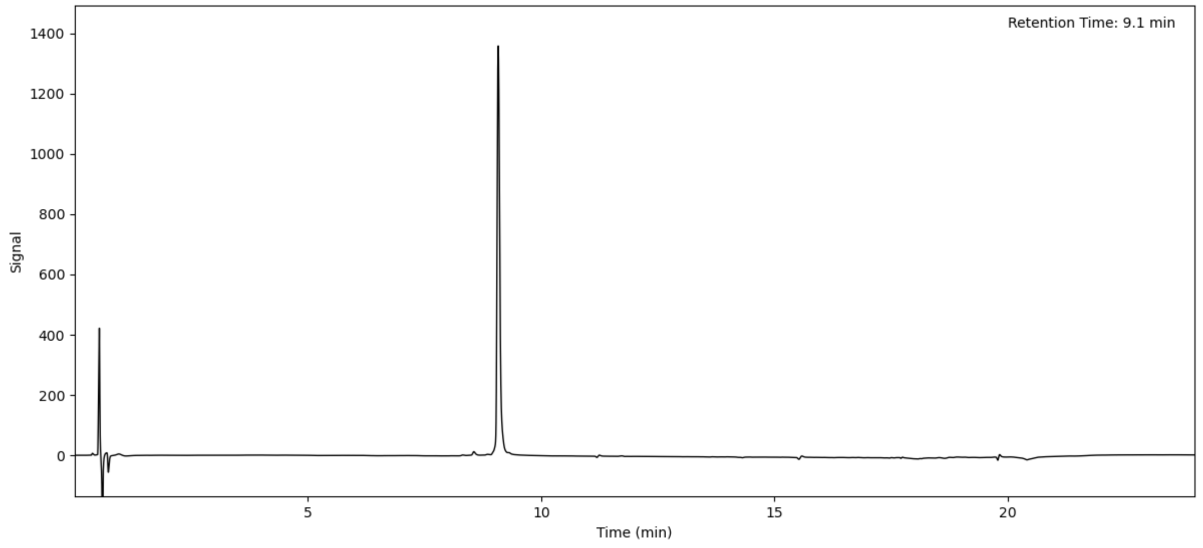

Peptide 15 isomer 2 (Method A)

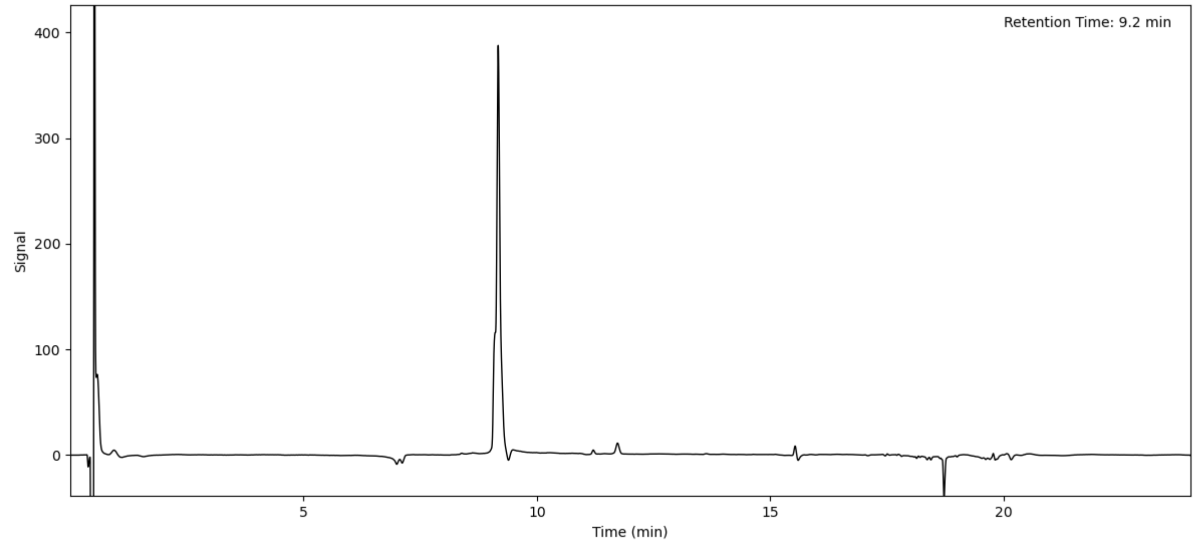

Peptide 16 (Method A)

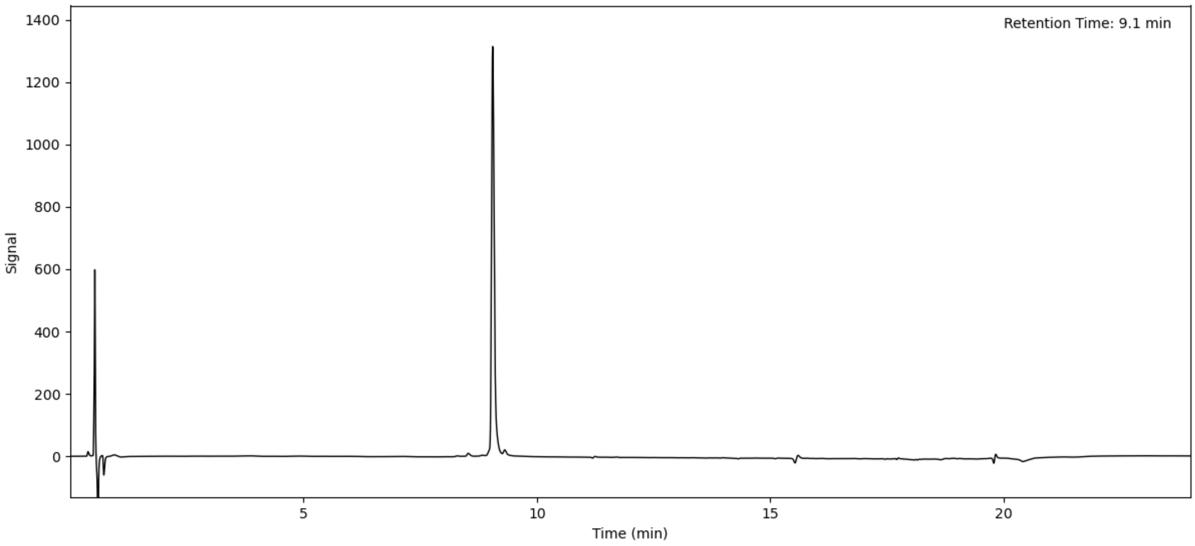

Peptide 17 (Method A)

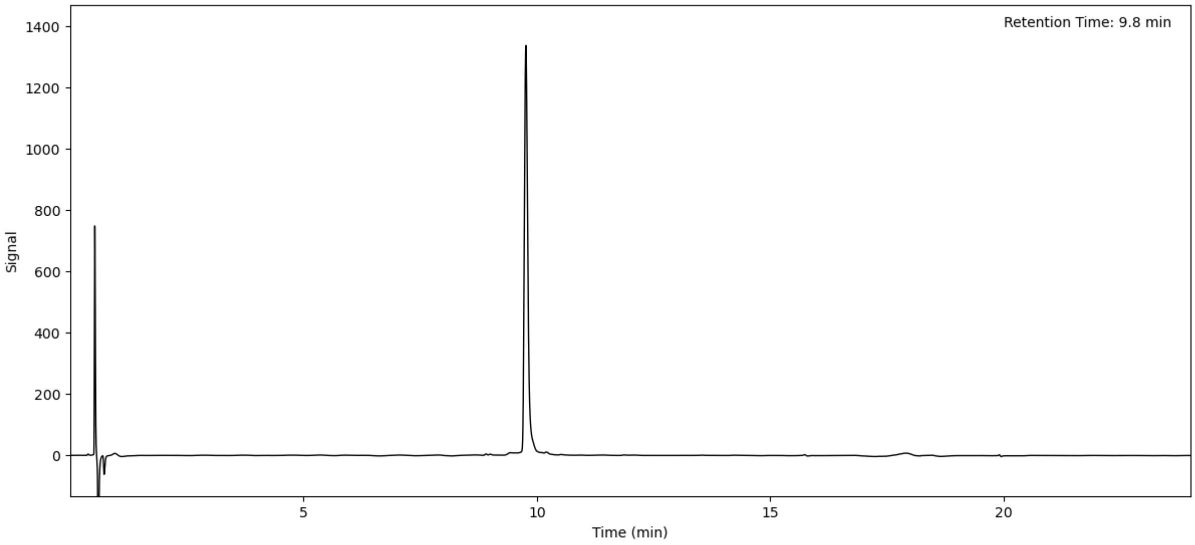

Peptide 18 isomer 1 (Method A)

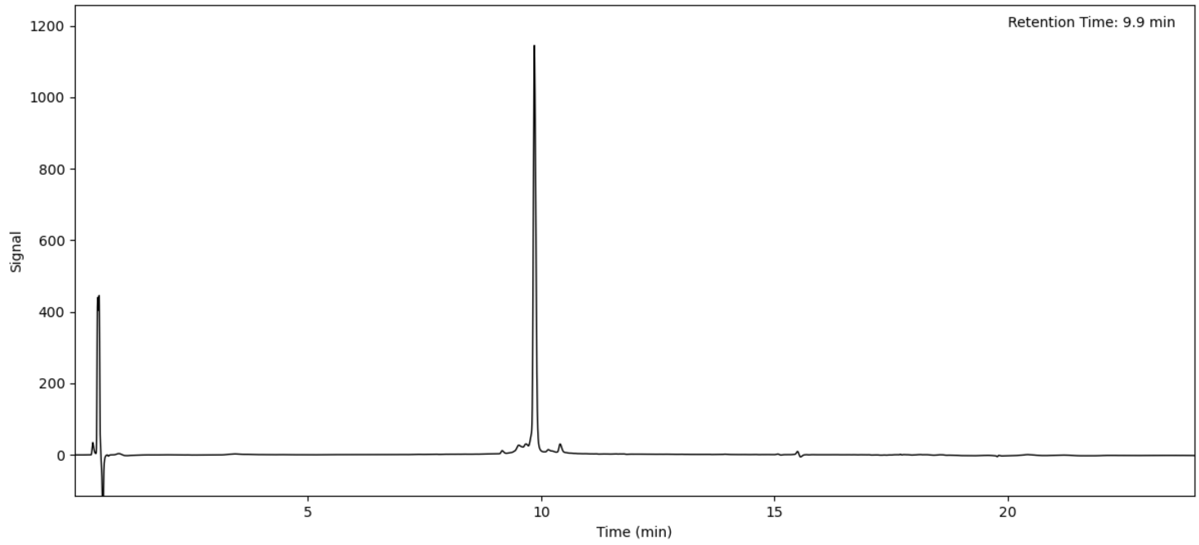

Peptide 18 isomer 2 (Method A)

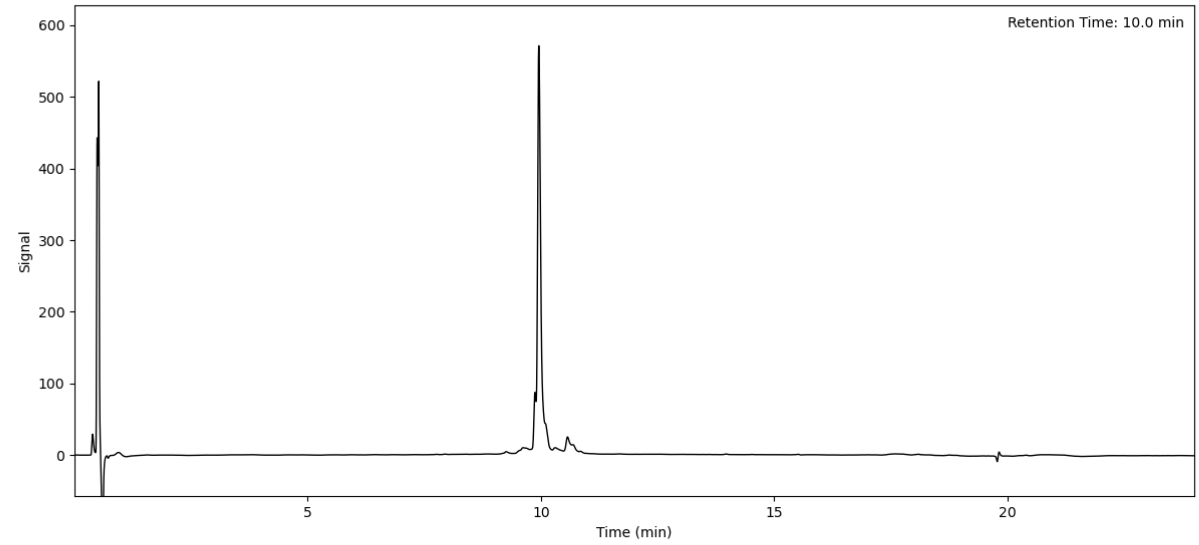

Peptide 19 (Method A)

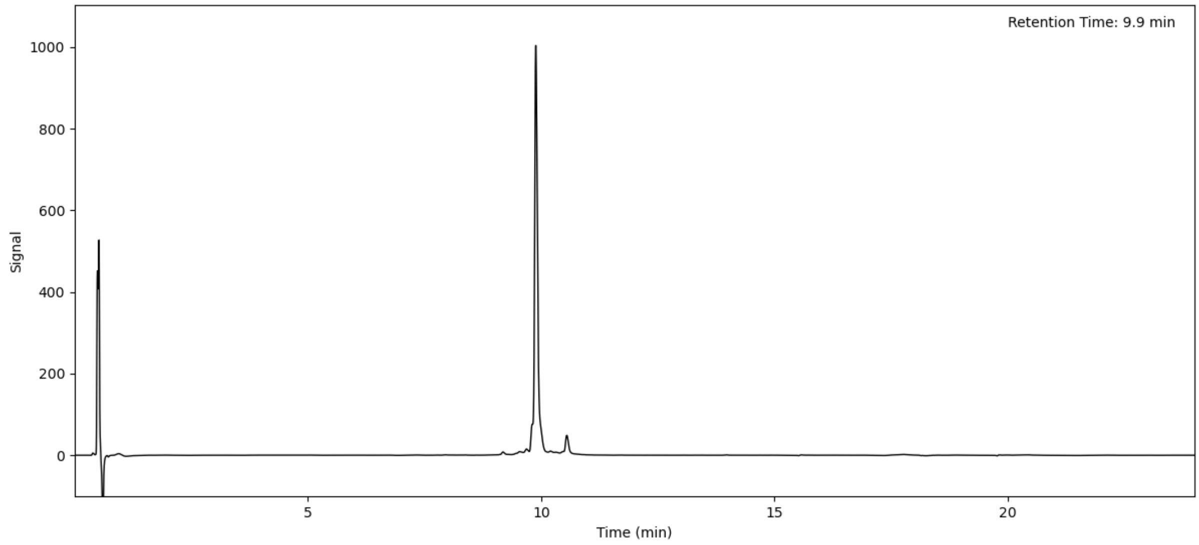

Peptide 20 (Method A)

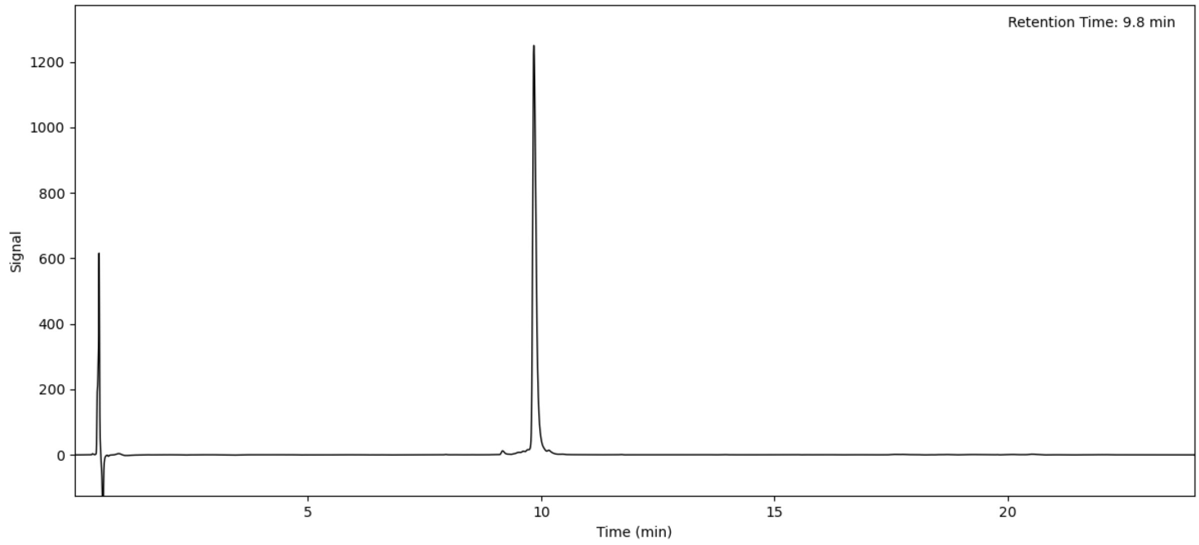

Peptide 21 (Method A)

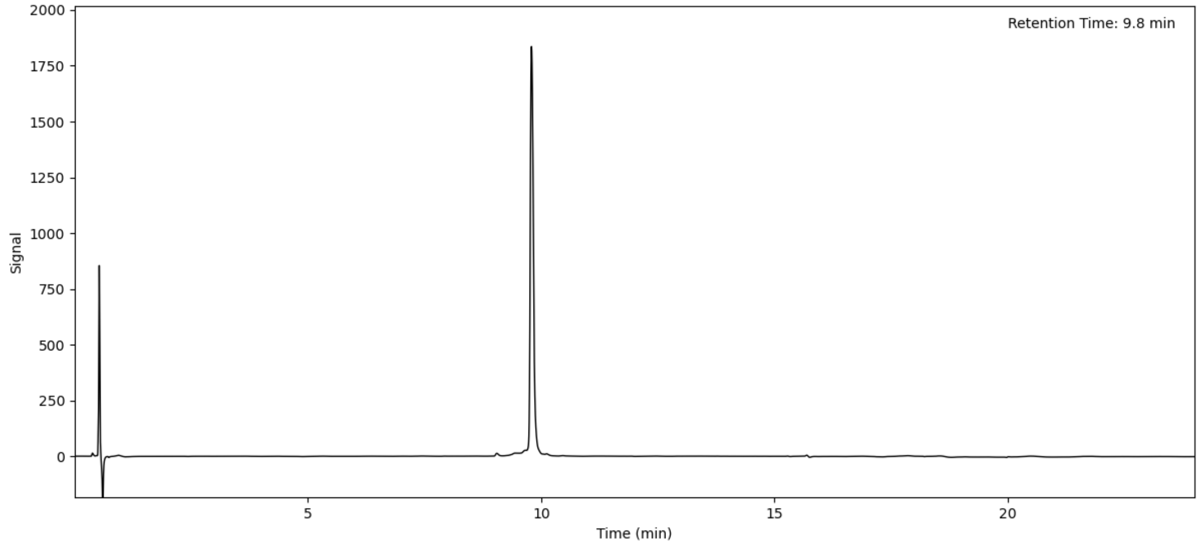

Peptide 22 (Method A)

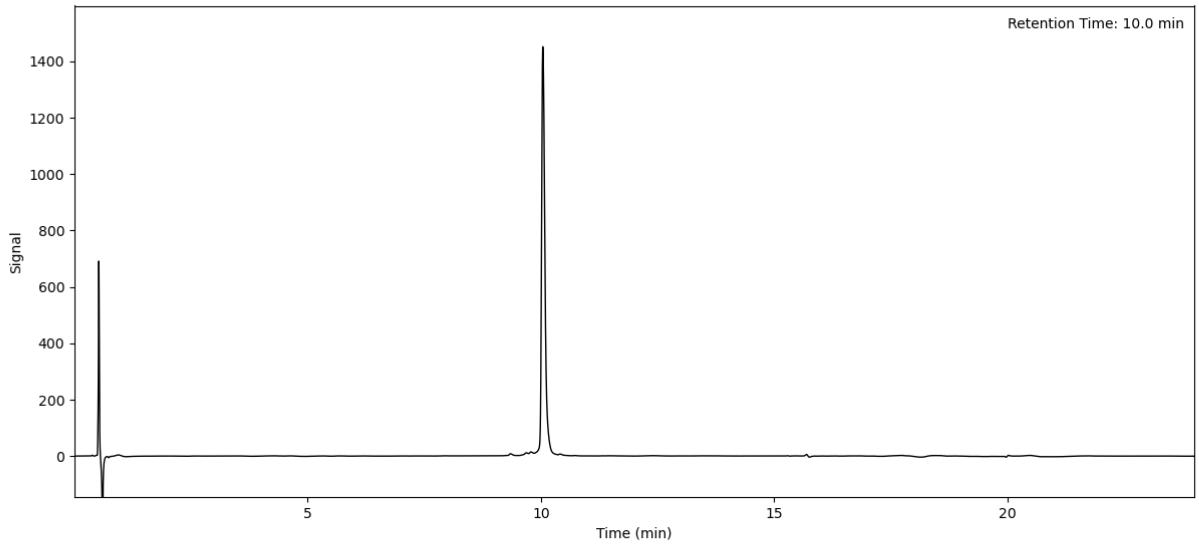

Peptide 23 (Method A)

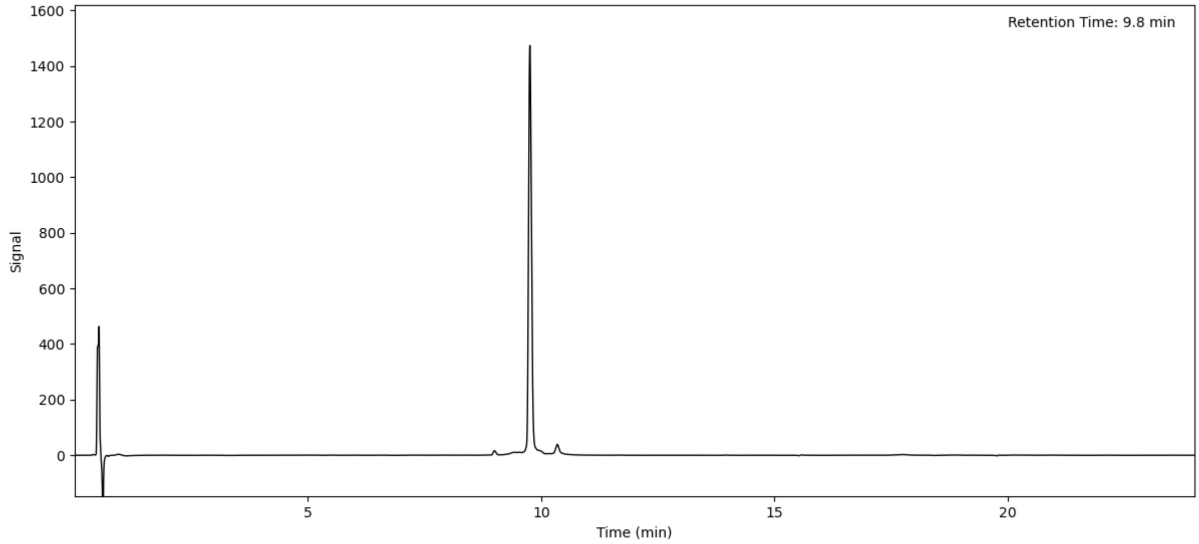

Peptide 24 (Method A)

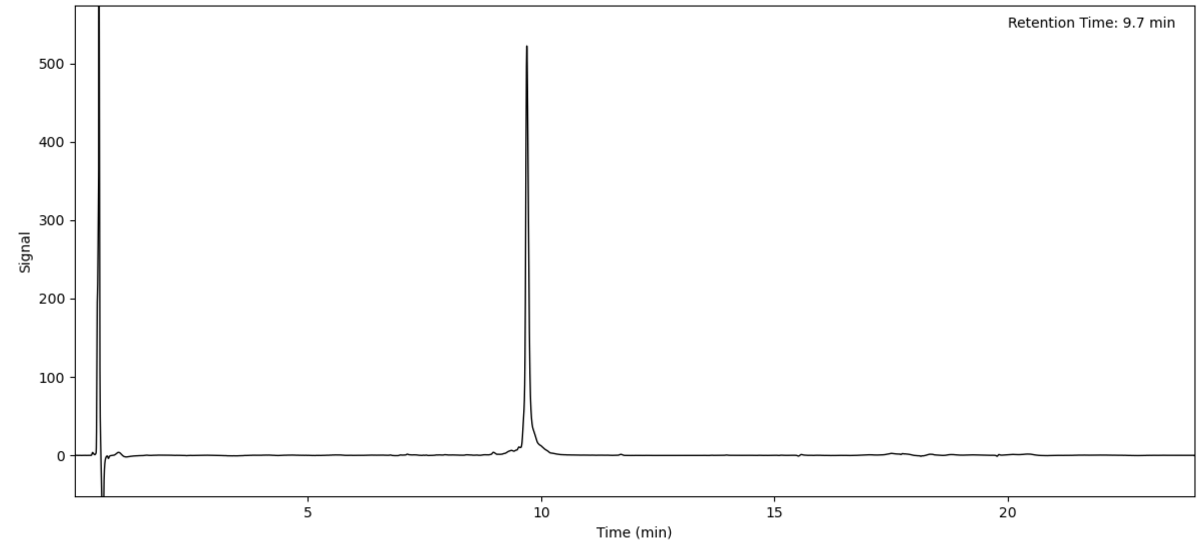

Peptide 25 (Method A)

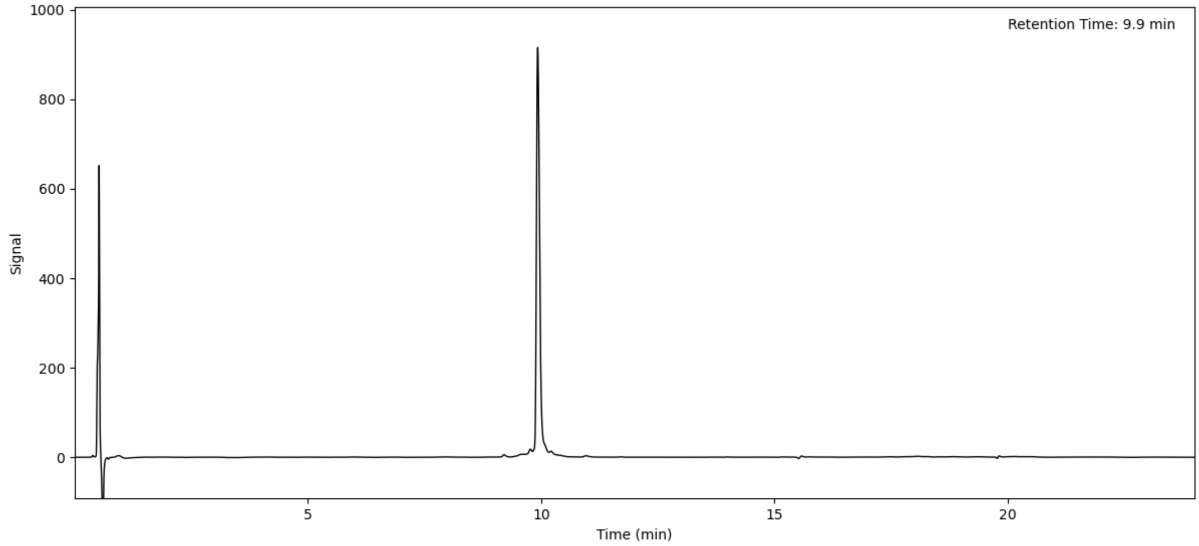

Peptide 26 (Method A)

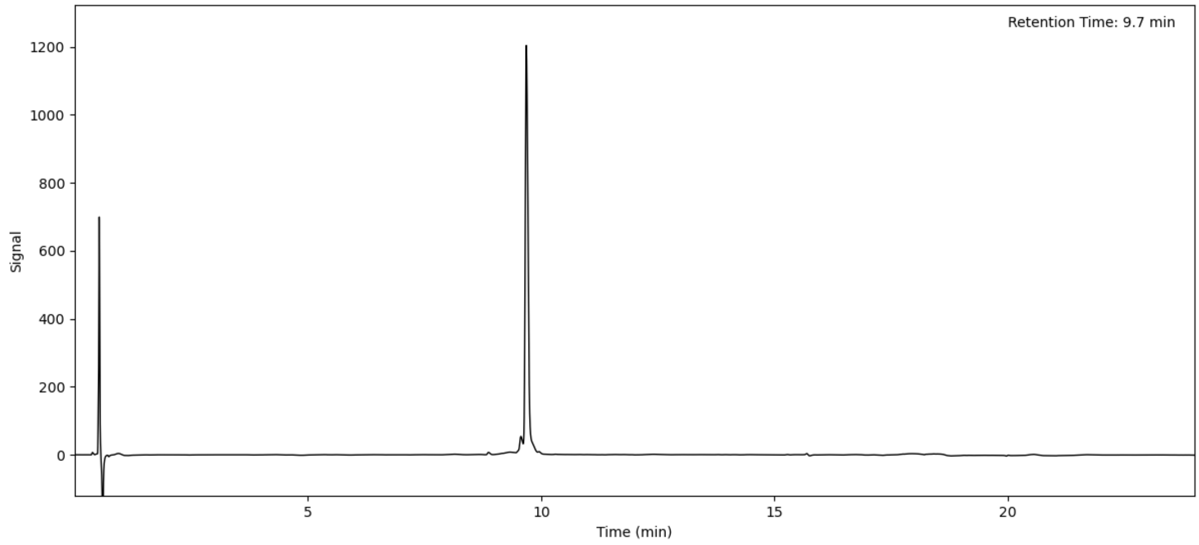

Peptide 27 (Method A)

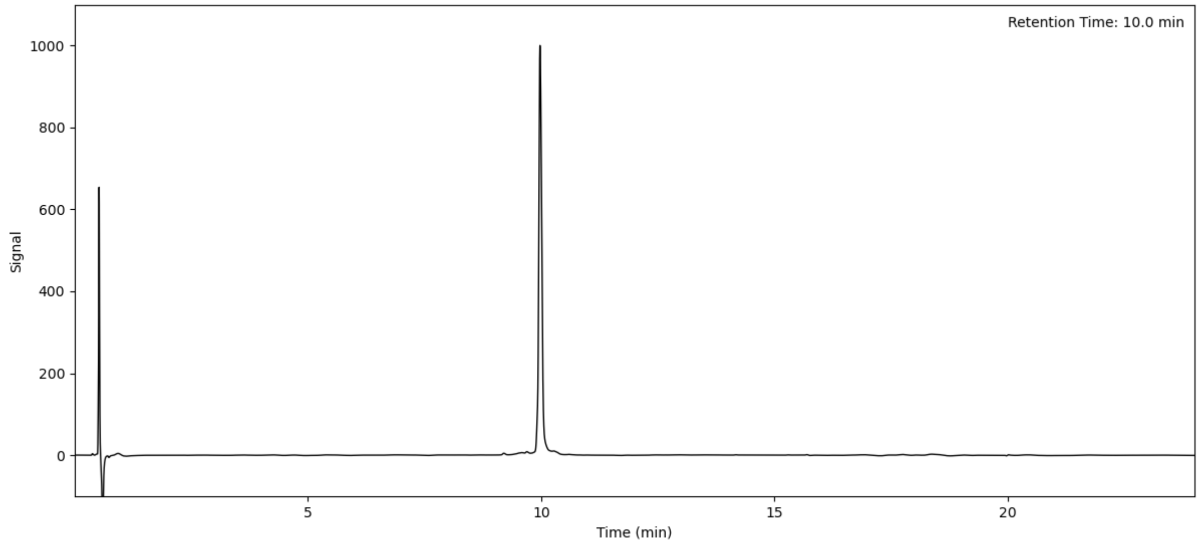

Peptide 28 (Method A)

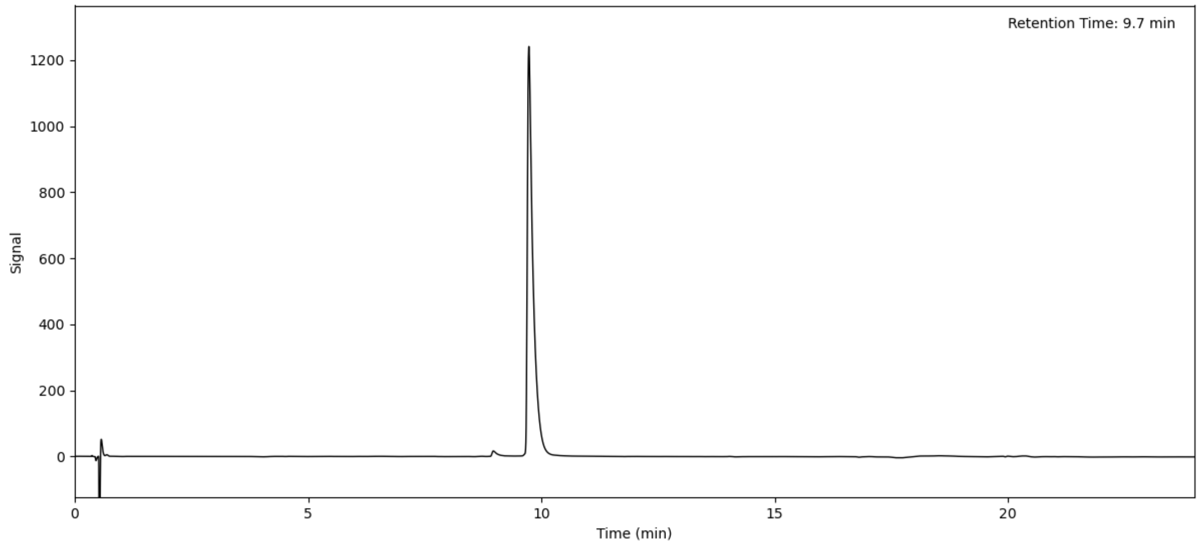

Peptide 29 (Method A)

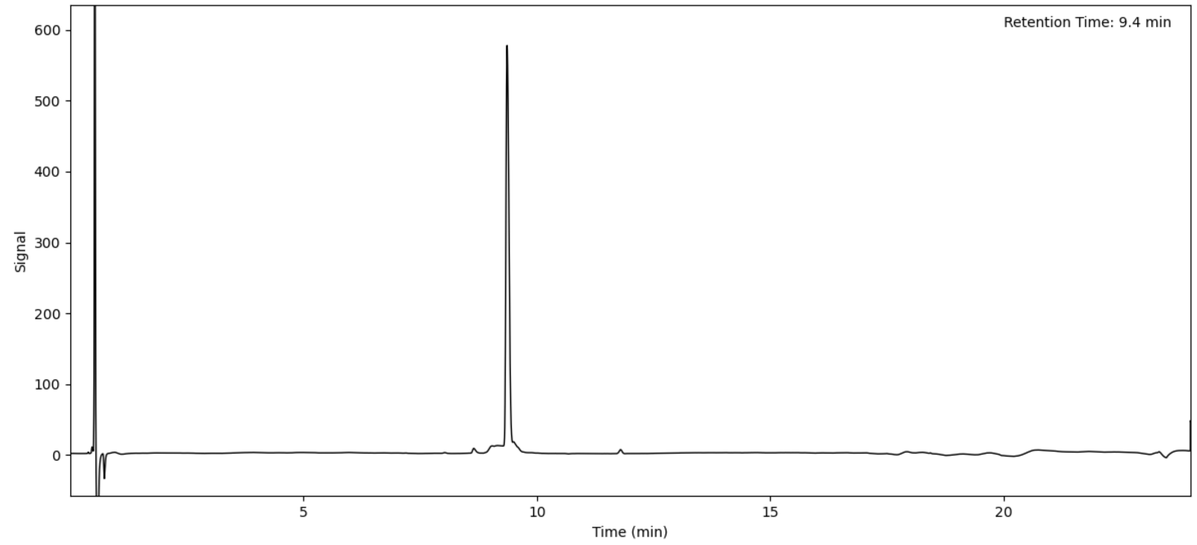

Peptide 30 (Method A)

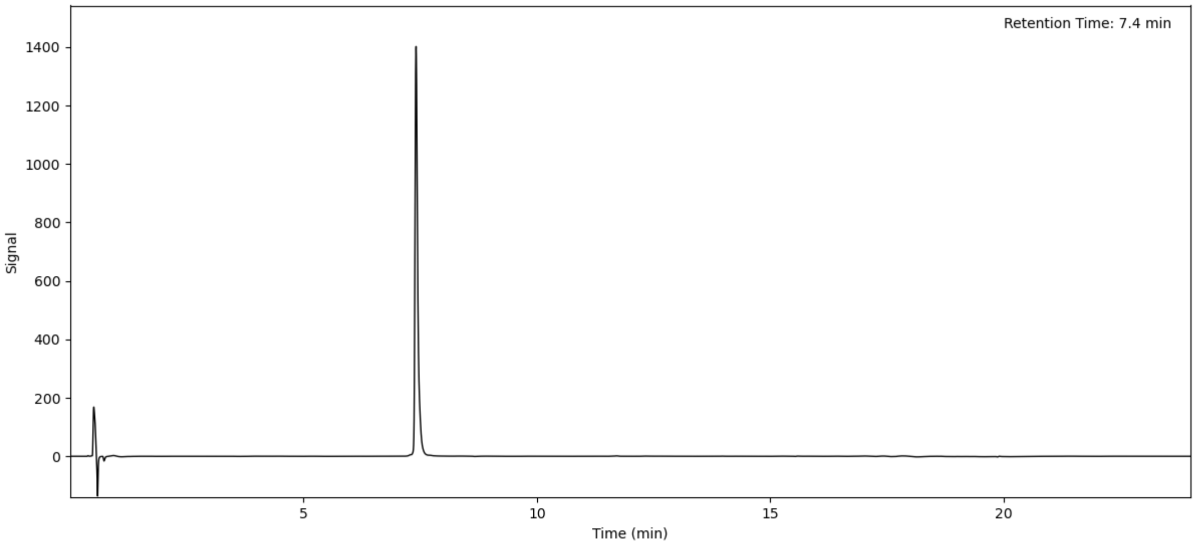

Peptide 31 (Method A)

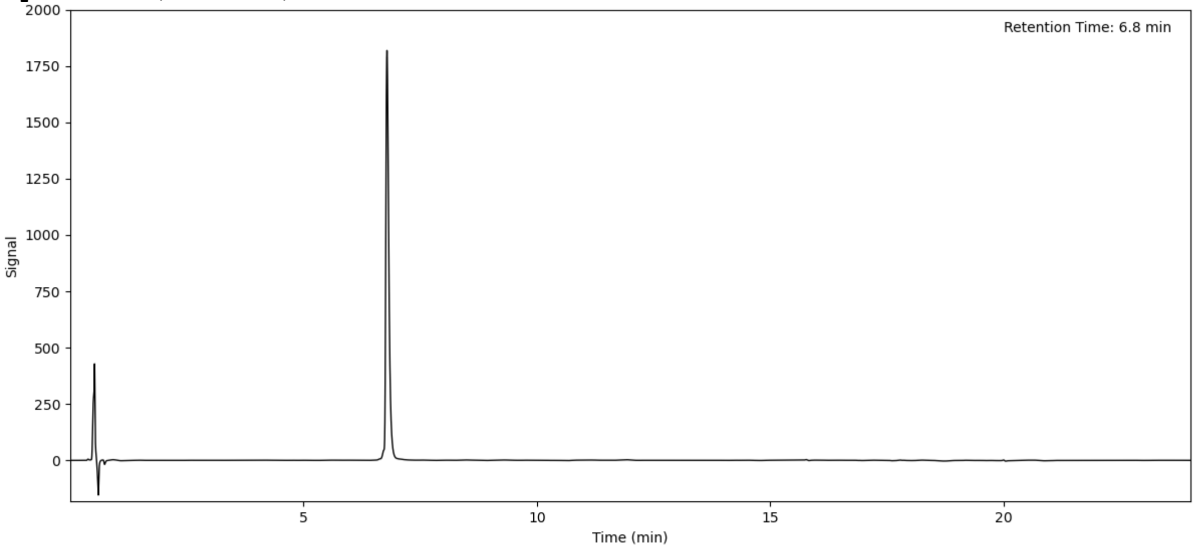

Peptide 32 (Method A)

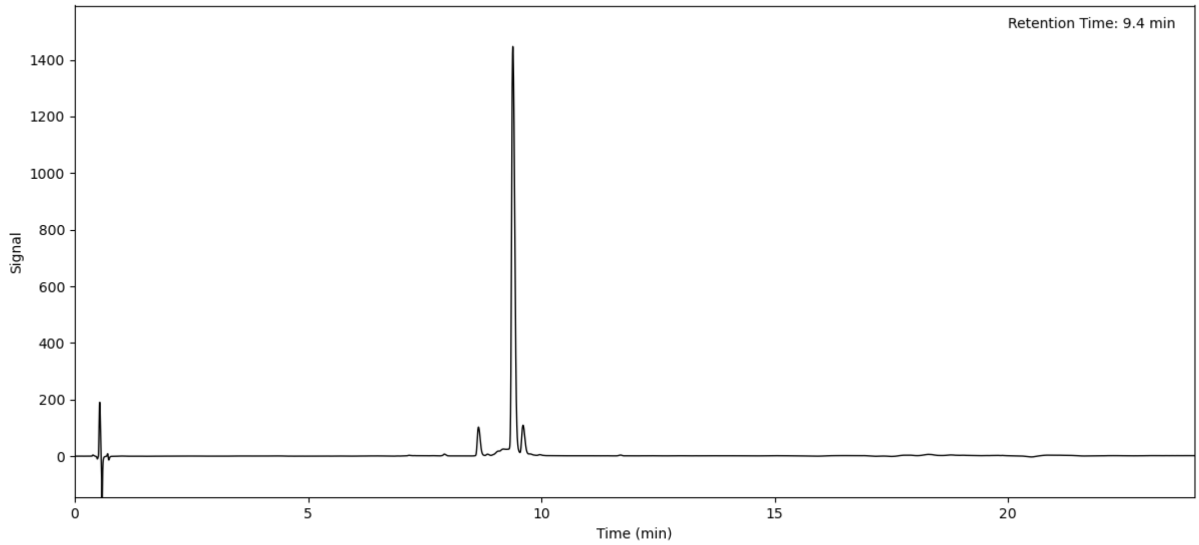

Peptide 33 (Method A)

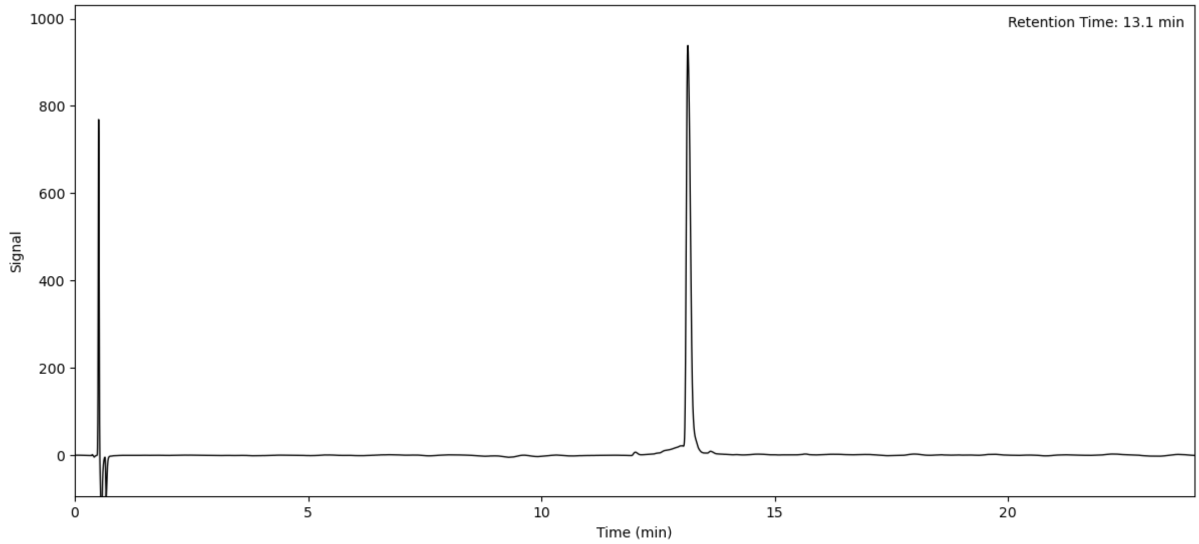

Peptide 34 (Method A)

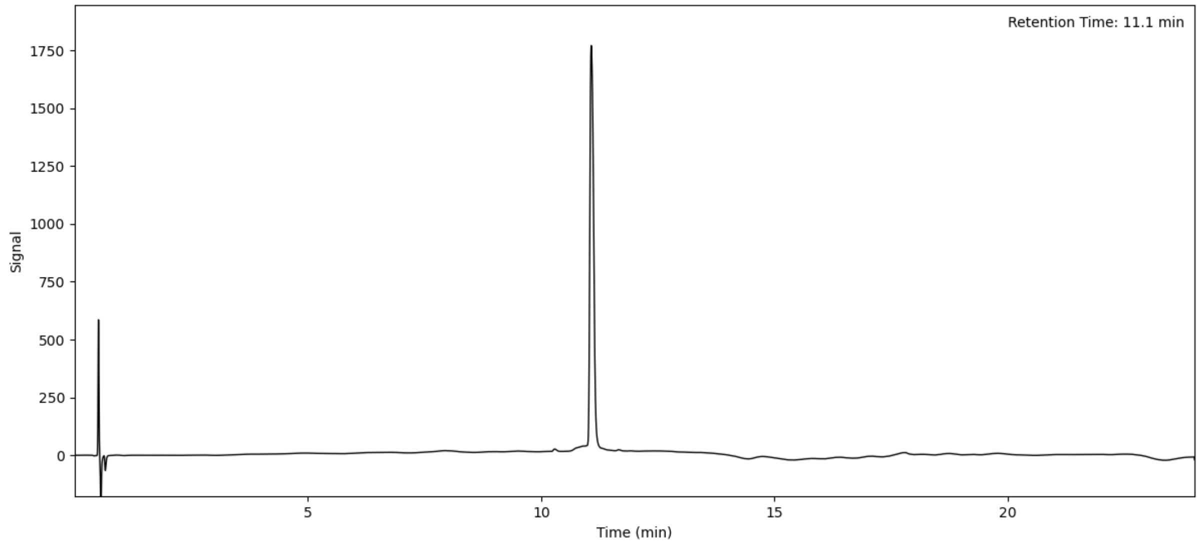

Peptide 35 (Method A)

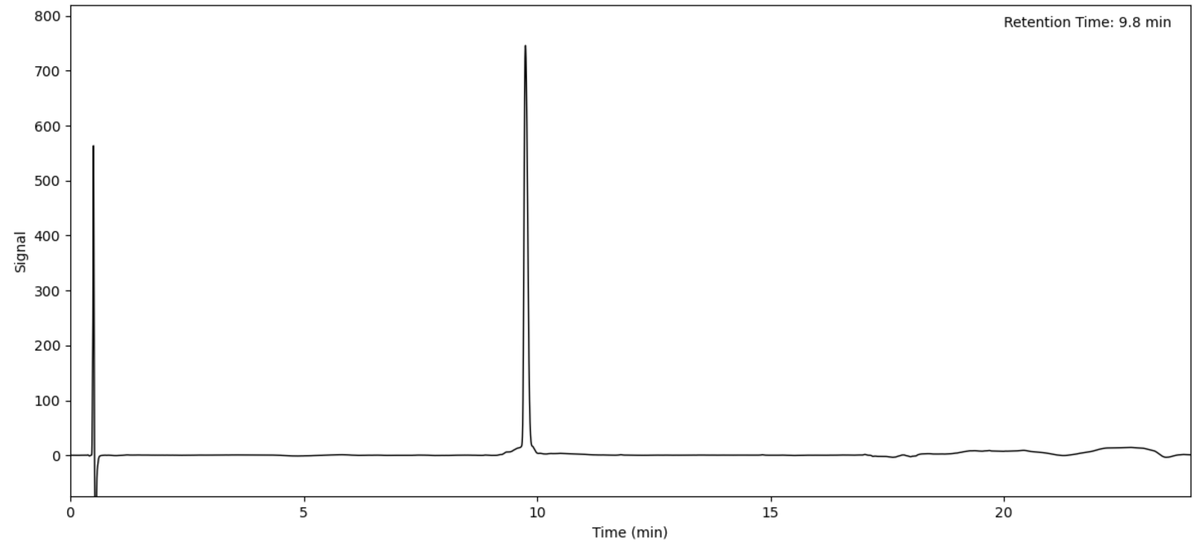

Peptide 36 (Method A)

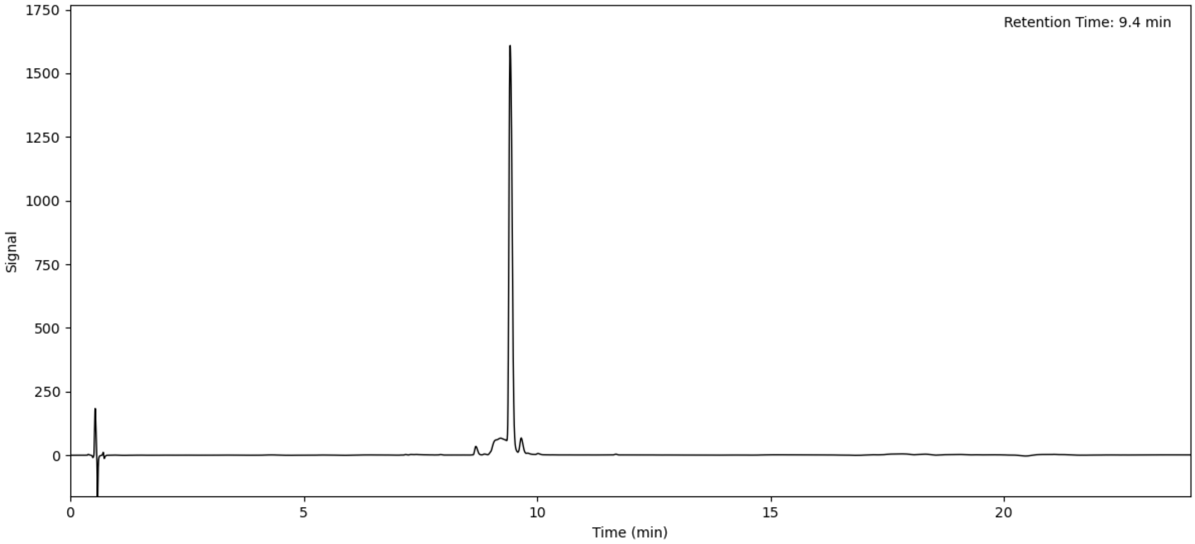

Peptide 37 (Method A)

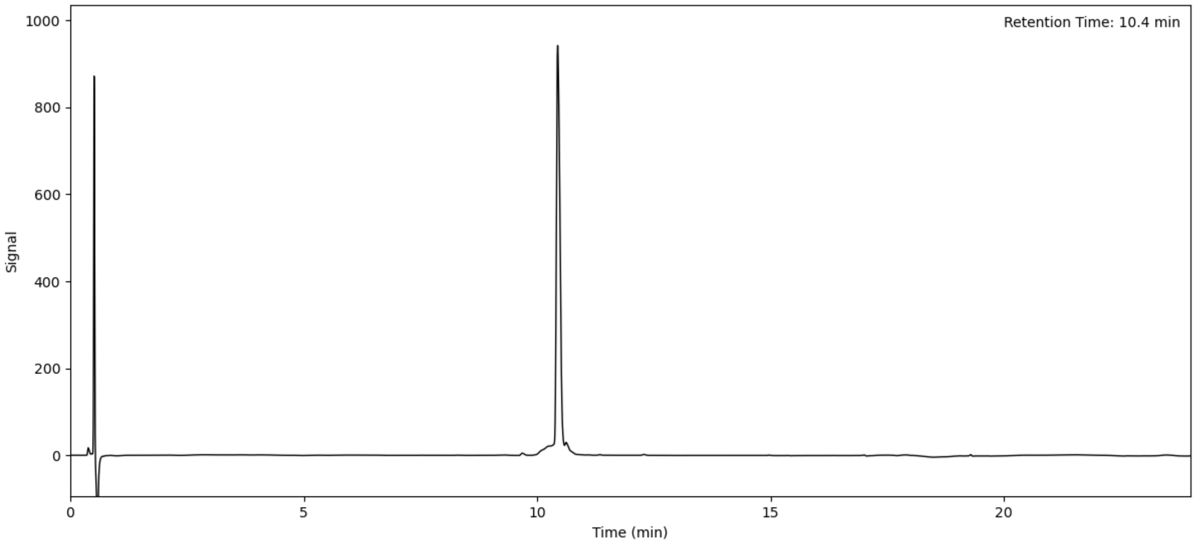

Peptide 38 (Method A)

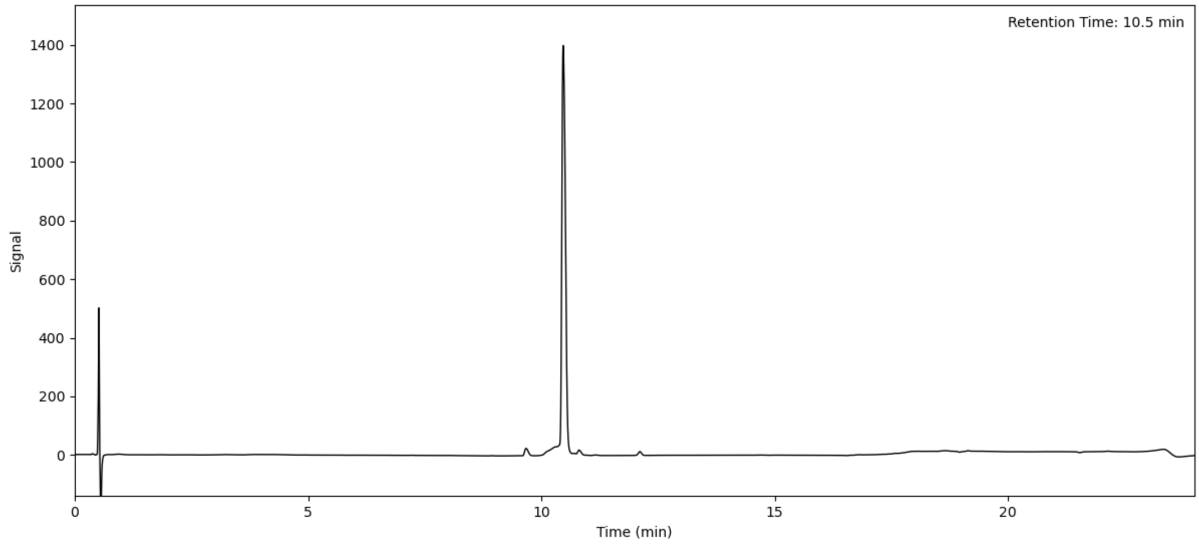

Peptide 39 (Method A)

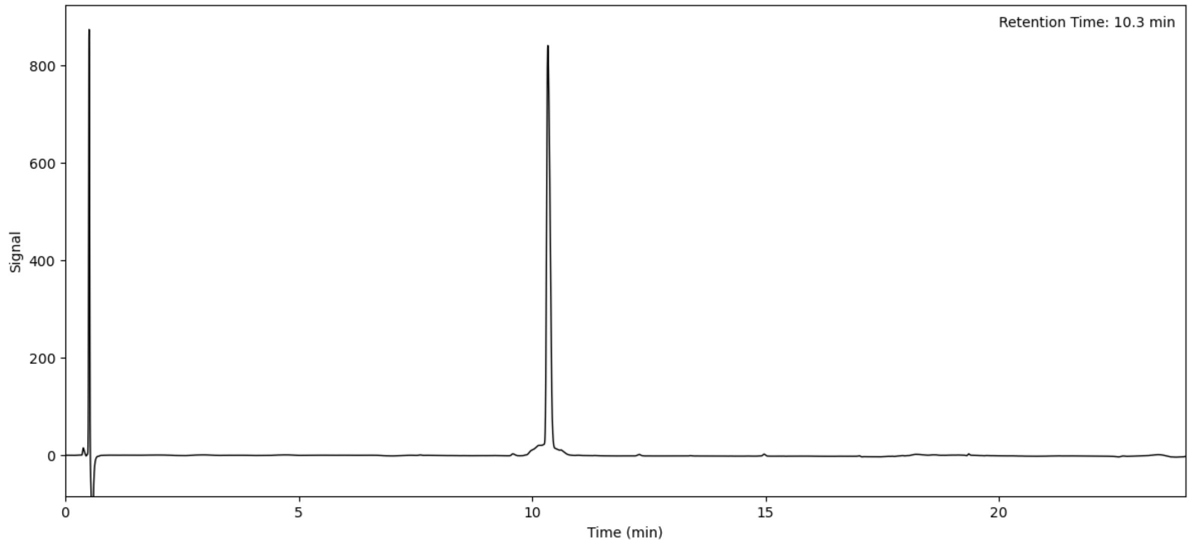

Peptide 40 (Method A)

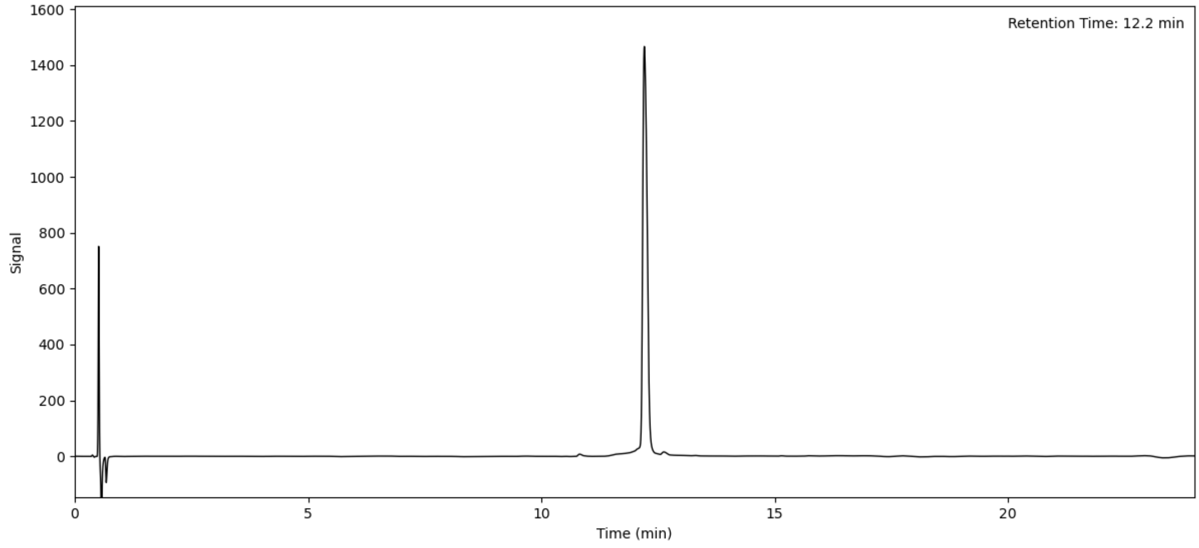

Peptide 41 (Method A)

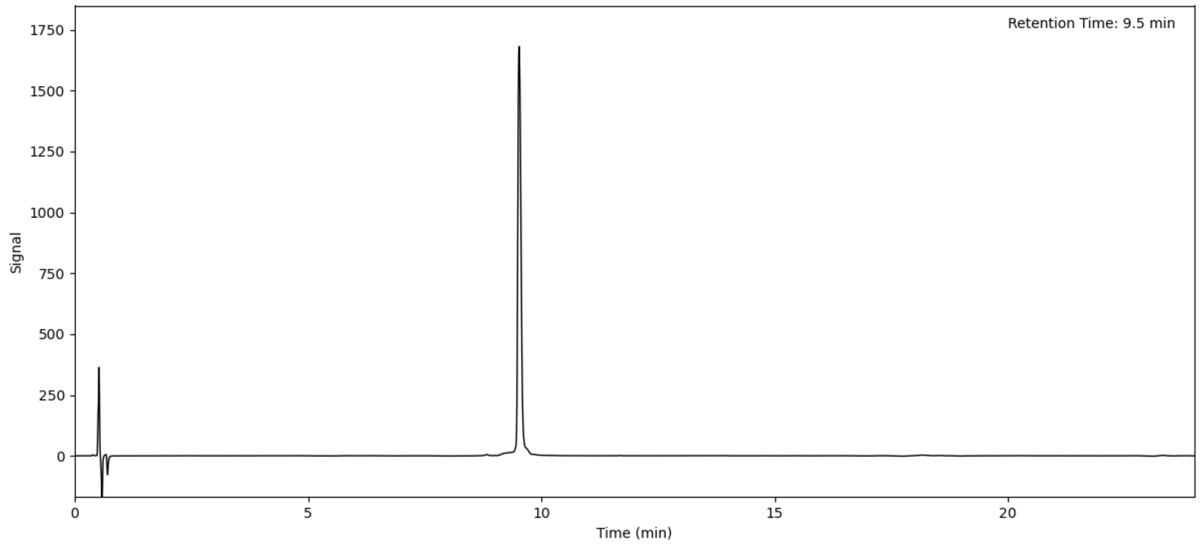

Peptide 42 (Method A)

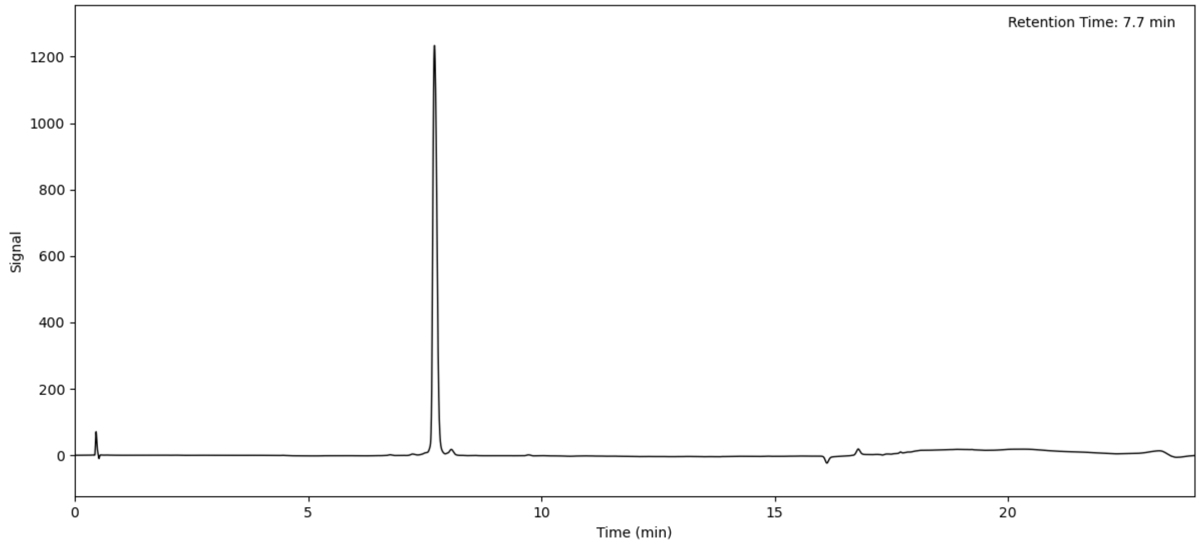

Peptide 43 (Method A)

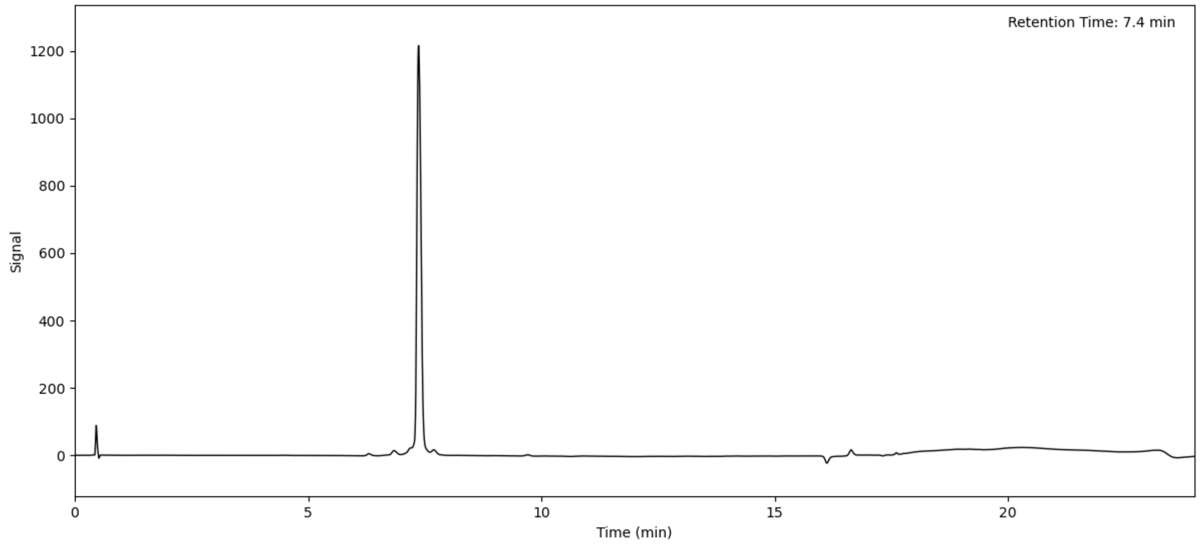

Peptide 44 (Method A)

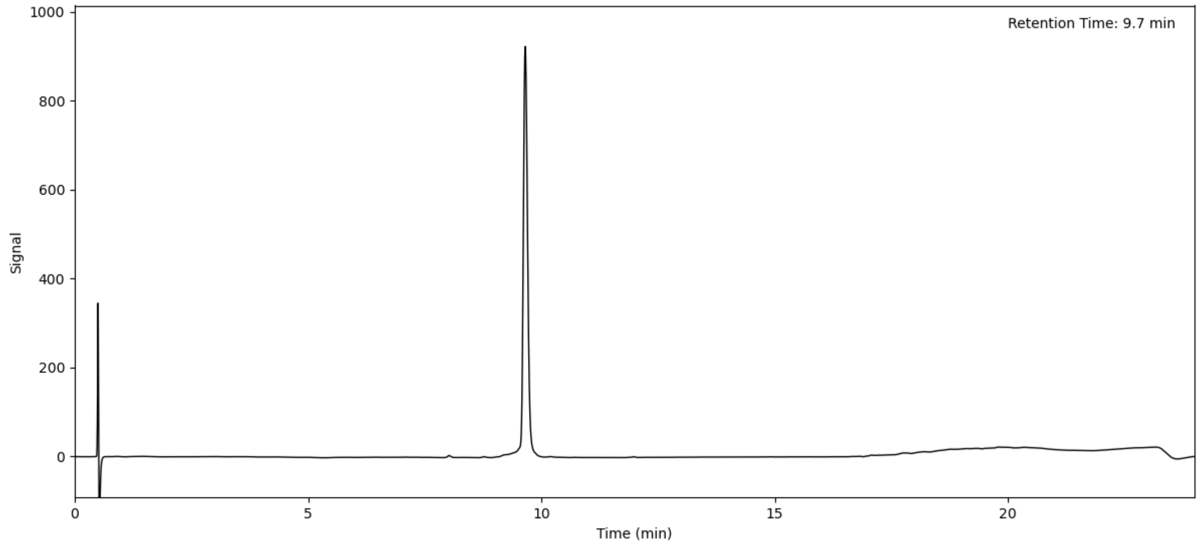

Peptide 45 (Method A)

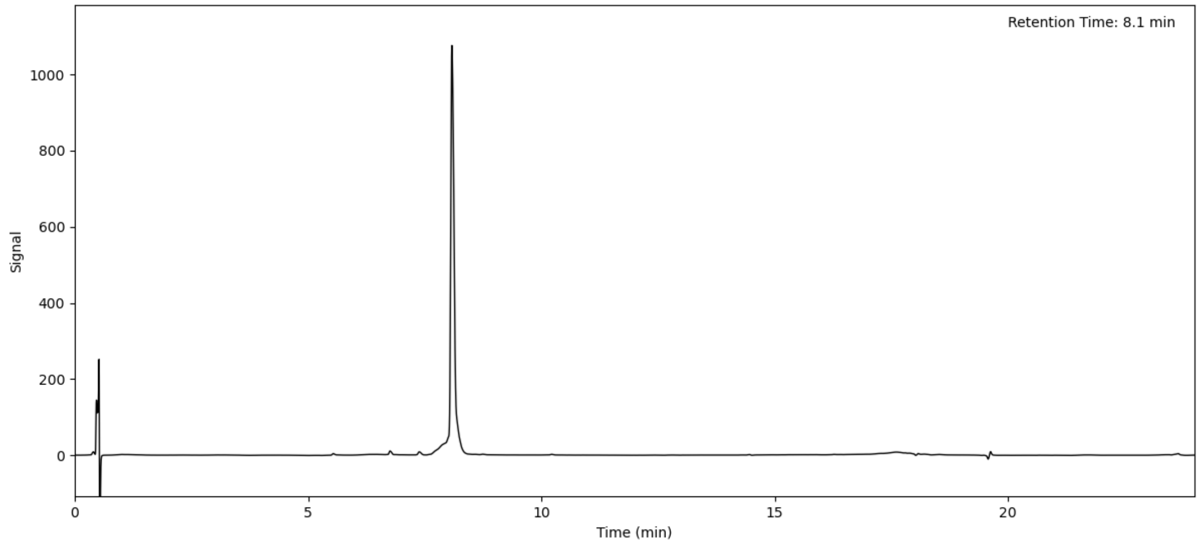

Peptide 46 (Method A)

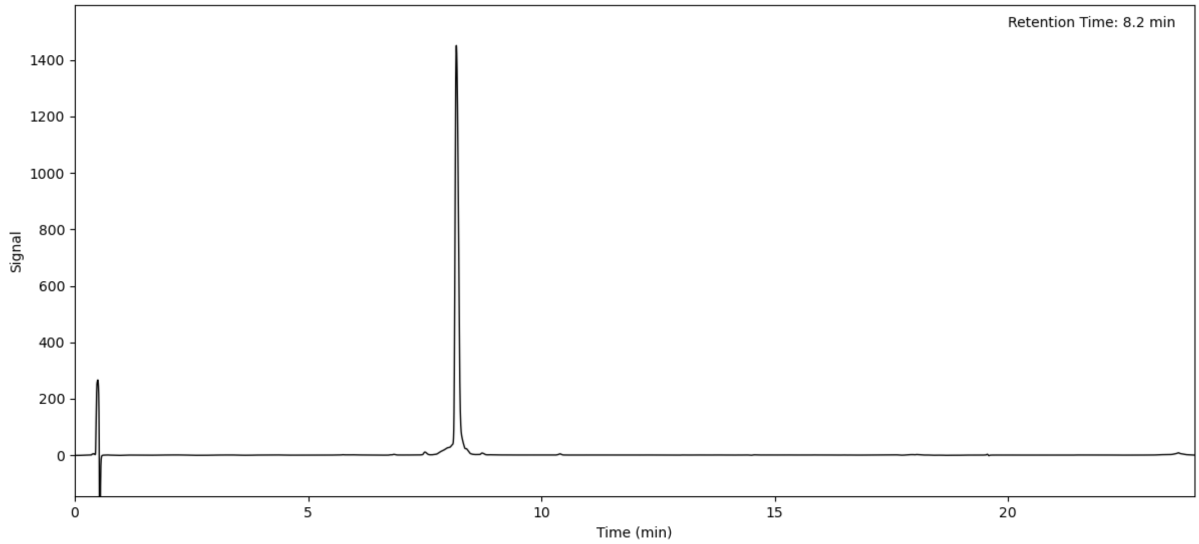

Peptide 47 (Method A)

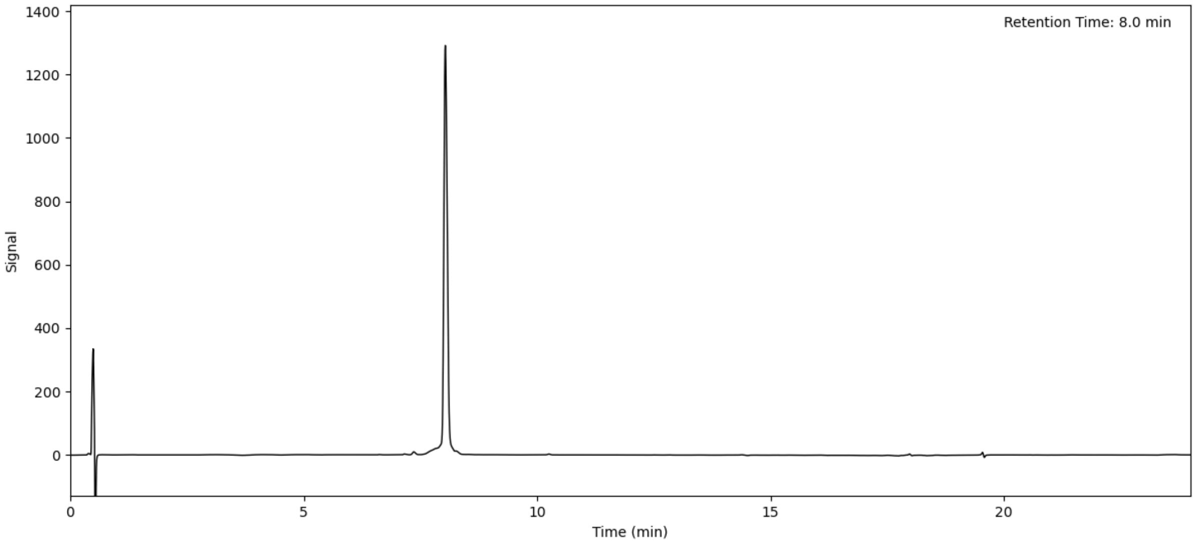

Peptide 48 (Method A)

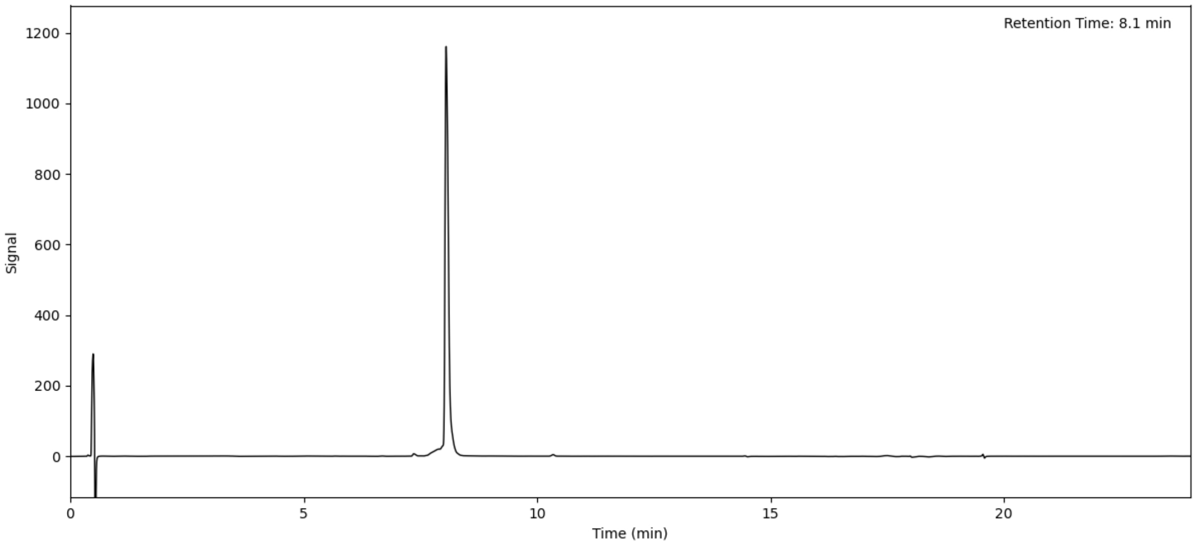

Peptide 49 (Method A)

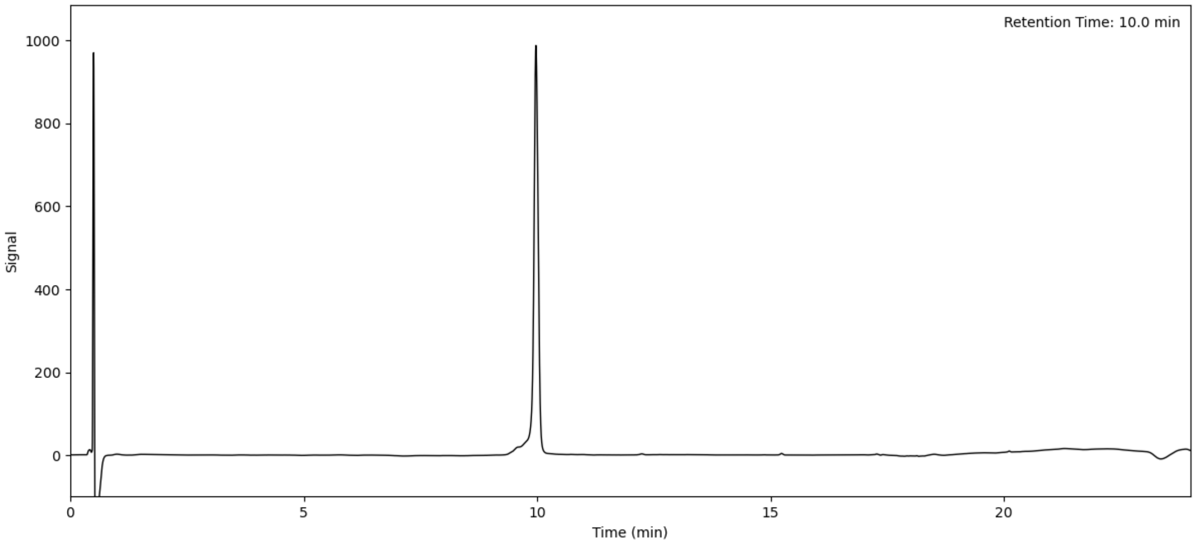

Peptide 50 (Method B)

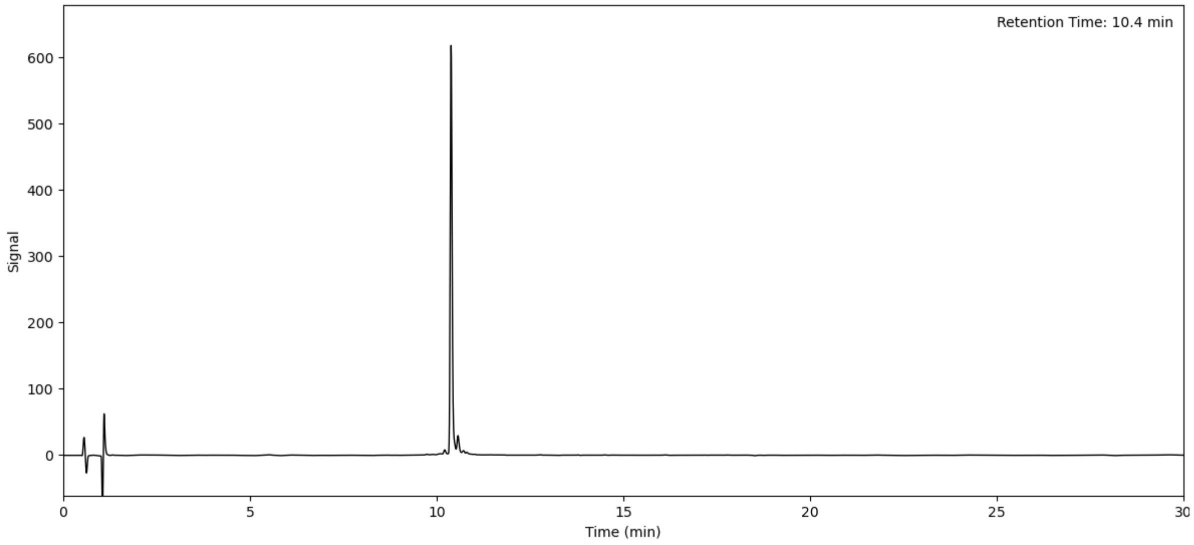

Peptide 51 (Method A)

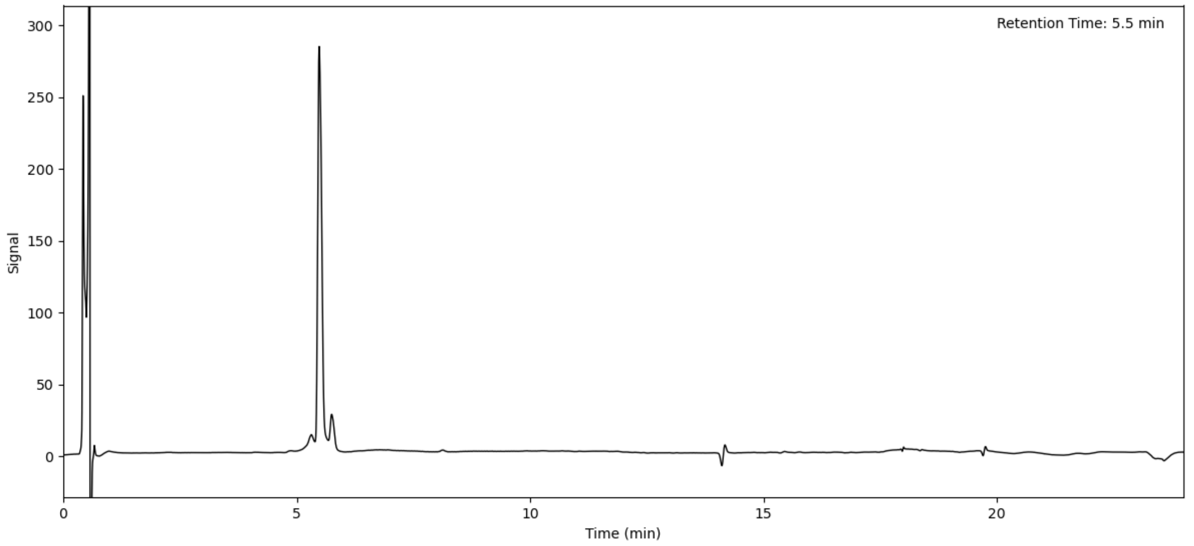

Peptide 52 (Method B)

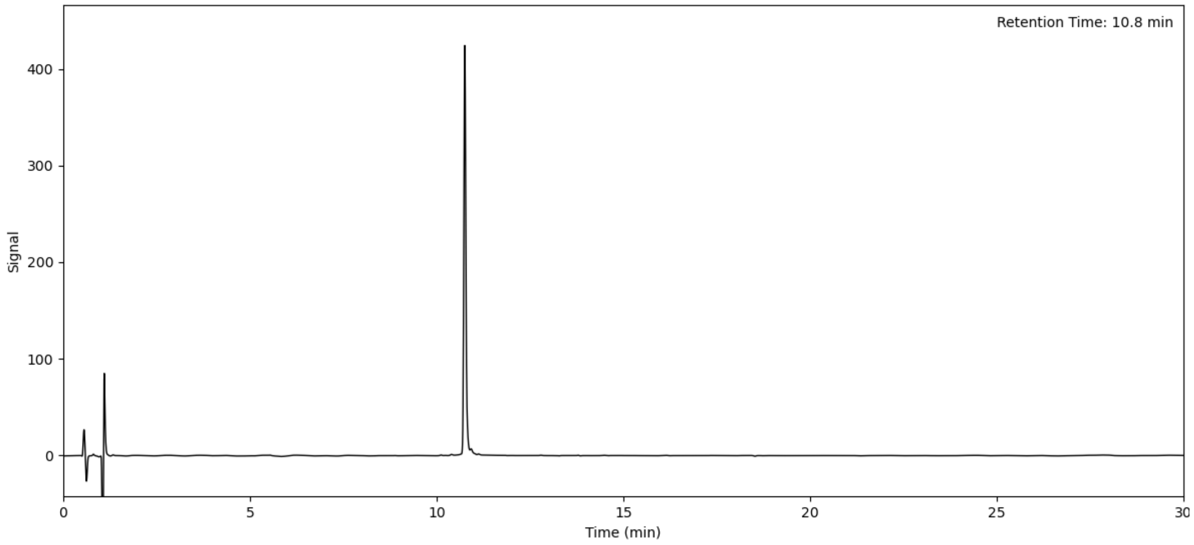

Peptide 53 (Method B)

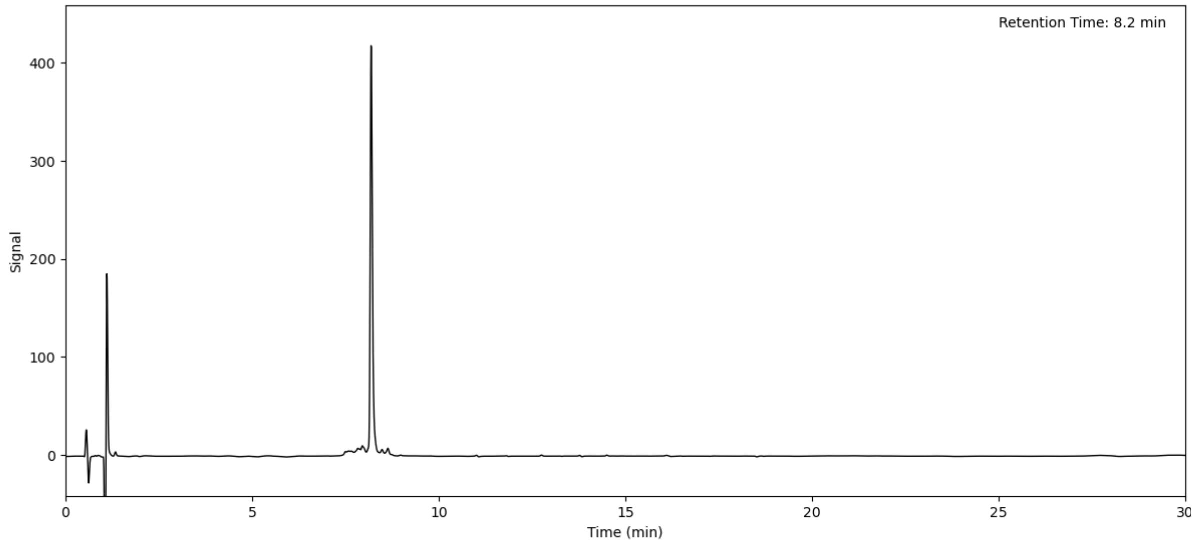

Peptide 54 (Method B)

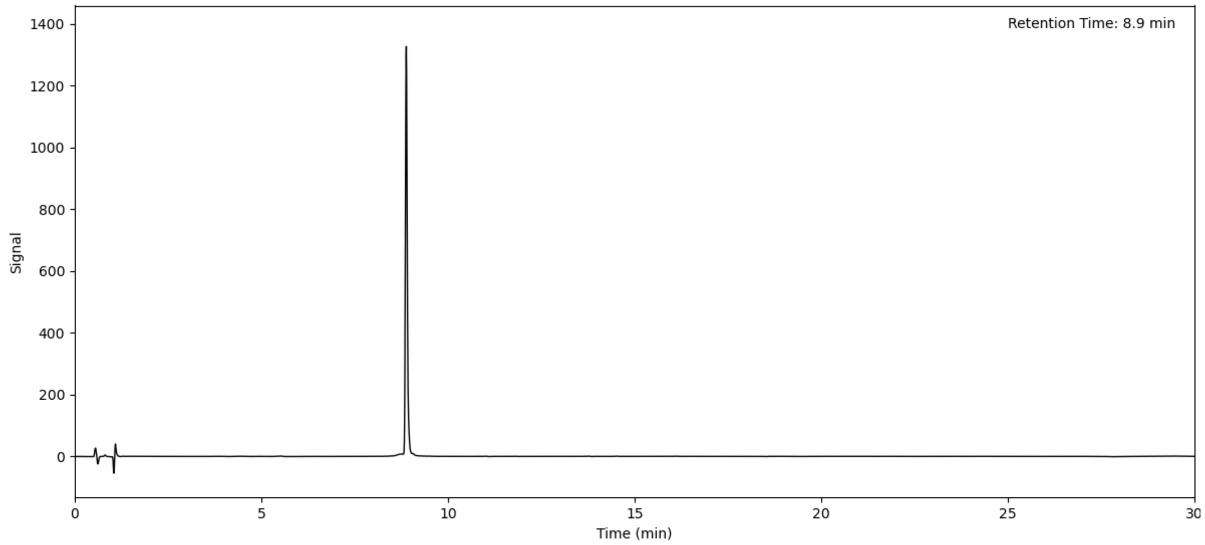

Peptide 55 (Method B)

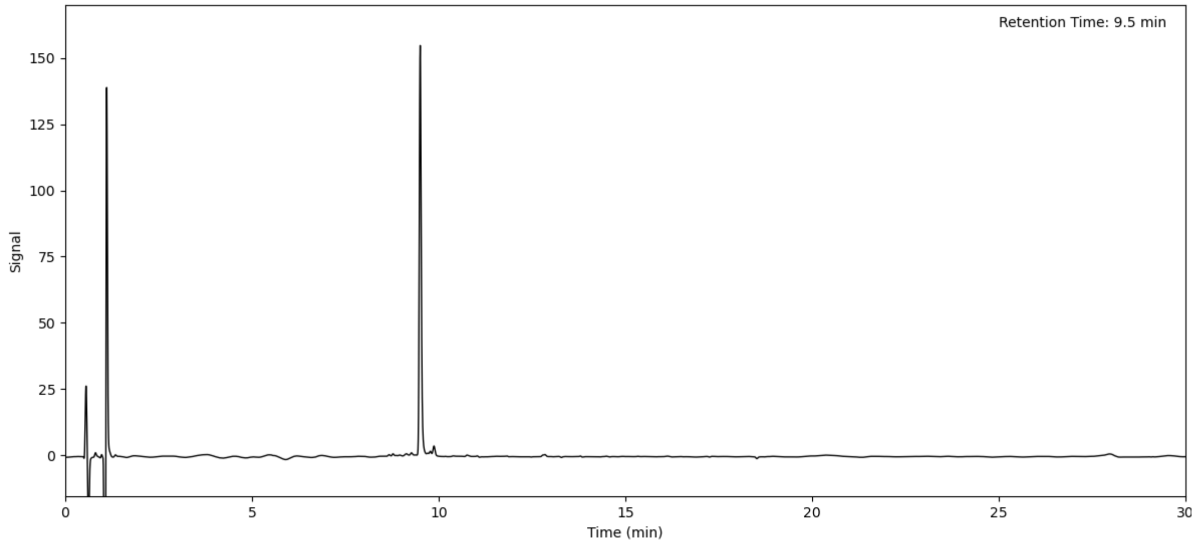

Peptide 56 (Method B)

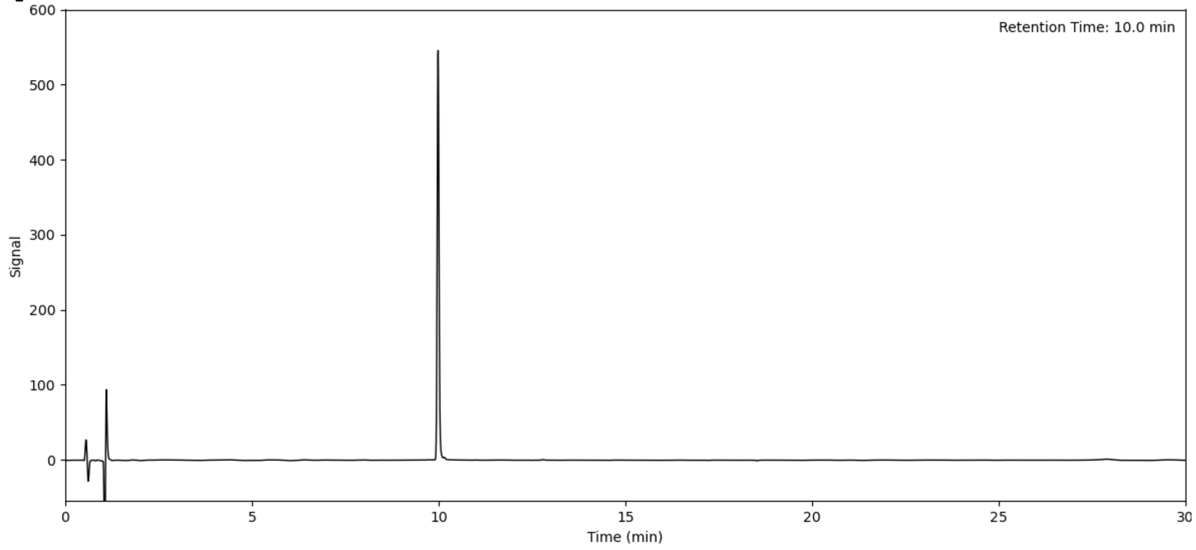

Peptide S1 (Method A)

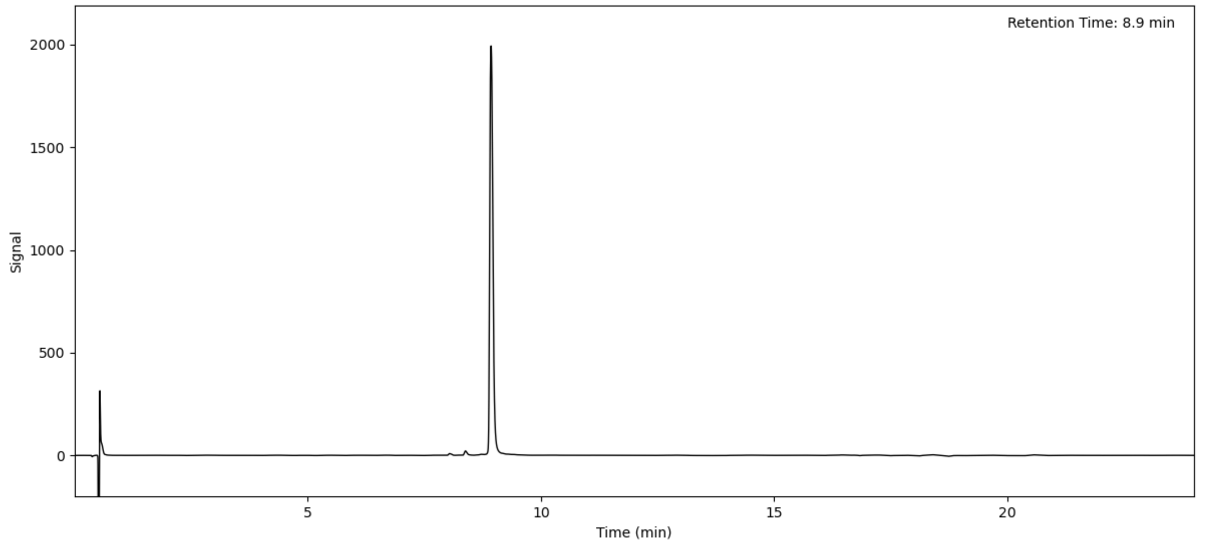

Peptide S2 (Method A)

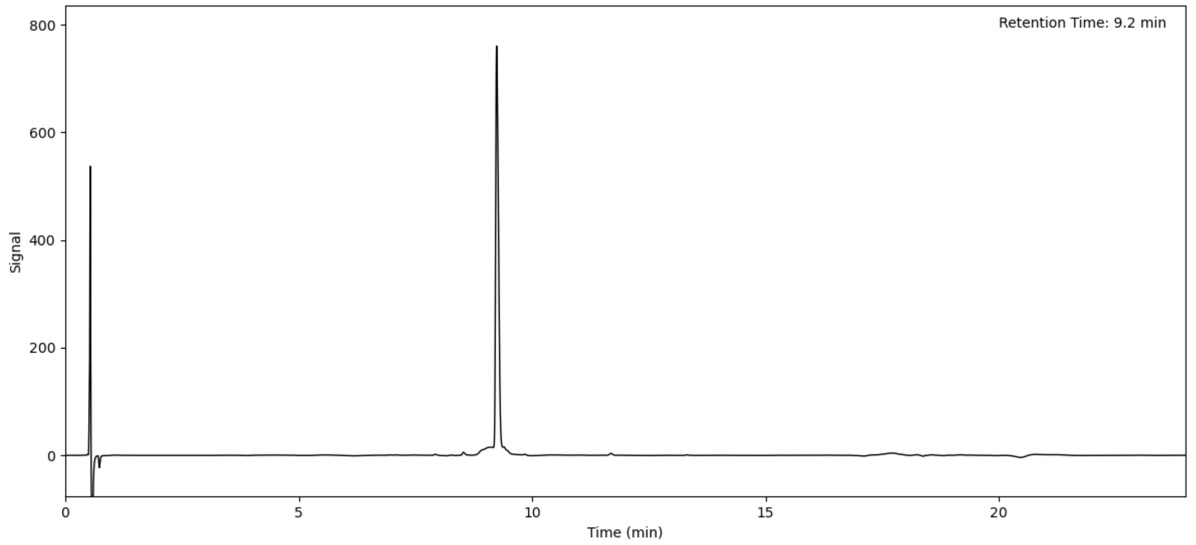

Peptide S3 (Method A)

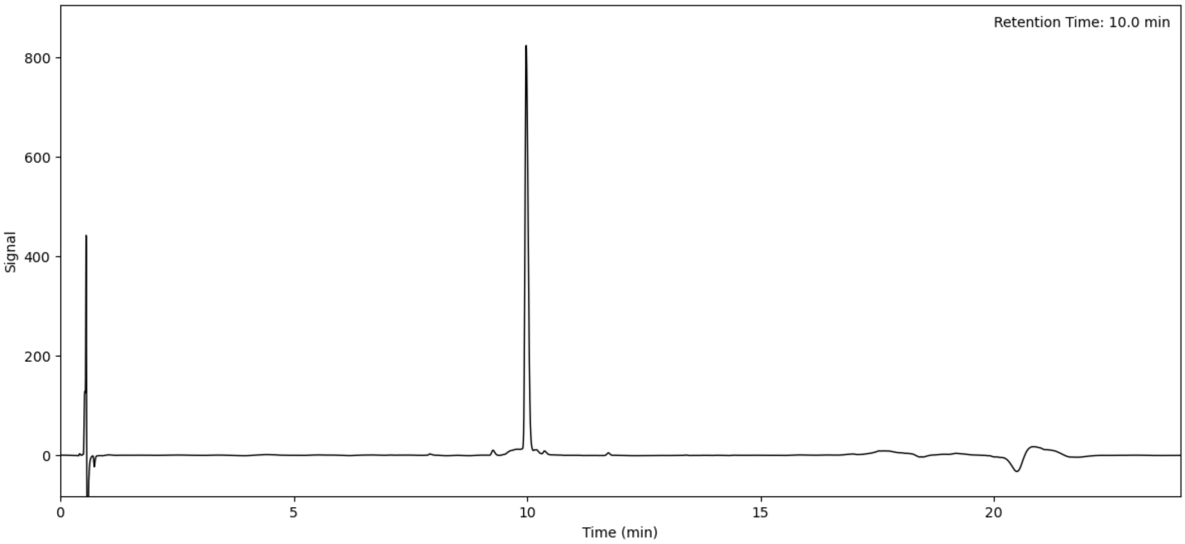

Peptide S4 (Method A)

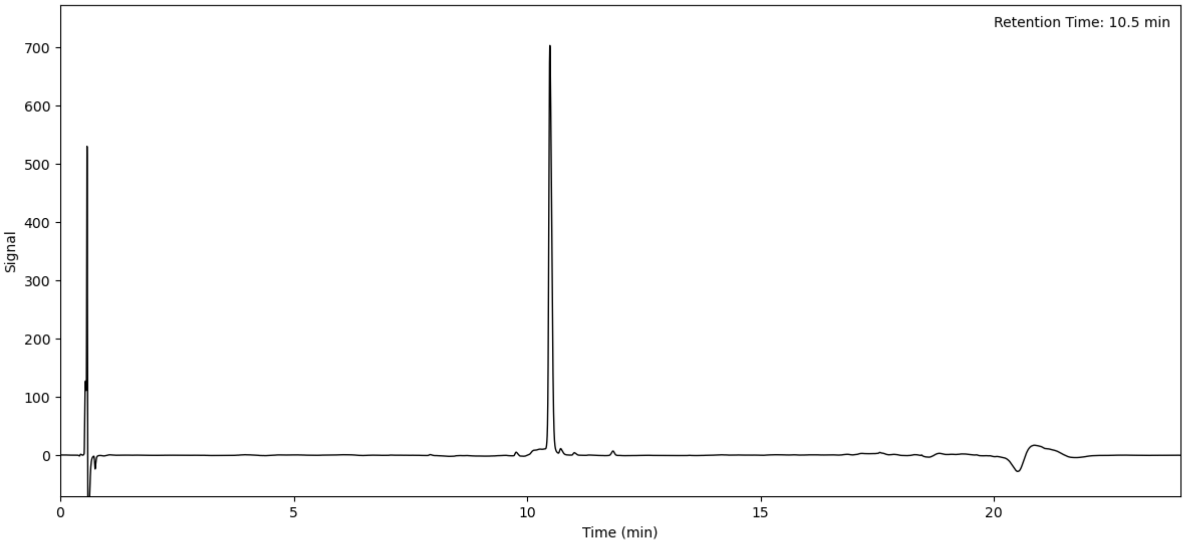

Peptide S5 (Method A)

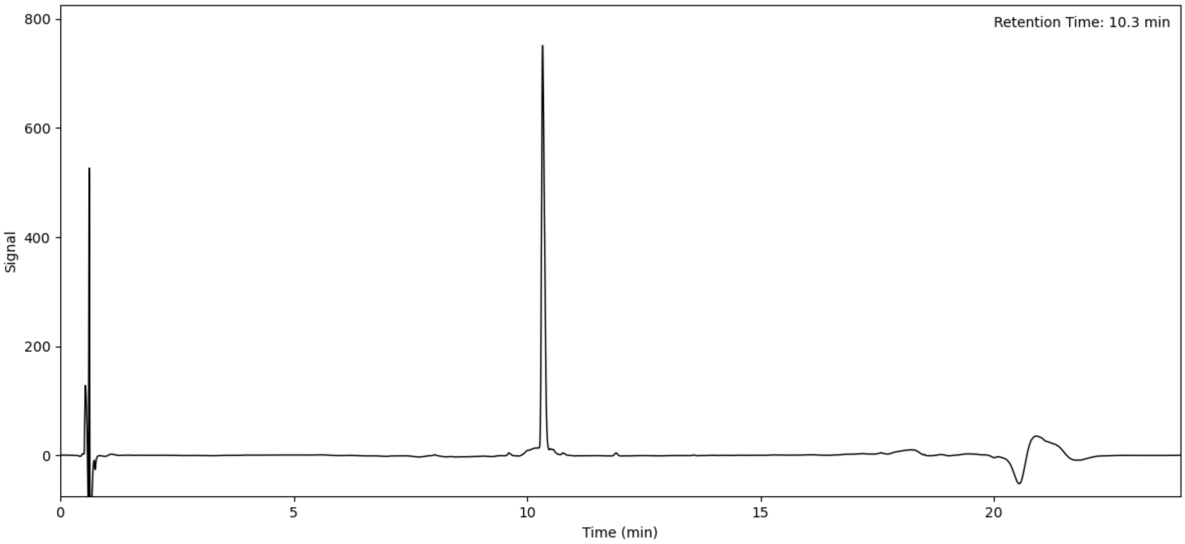

Peptide S6 (Method A)

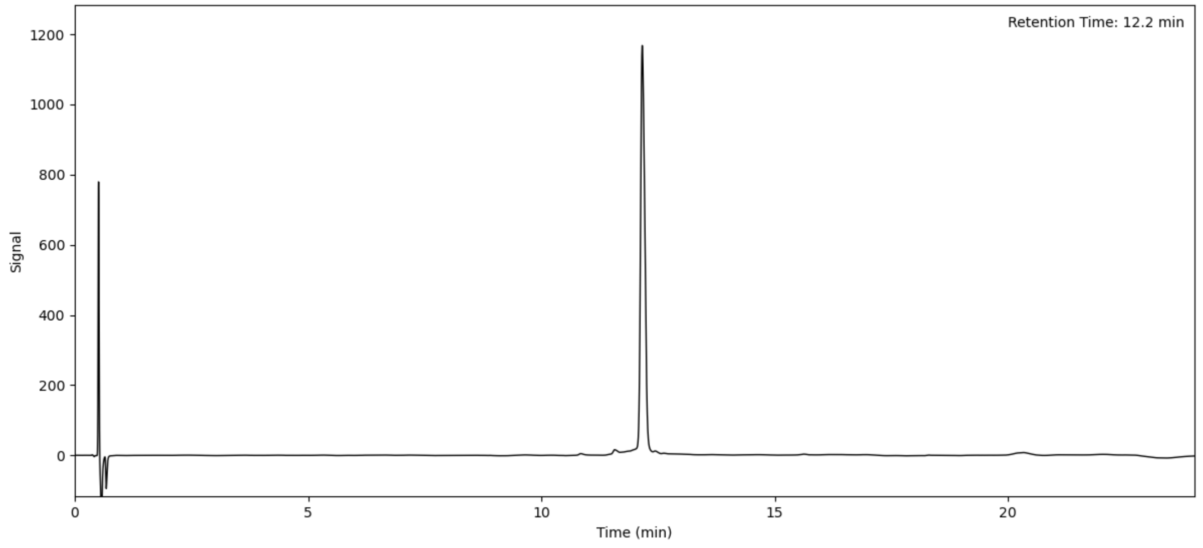

Peptide S7 (Method A)

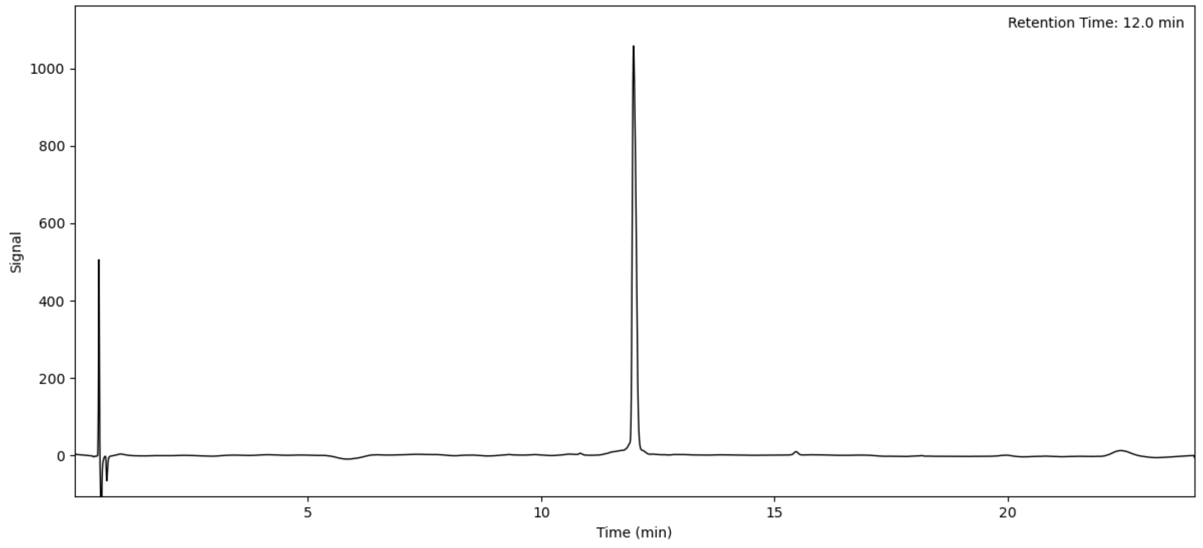

Peptide S8 (Method A)

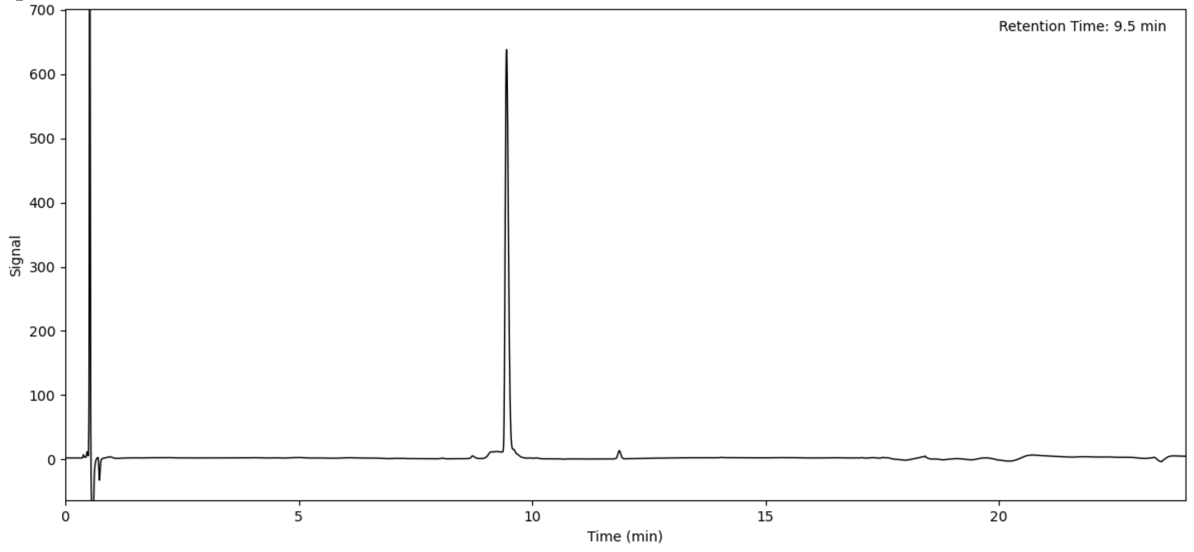

Peptide S9 (Method A)

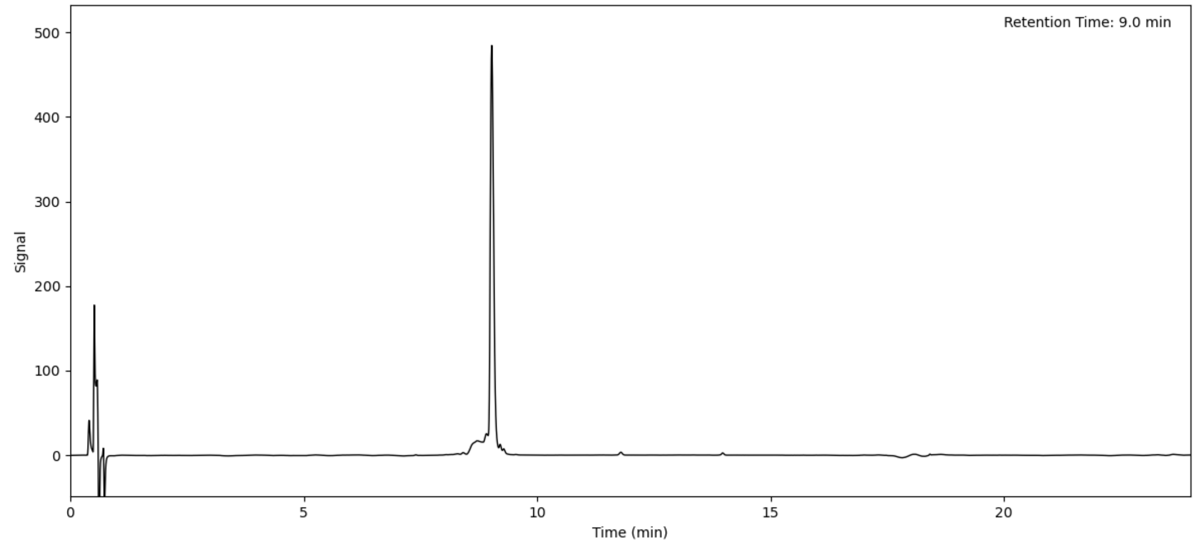

Peptide S10 (Method A)

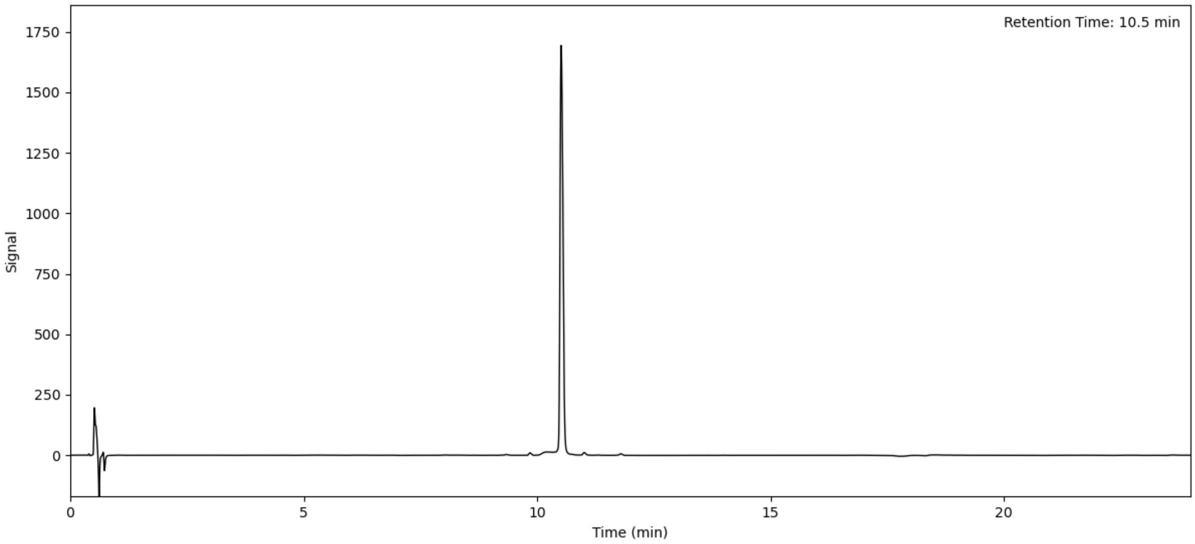

Peptide S11 (Method A)

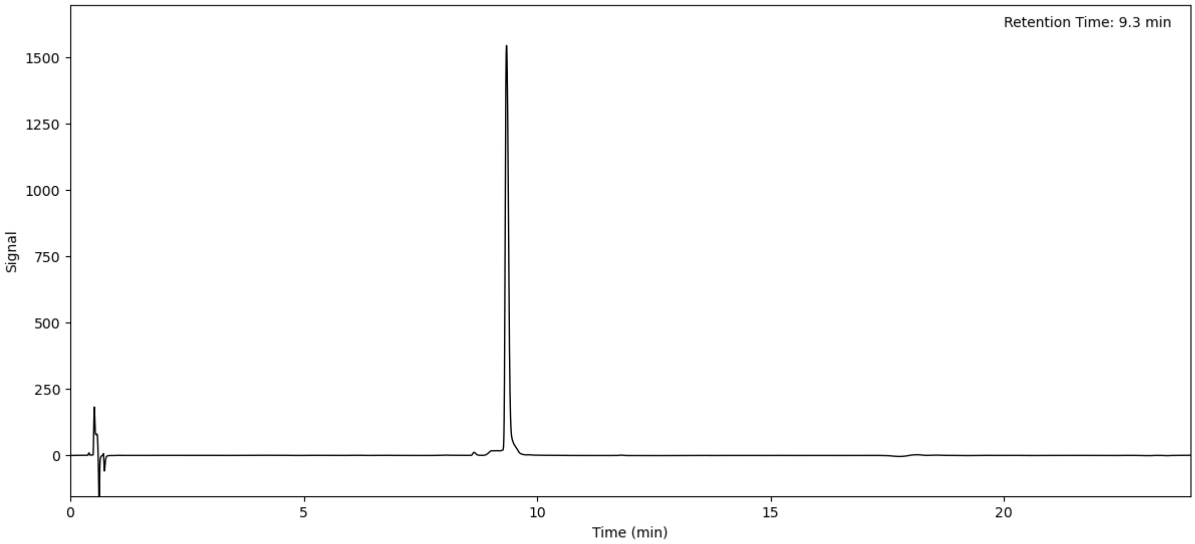

Peptide S12 (Method A)

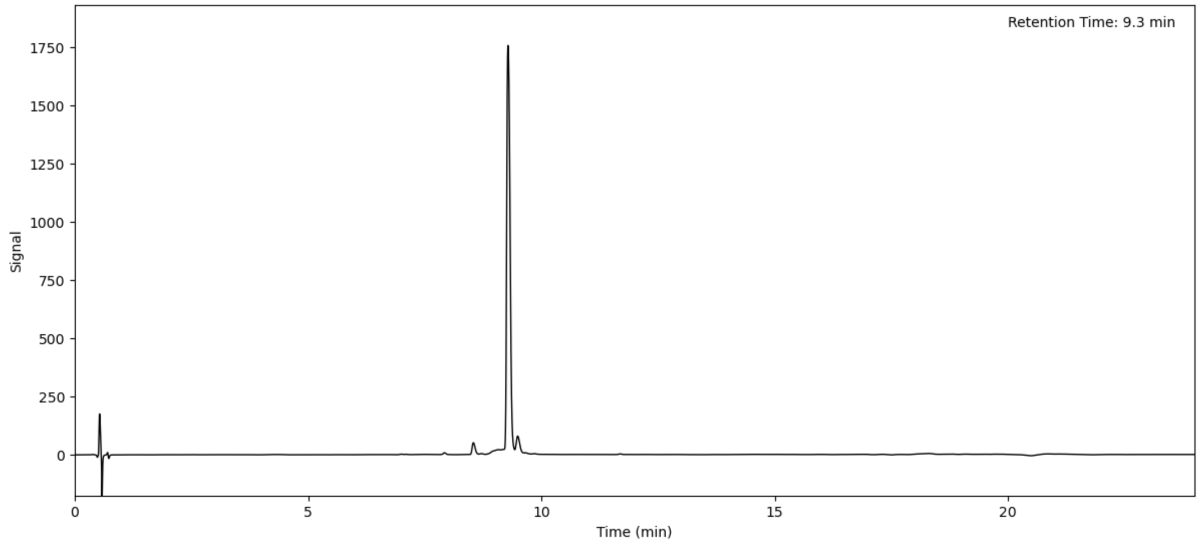

Peptide S13 (Method A)

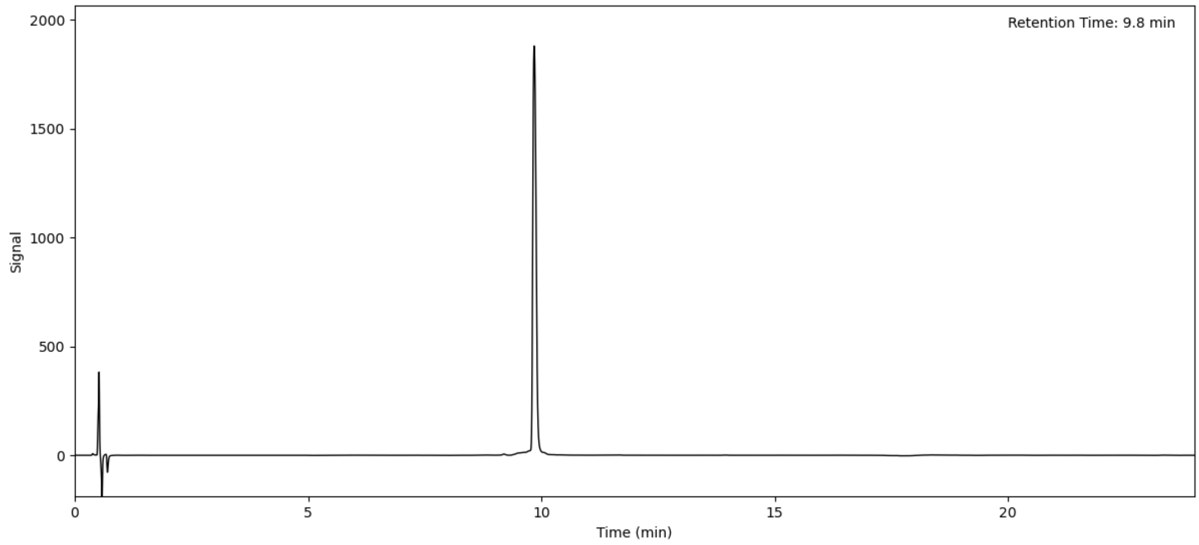

Peptide S14 (Method A)

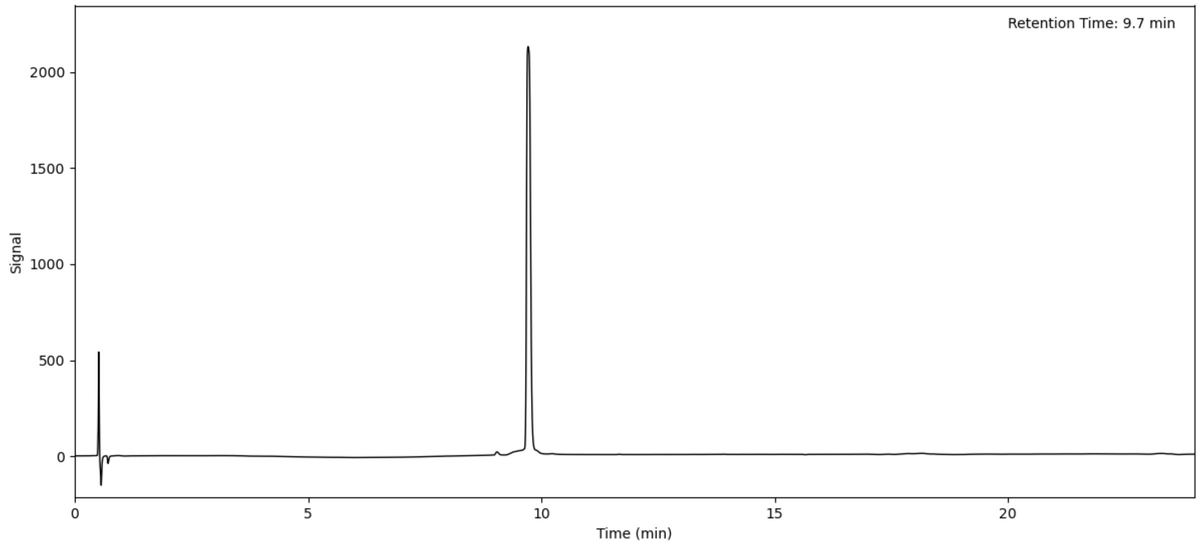

Peptide S15 (Method A)

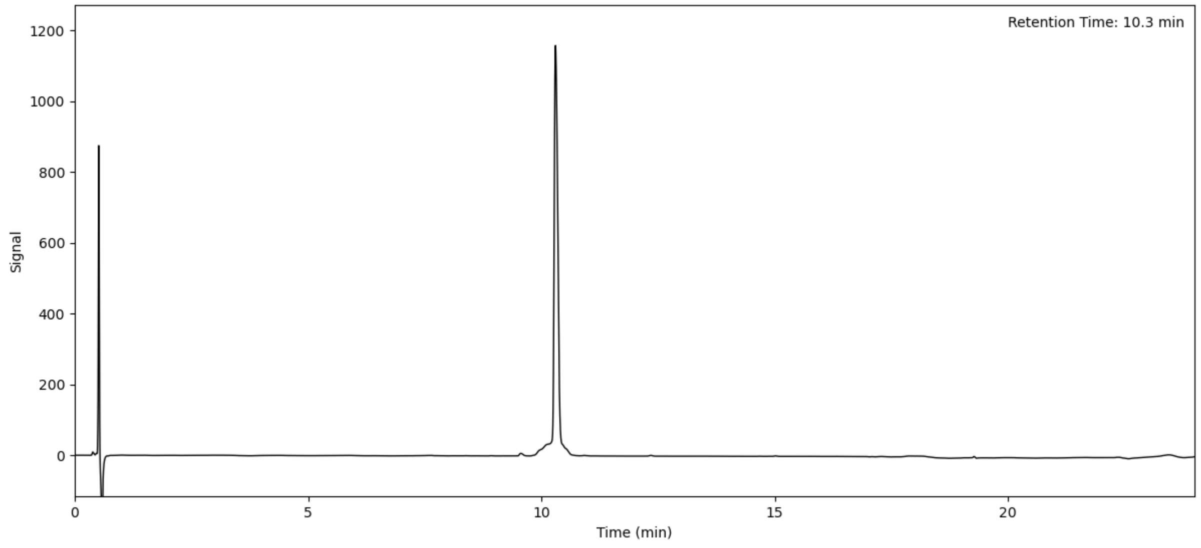

Peptide S16 (Method A)

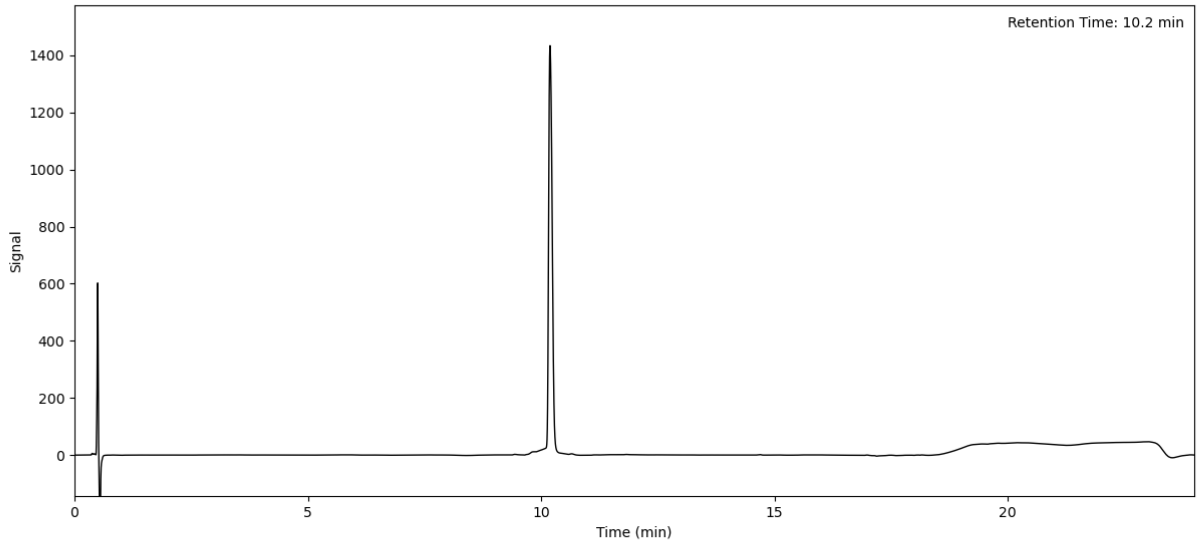

Peptide S17 (Method A)

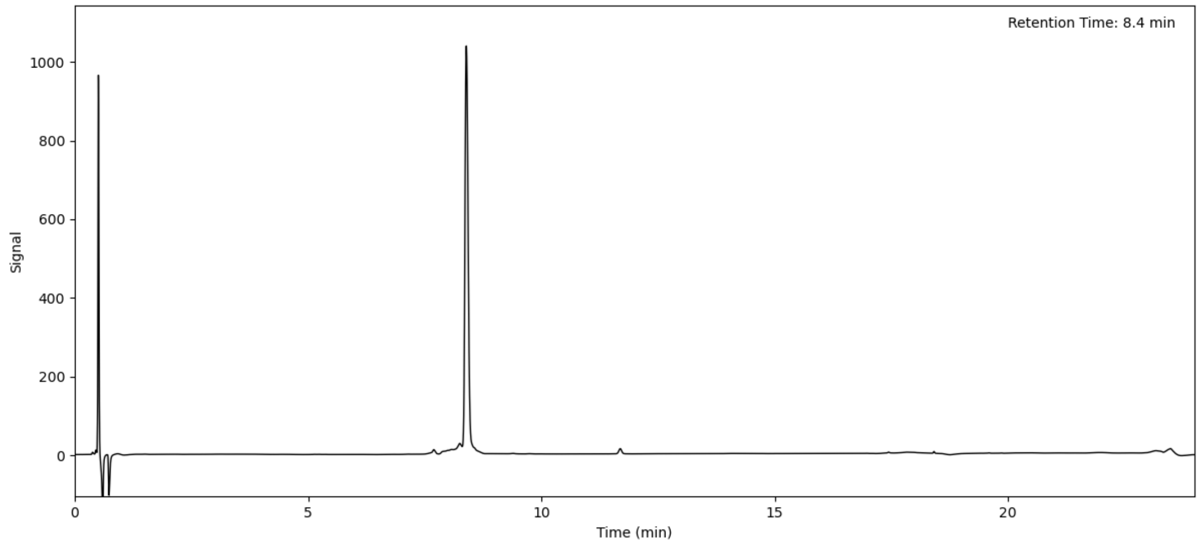

Peptide S18 (Method A)

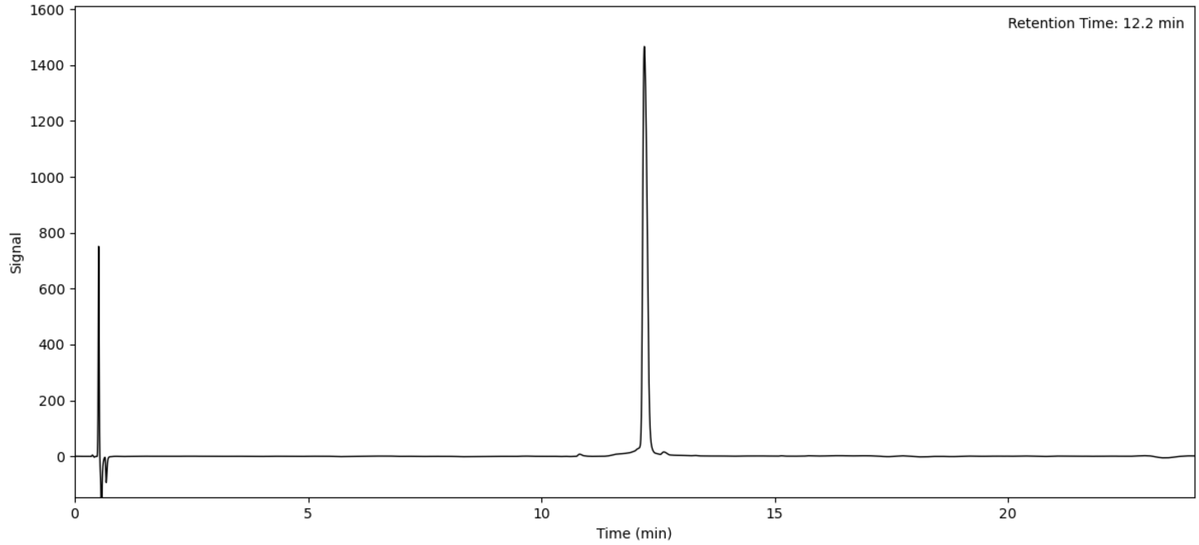

Peptide S19 (Method A)

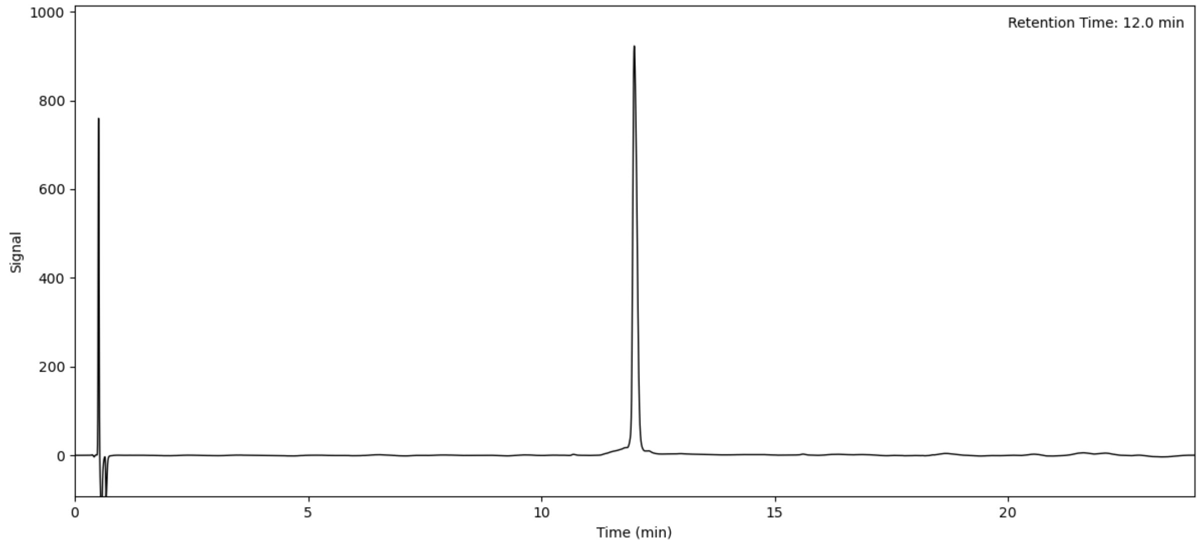

Peptide S20 (Method A)

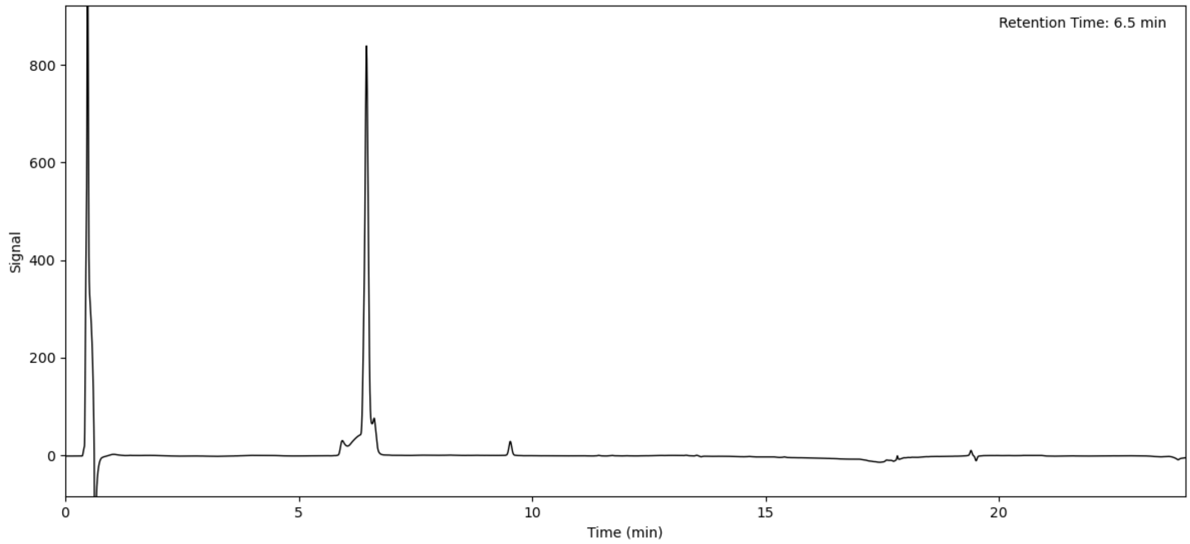

Peptide S21 (Method A)

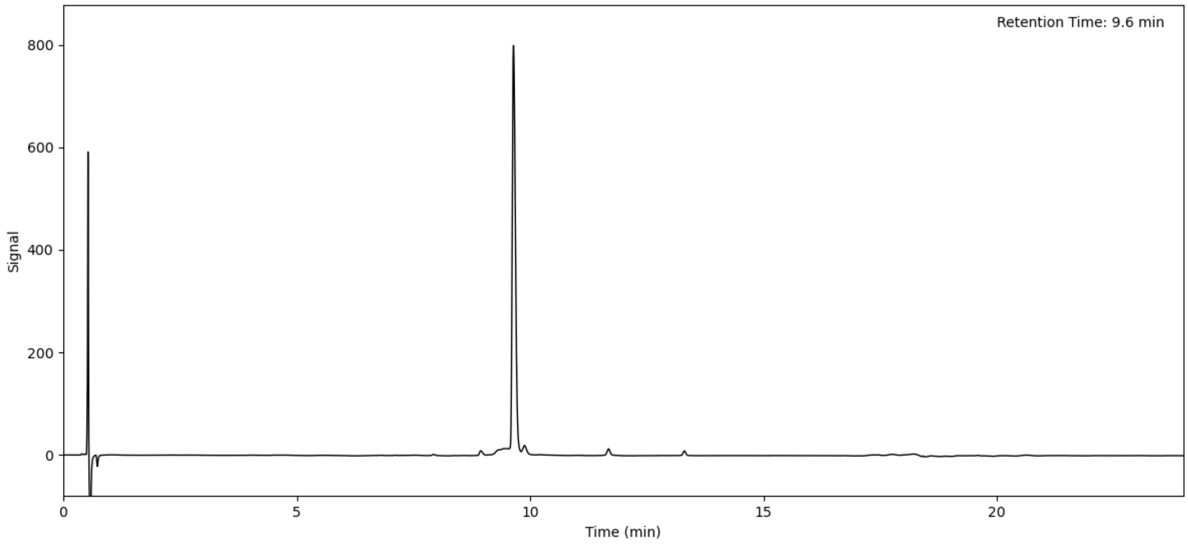

Peptide S22 (Method A)

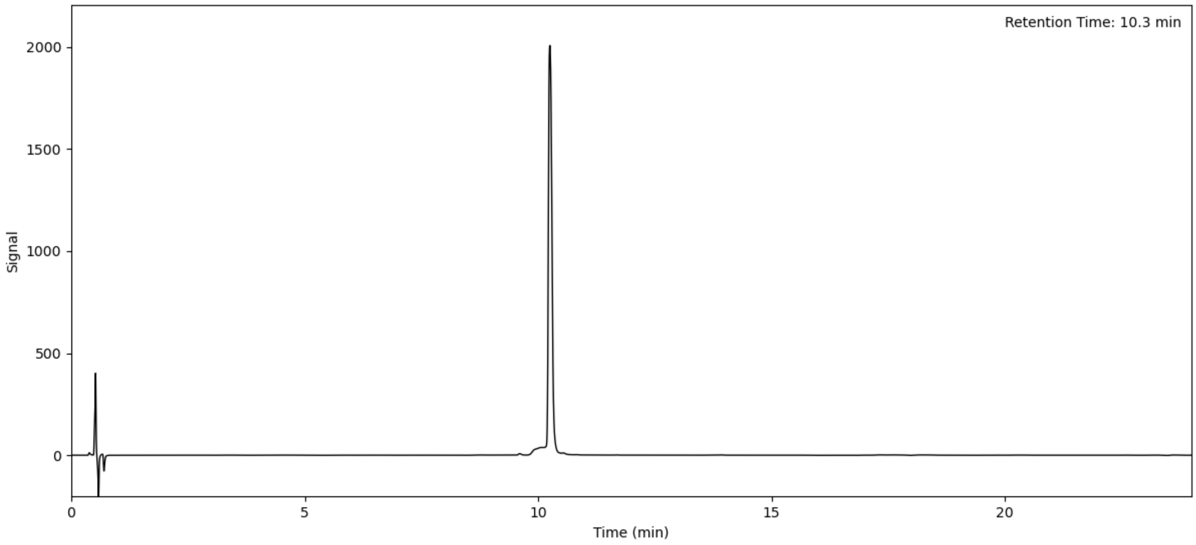

Peptide S23 (Method A)

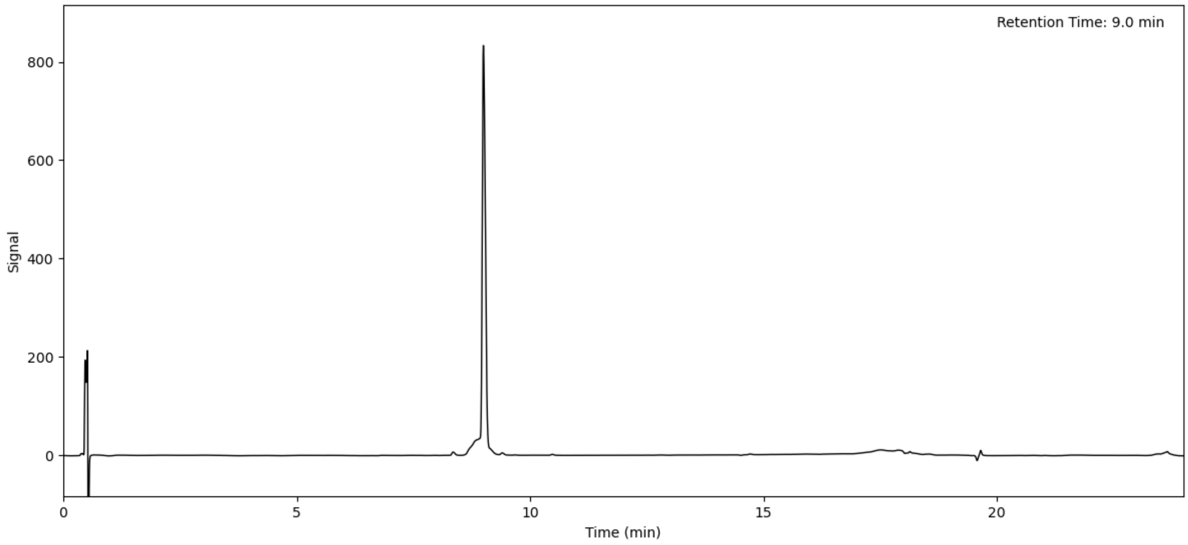

Peptide S24 (Method A)

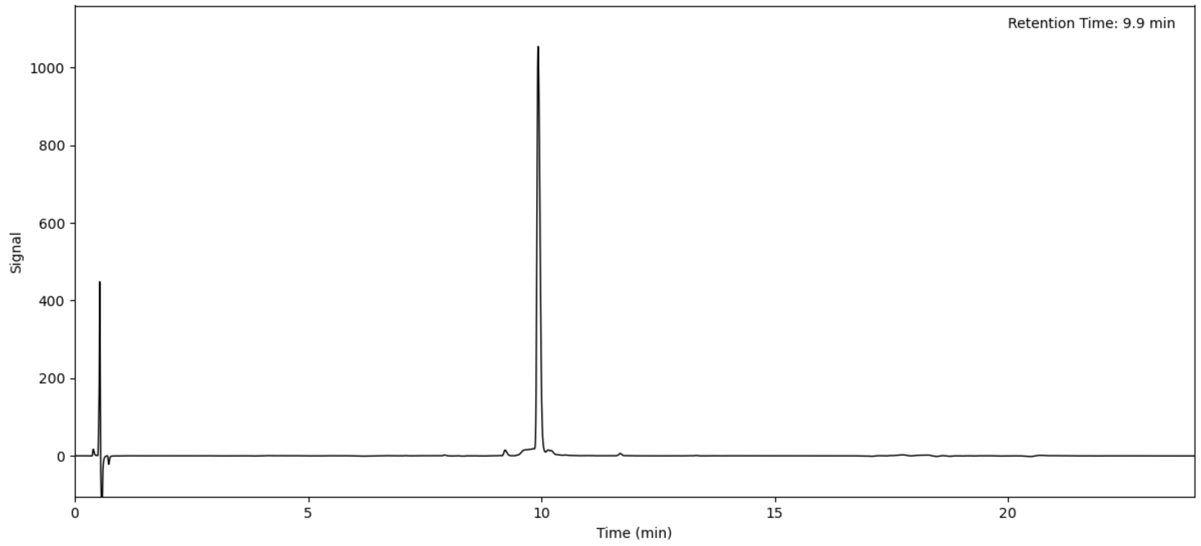

Peptide S25 (Method A)

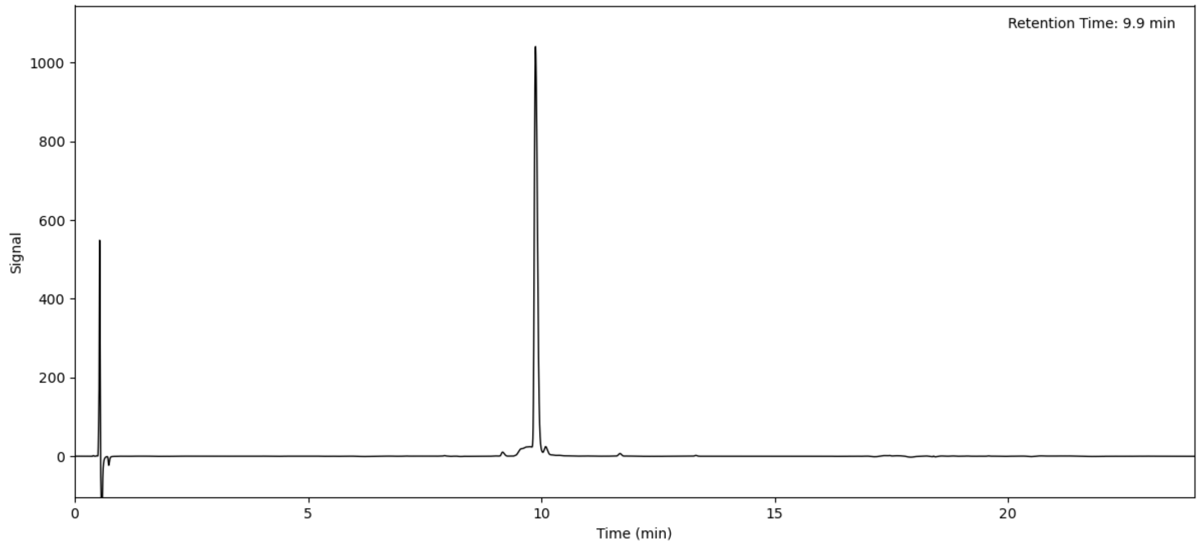

Peptide S26 (Method A)

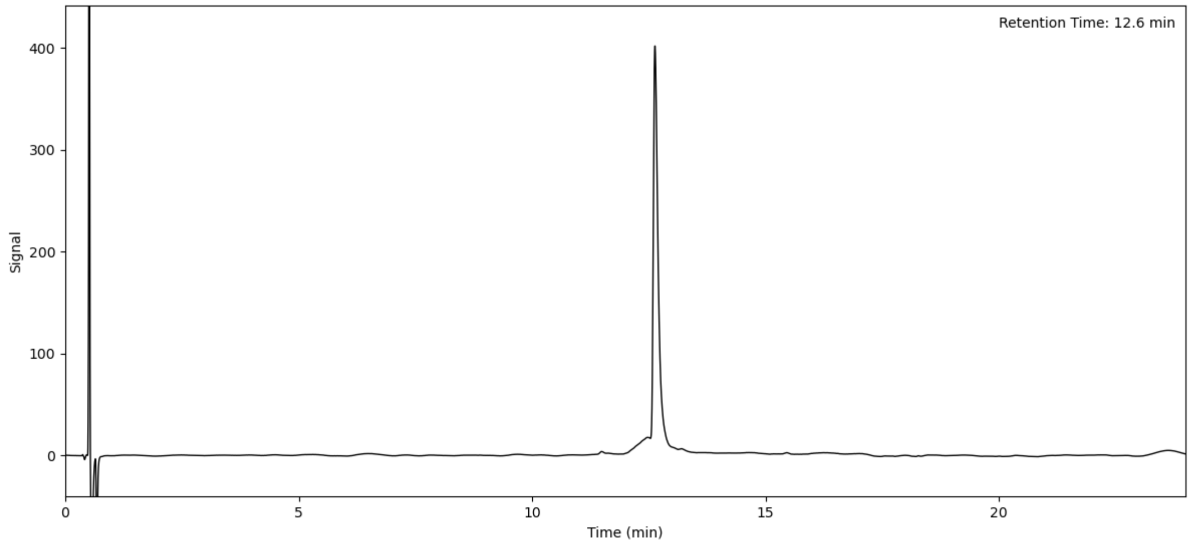

Peptide S27 (Method A)

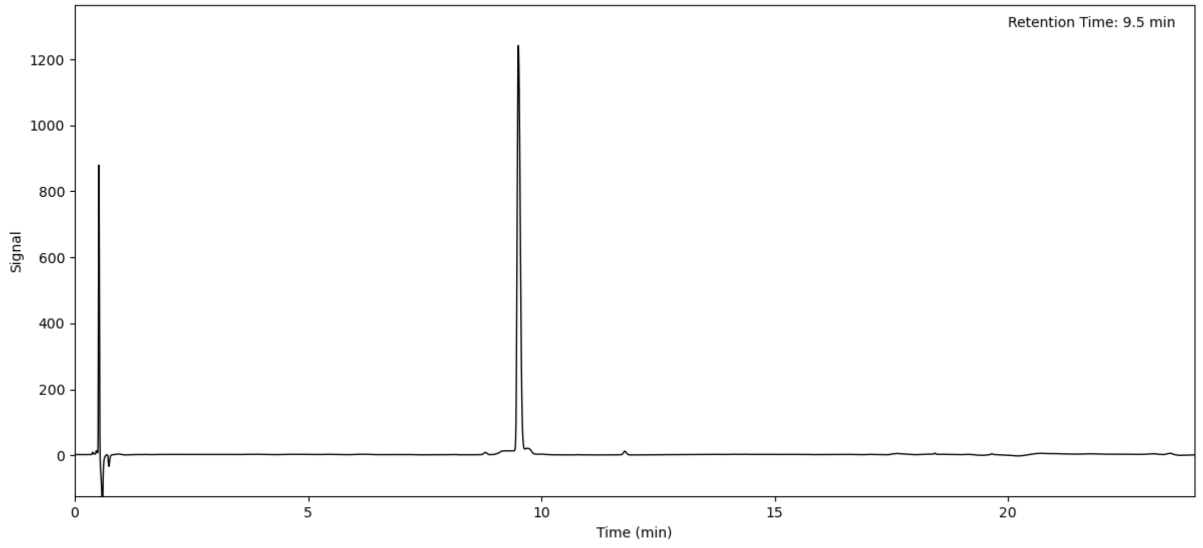

Peptide S28 (Method A)

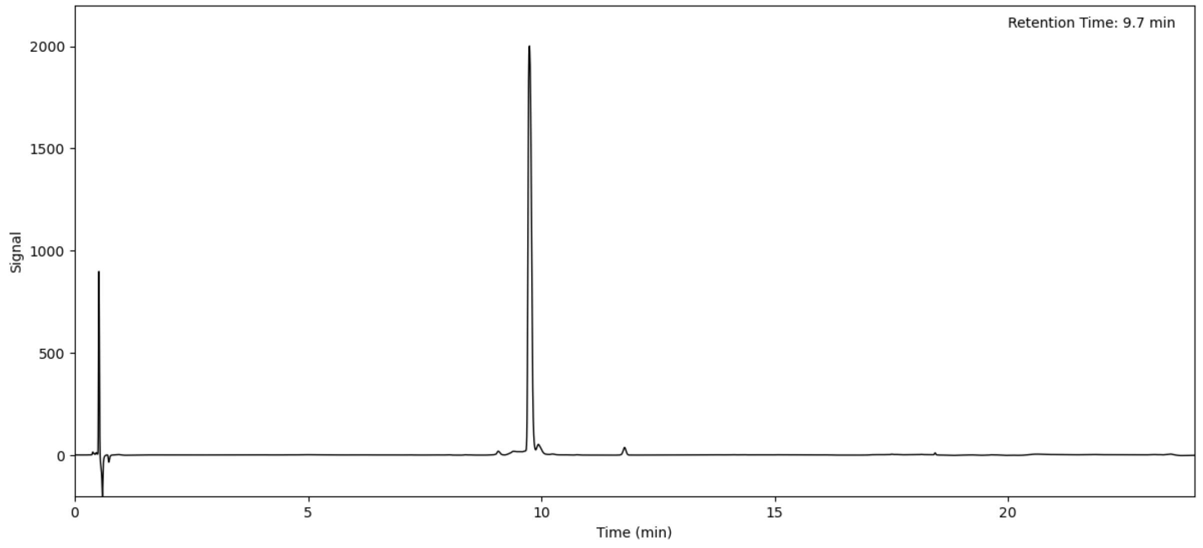

Peptide S29 (Method A)

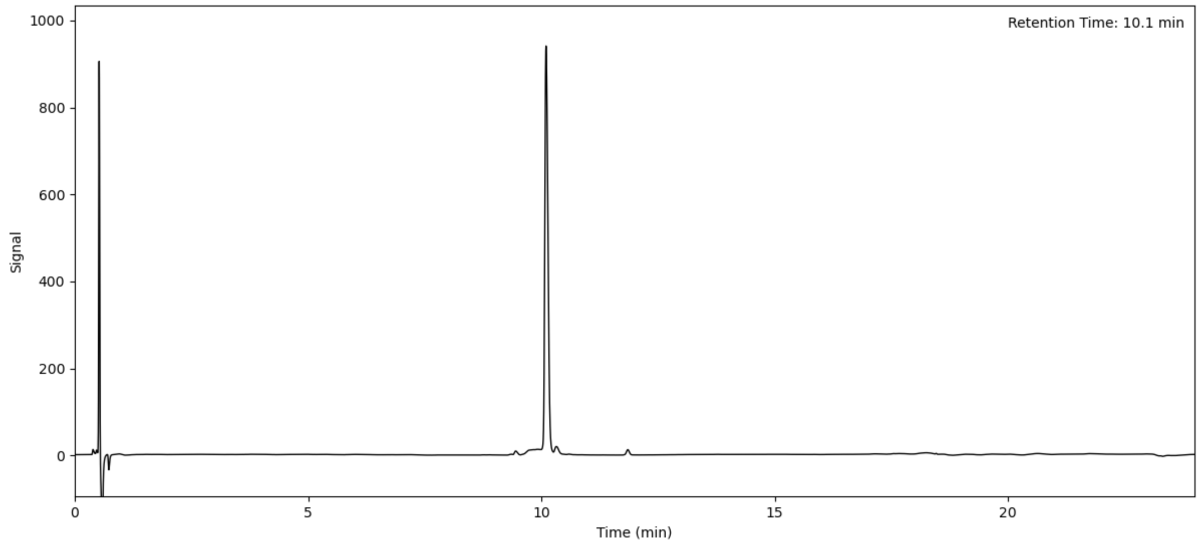

Peptide S30 (Method A)

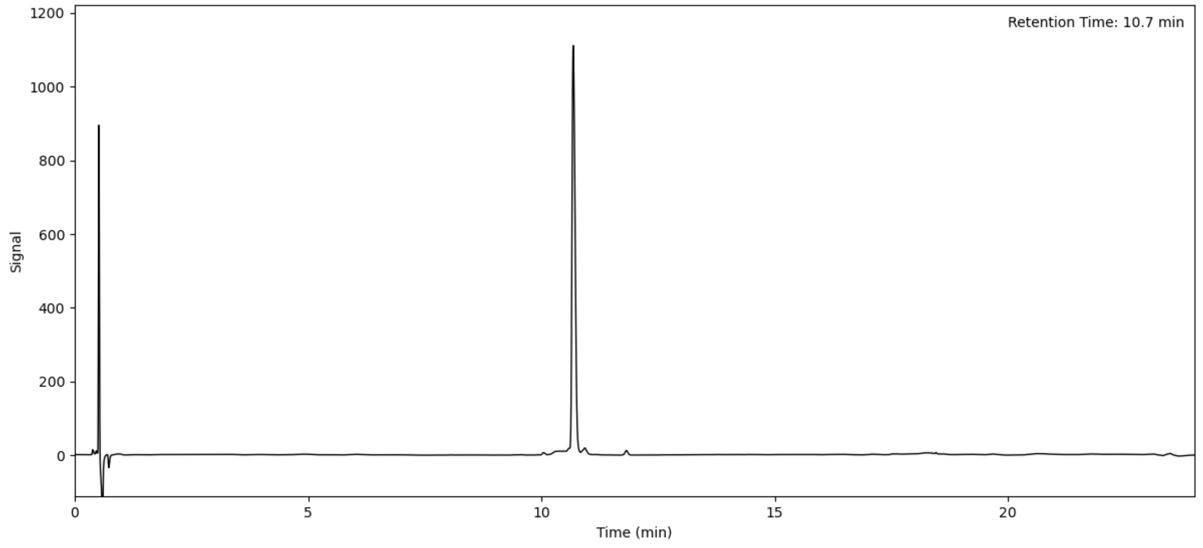

Peptide S31 (Method A)

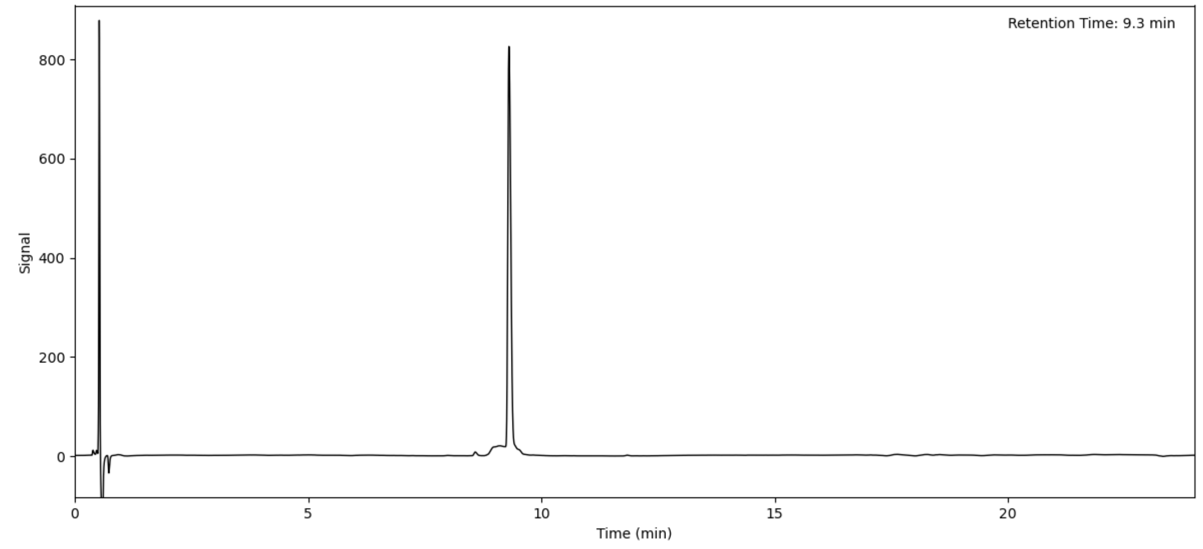

Peptide S32 (Method A)

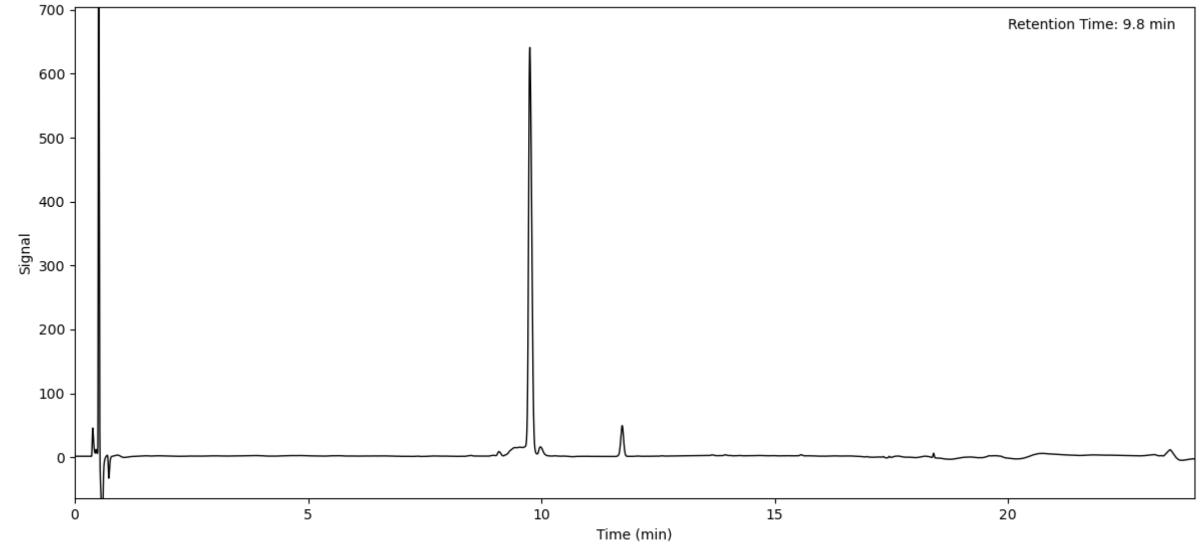

Peptide S33 (Method A)

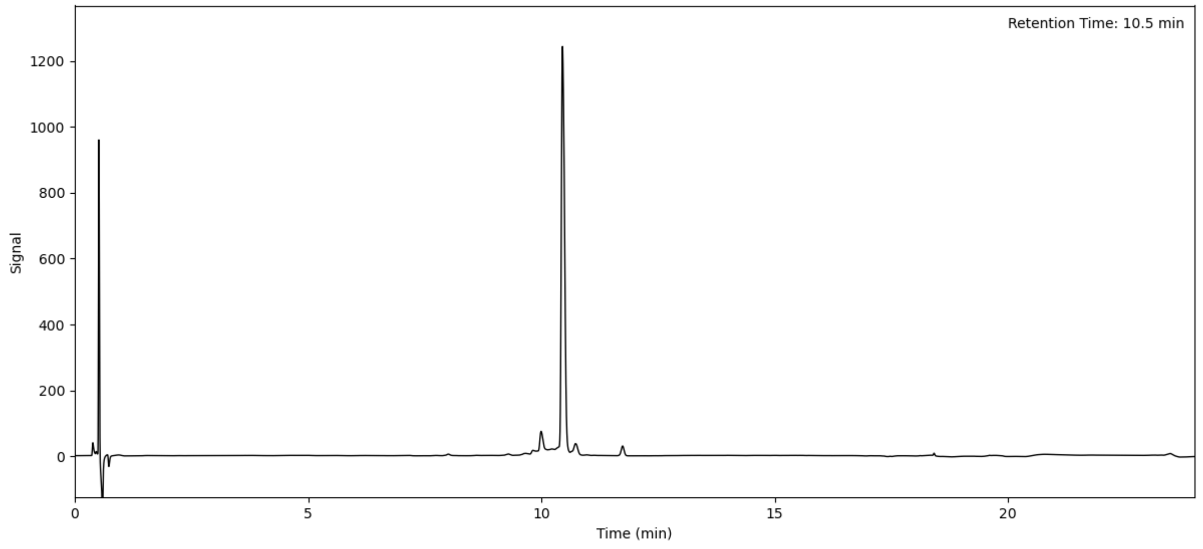

Peptide S34 (Method A)

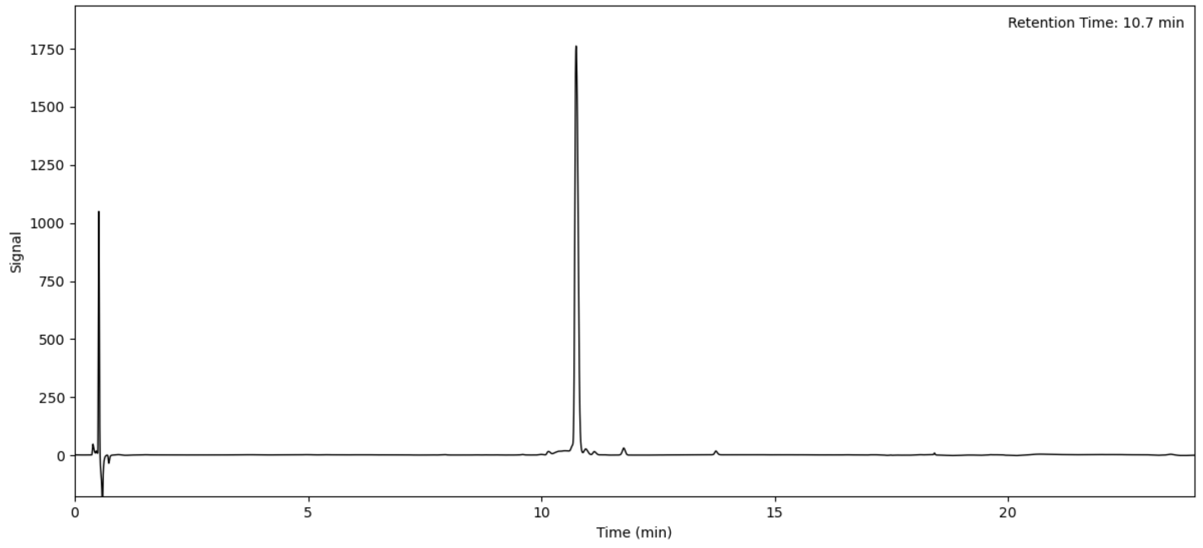

H4 histone tail peptide(the MTase Glo substrate) (Method A)

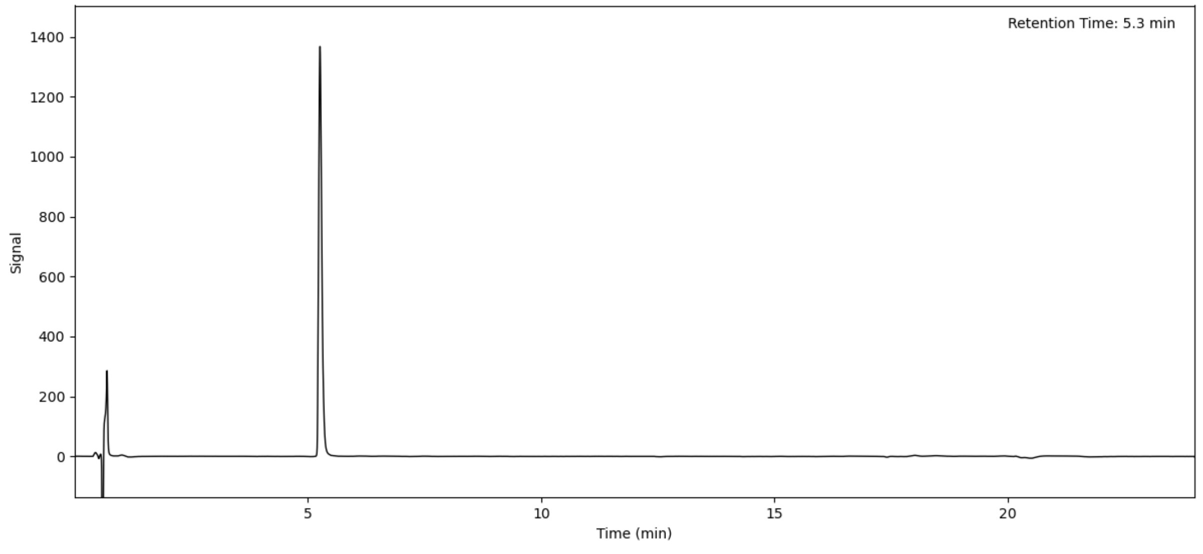

Supplement: Supplementary file 1 — jm2c01273_si_001.pdf [file jm2c01273_si_001.pdf]
